# Supplementary figures and images for: Behavioral correlates of cortical semantic representations modeled by word vectors
Source: PLoS Comput Biol. 2021 Jun 23;17(6):e1009138. doi: 10.1371/journal.pcbi.1009138 (PMC8260002; doi:10.1371/journal.pcbi.1009138)

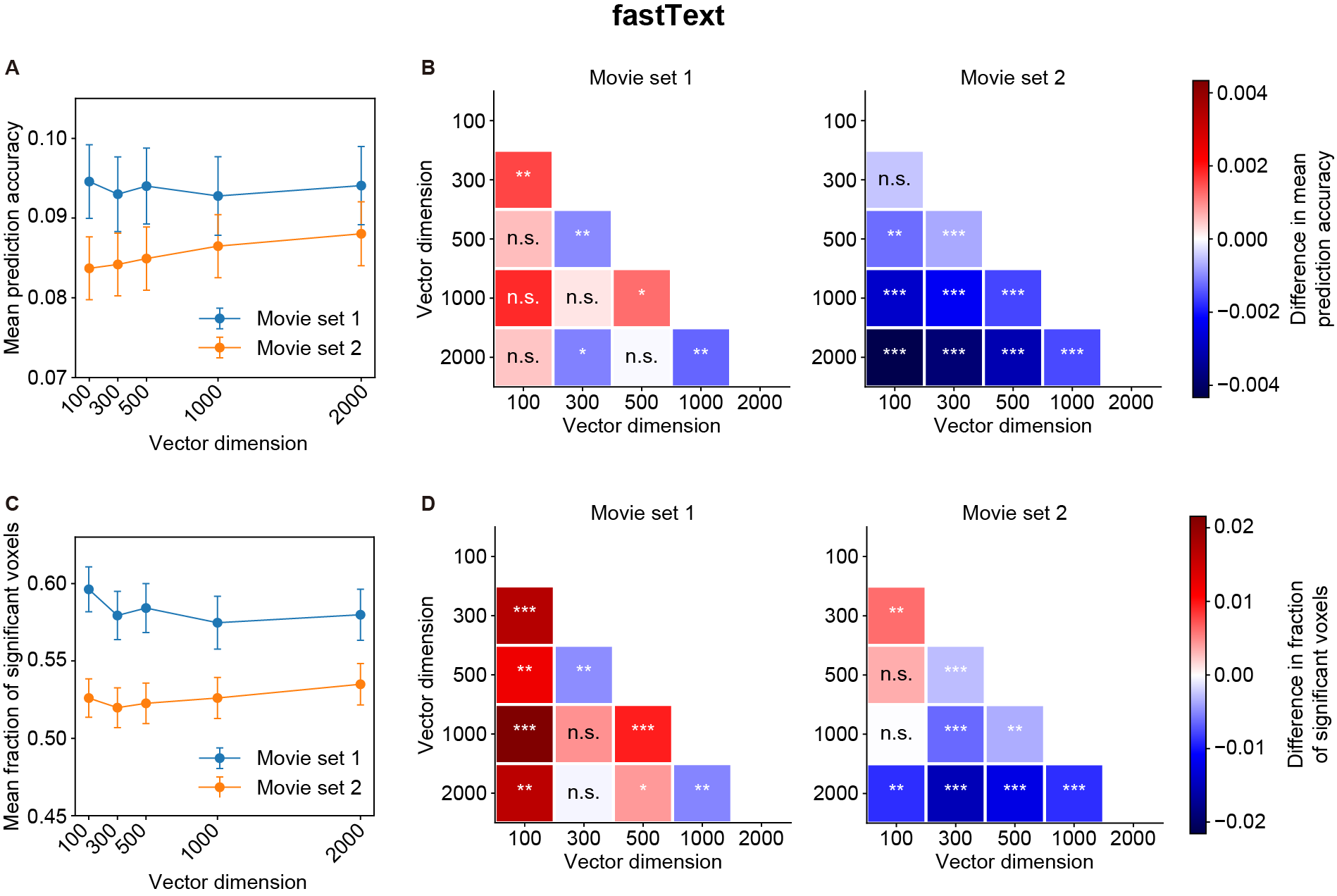

Supplement: S1 Fig — A) Mean prediction accuracy of voxelwise models based on the fastText vector space with different vector dimensions. Error bars indicate standard error of the mean (SEM). B) Difference of mean prediction accuracies between different vector dimensions. The difference was evaluated separately for movie sets 1 (left) and 2 (right). The color of each cell represents the accuracy difference of the dimension on the x-axis minus the dimension on y-axis (red, positive values; blue, negative values). The mark in each cell indicates the statistical significance of the difference (Wilcoxon test, ***p < 0.0001, **p < 0.01, *p < 0.05, n.s., p > 0.05, FDR corrected). C) Fractions of significant voxels for voxelwise models with different vector dimensions. The same conventions were used as in A. D) Difference in fractions of significant voxels between different vector dimensions. The same conventions were used as in B. (TIF) [file pcbi.1009138.s001.tif]

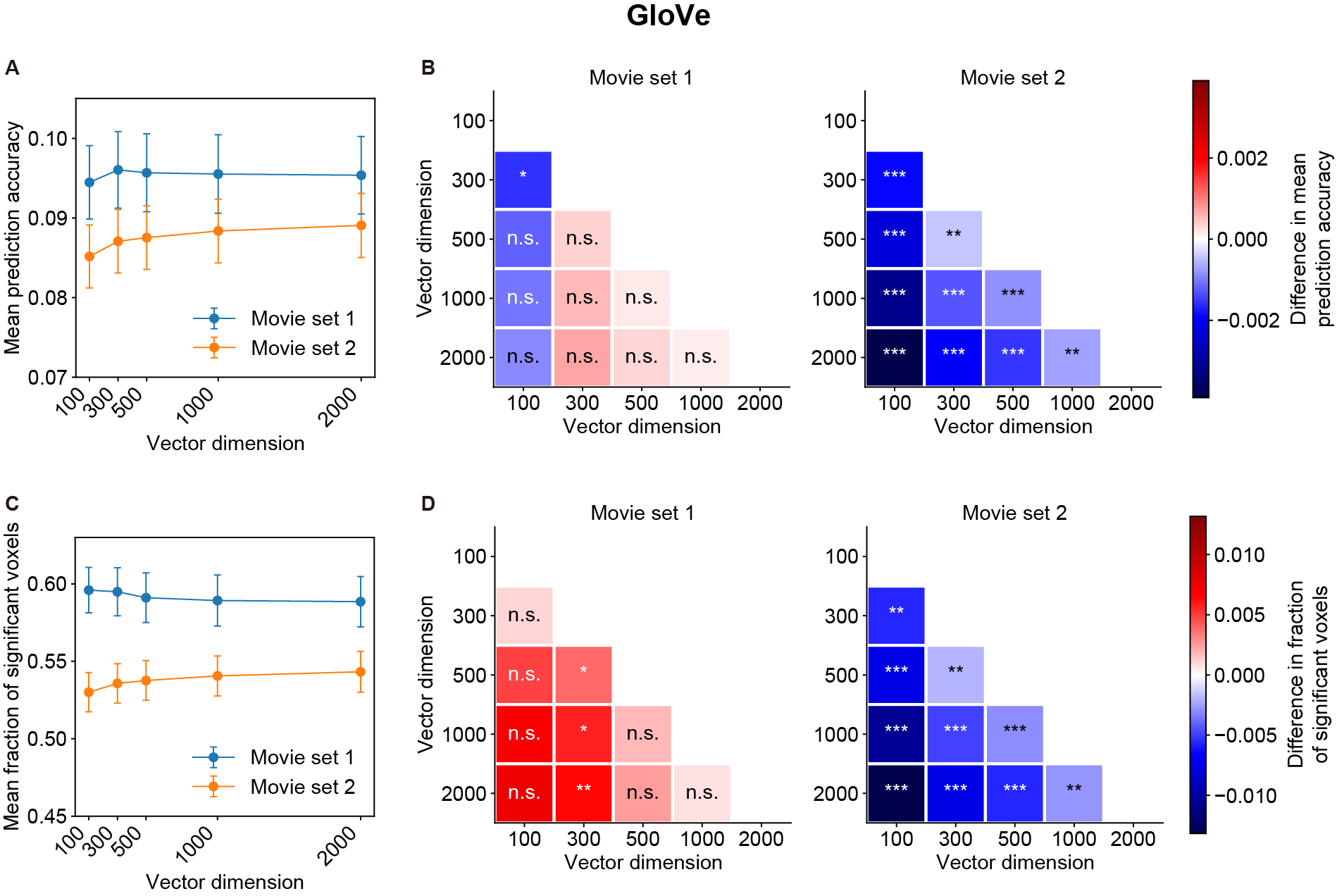

Supplement: S2 Fig — The same analysis as in S1 Fig but for GloVe vectors. (TIF) [file pcbi.1009138.s002.tif]

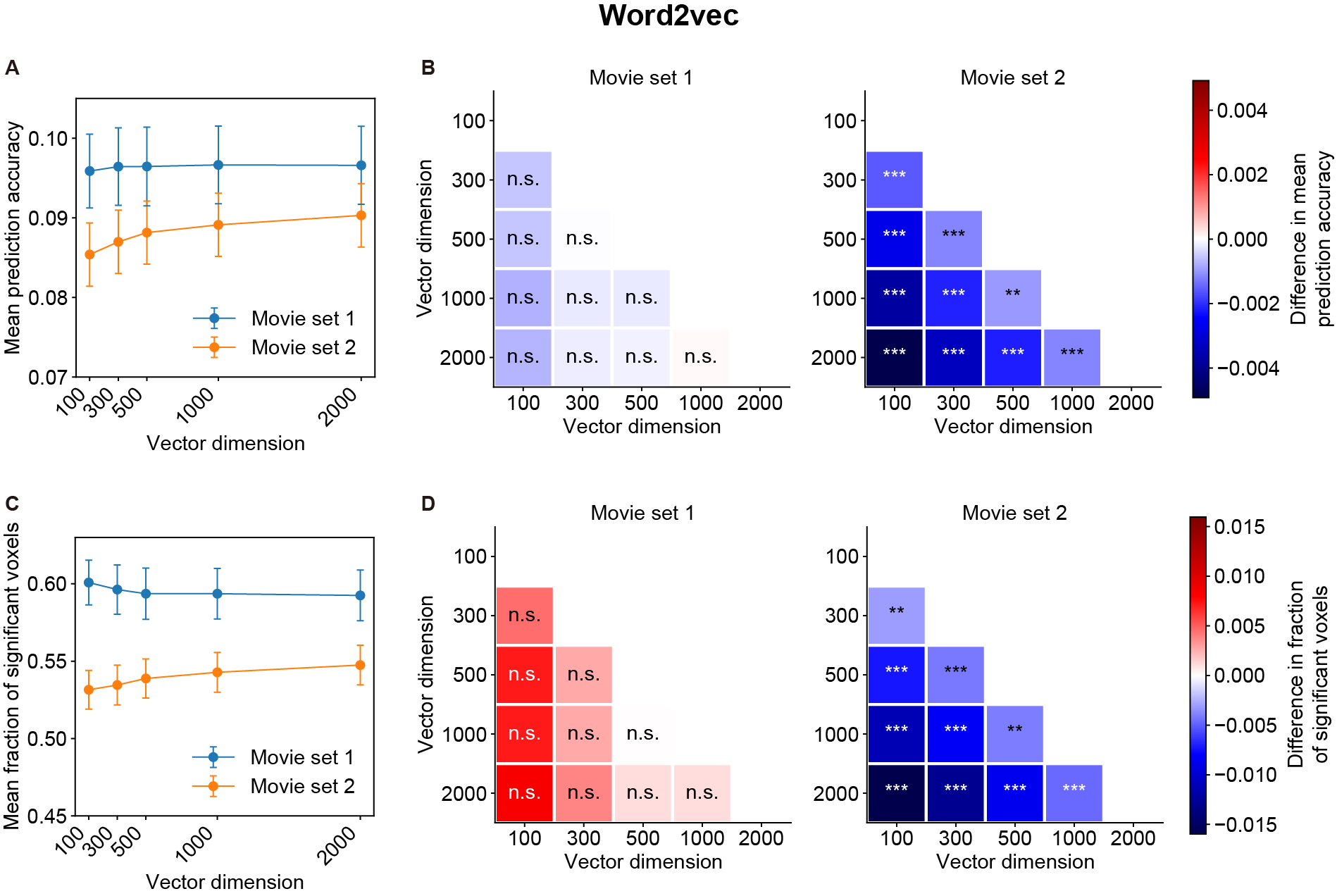

Supplement: S3 Fig — The same analysis as in S1 Fig but for word2vec vectors. (TIF) [file pcbi.1009138.s003.tif]

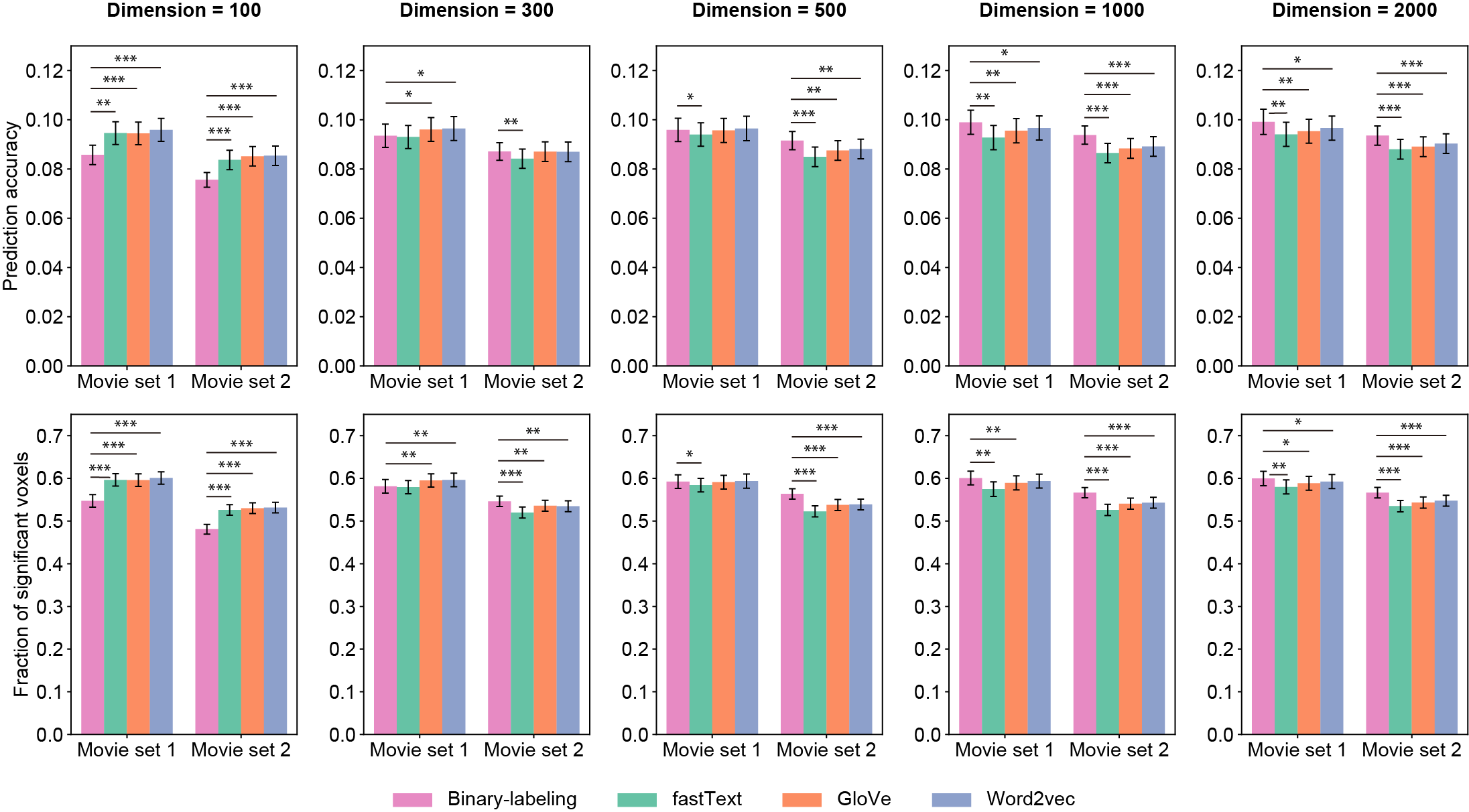

Supplement: S4 Fig — To compare discrete word features with word vectors in terms of the modeling of brain semantic representations, voxelwise models with the binary labeling of movie scenes (binary-labeling models) were constructed for individual participants. Prediction accuracy (top) and the fraction of significant voxels (bottom) for the binary-labeling models (pink bars) are shown separately for the vector dimensionality of 100, 300, 500,1000, and 2000 (from left to right), along with prediction performance for the word vector-based models (green bars, fastText; orange bars, GloVe; blue bars, word2vec). Error bars indicate SEM. Marks above bars indicate the statistical significance of the performance difference between the binary-labeling model and each of the word vector-based models (Wilcoxon test, ***p < 0.0001, **p < 0.01, *p < 0.05, FDR corrected). (TIF) [file pcbi.1009138.s004.tif]

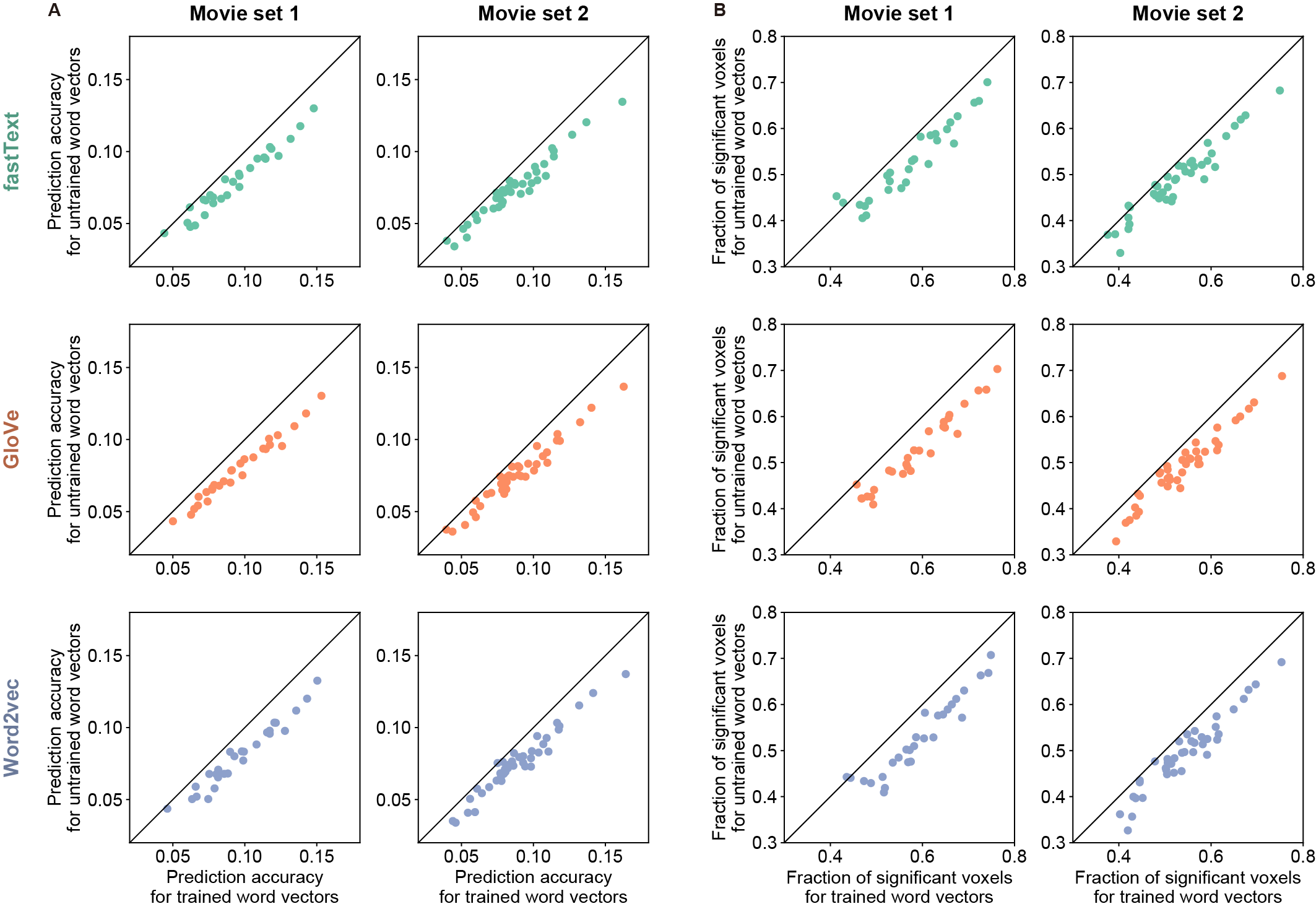

Supplement: S5 Fig — To test whether the semantic relational structure of words, captured by word vectors, is effective in the modeling of cortical semantic representations, we compared trained (original) and untrained word vectors in terms of the model performance of brain-response prediction. We obtained untrained vectors by randomly assigning a 1000-dimensional random vector to each word in the same vocabulary as used for trained vectors. Hence, untrained vectors had signatures of individual words but not the semantic relational structure of words. Voxelwise models based on untrained vectors were constructed using the same procedure as used for voxelwise models based on trained vectors. A) Prediction accuracy for models based on trained vectors (x-axis) and ones based on untrained vectors (y-axis) separately shown for each word-vector type (top, fastText; middle, GloVe; bottom, word2vec) and each dataset (left, movie set 1; right, movie set 2). Each dot represents mean prediction accuracy over all voxels of each brain. The models based on trained vectors exhibited significantly higher prediction accuracy than those based on untrained vectors regardless of vector types and datasets (Wilcoxon test, p < 0.00001, FDR corrected). B) The fraction of significant voxels for models based on trained vectors (x-axis) and ones based on untrained vectors (y-axis). Each dot represents the fraction for each brain. The models based on trained vectors exhibited significantly higher fraction than those based on untrained vectors regardless of vector types and datasets (p < 0.00001, FDR corrected). (TIF) [file pcbi.1009138.s005.tif]

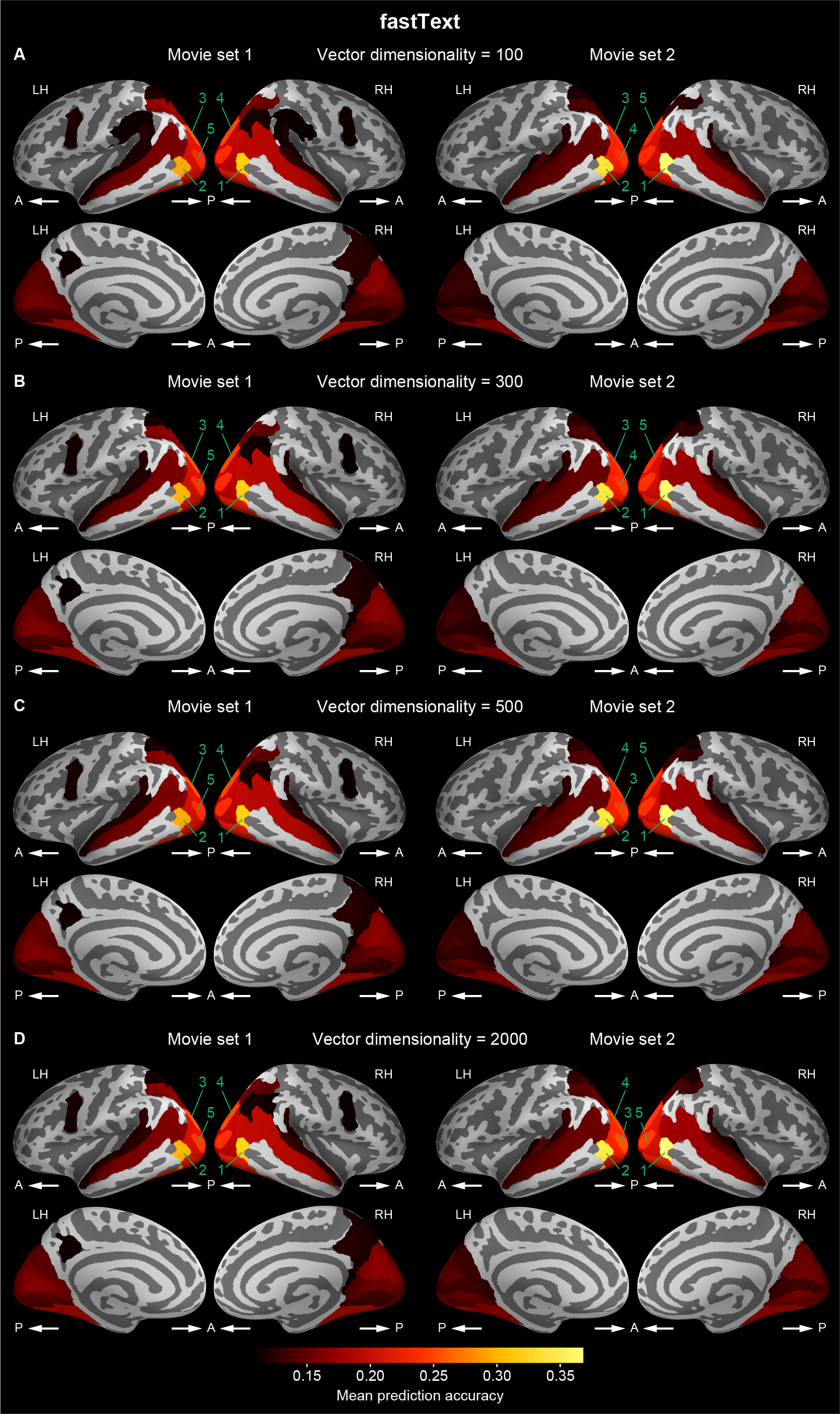

Supplement: S6 Fig — The mean prediction accuracy of fastText vector-based voxelwise models in each brain region was mapped onto the cortical surface (A, vector dimension = 100; B, 300; C, 500; D, 2000). The same conventions were used as in Fig 4. The five numbered cortical regions with the highest mean prediction accuracy for each dimension and dataset are shown in S1 Table. (TIF) [file pcbi.1009138.s006.tif]

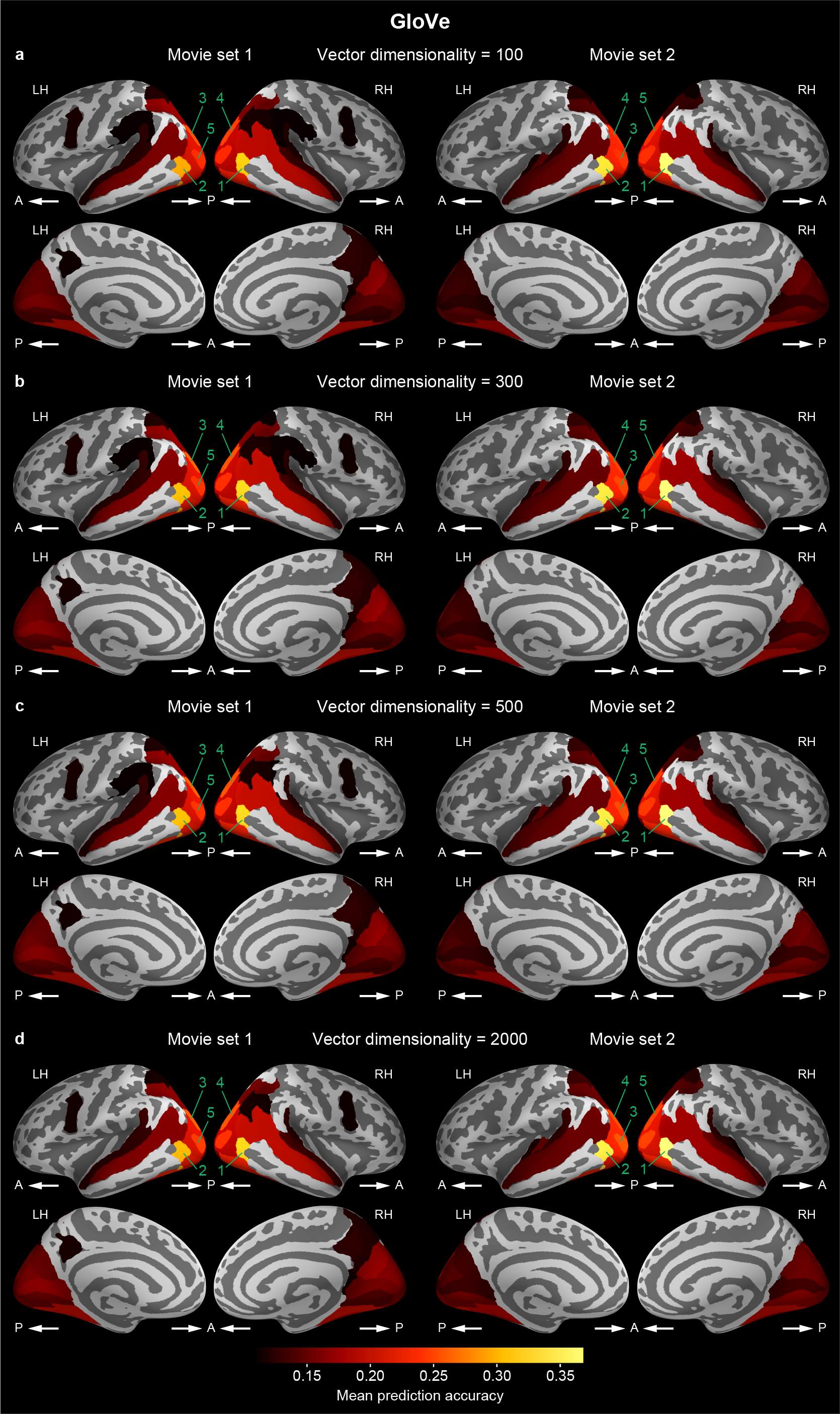

Supplement: S7 Fig — The same analysis as in S6 Fig but for GloVe vector-based models. The numbered cortical regions are shown in S2 Table. (TIF) [file pcbi.1009138.s007.tif]

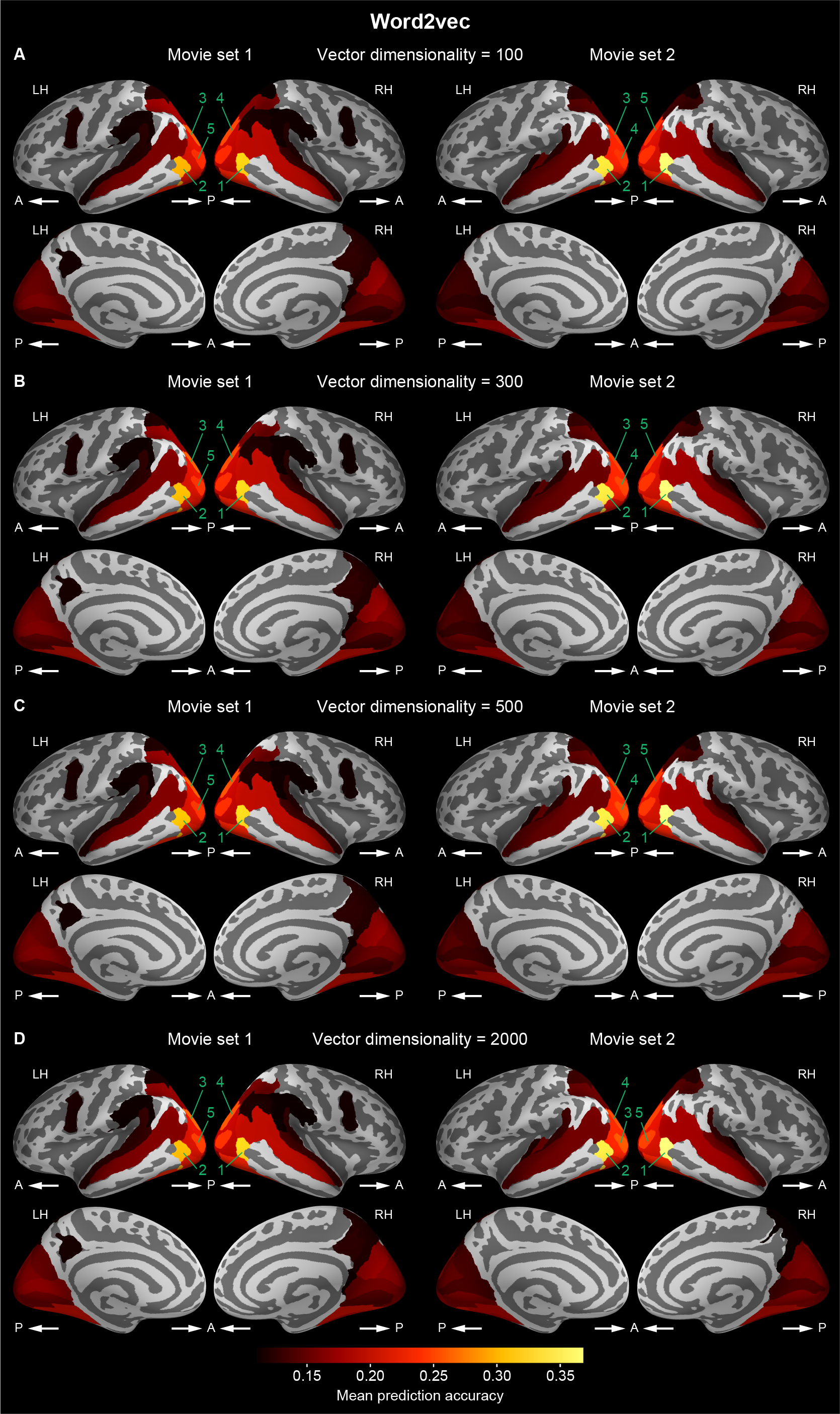

Supplement: S8 Fig — The same analysis as in S6 Fig but for word2vec vector-based models. The numbered cortical regions are shown in S3 Table. (TIF) [file pcbi.1009138.s008.tif]

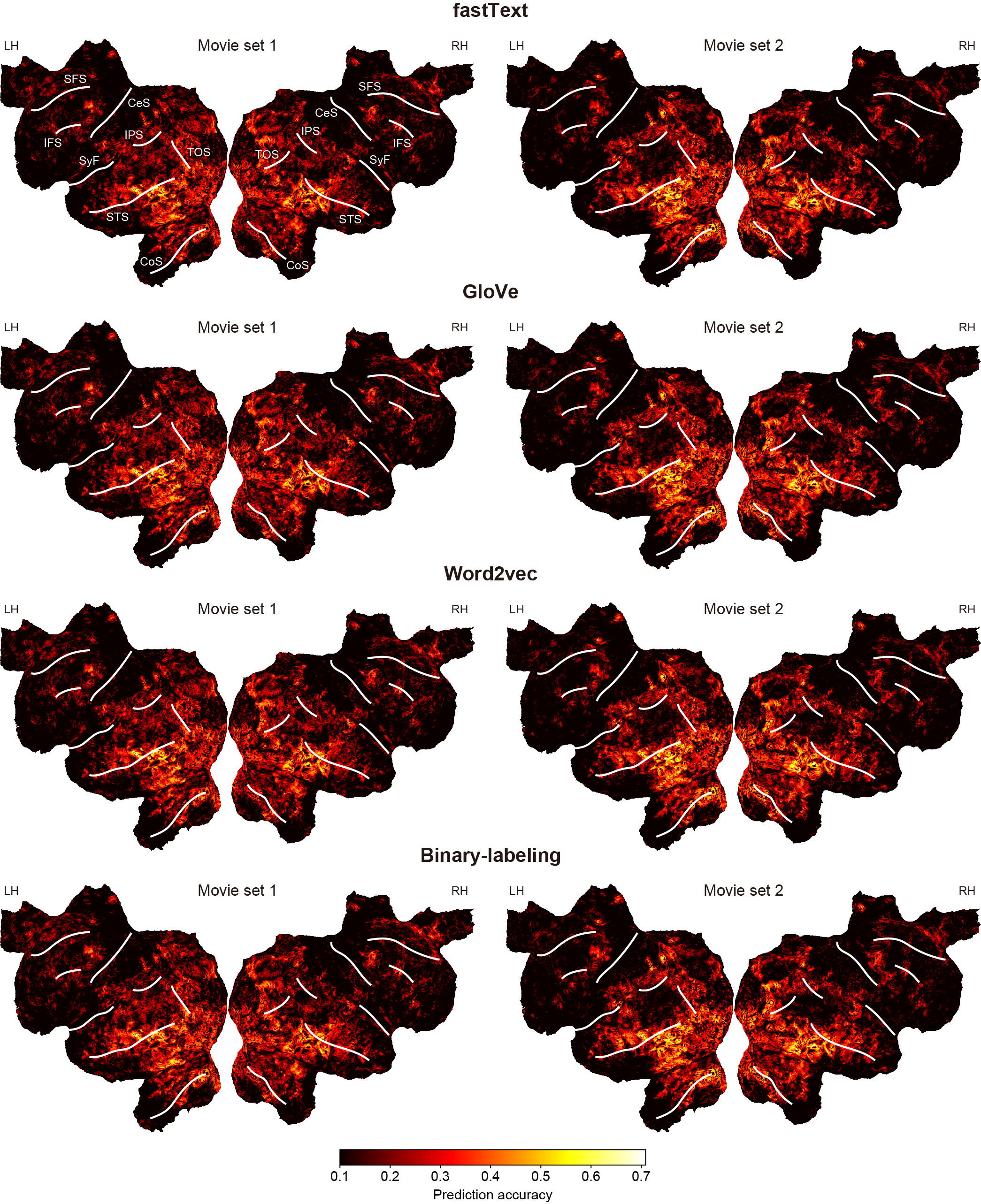

Supplement: S9 Fig — Voxelwise prediction accuracy of each word vector-based voxelwise model (vector dimension = 1000) for a representative participant was mapped onto the cortical flat map of the participant (from top to bottom: fastText, GloVe, word2vec, and binary labeling models; left: movie set 1, right: movie set 2). Brighter colors on the cortical maps indicate voxels with higher prediction accuracy. Only voxels with prediction accuracy above 0.10 are shown. The values of voxelwise prediction accuracy range up to 0.709. White lines on the cortical maps denote representative sulci: CoS, collateral sulcus; STS, superior temporal sulcus; TOS, transverse occipital sulcus; IPS, intraparietal sulcus; SyF, sylvian fissure; CeS, central sulcus; SFS, superior frontal sulcus; IFS, inferior frontal sulcus. (TIF) [file pcbi.1009138.s009.tif]

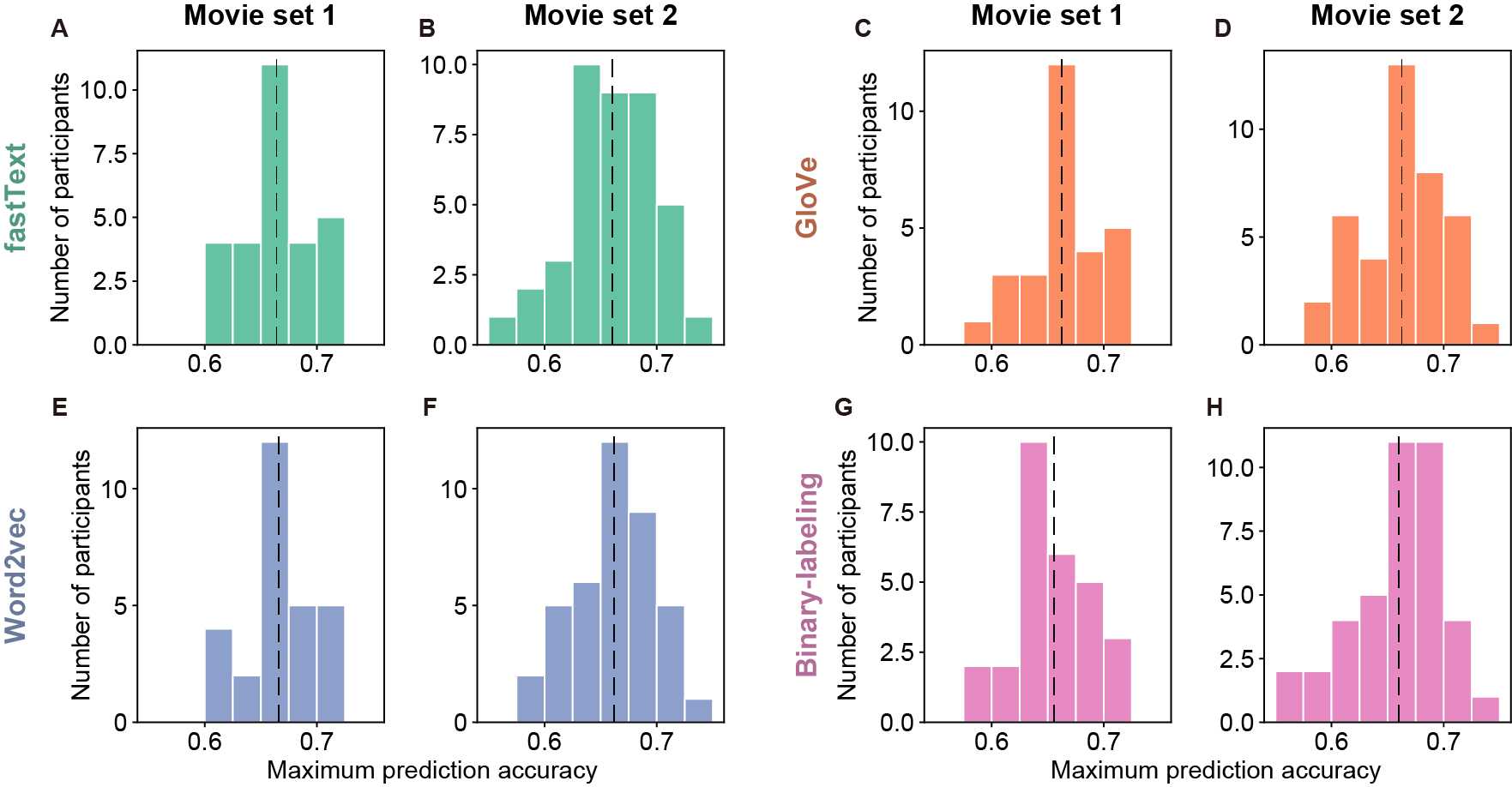

Supplement: S10 Fig — The distribution of the maximum values of voxelwise prediction accuracy for individual participants was shown separately for each model (A and B: fastText; C and D: GloVe; E and F: word2vec; G and H: binary-labeling) and each dataset (A, C, E, and G: movie set 1; B, D, F, and H: movie set 2). The vertical dashed line in each panel indicates the mean value of the maximum prediction accuracy averaged across participants. (TIF) [file pcbi.1009138.s010.tif]

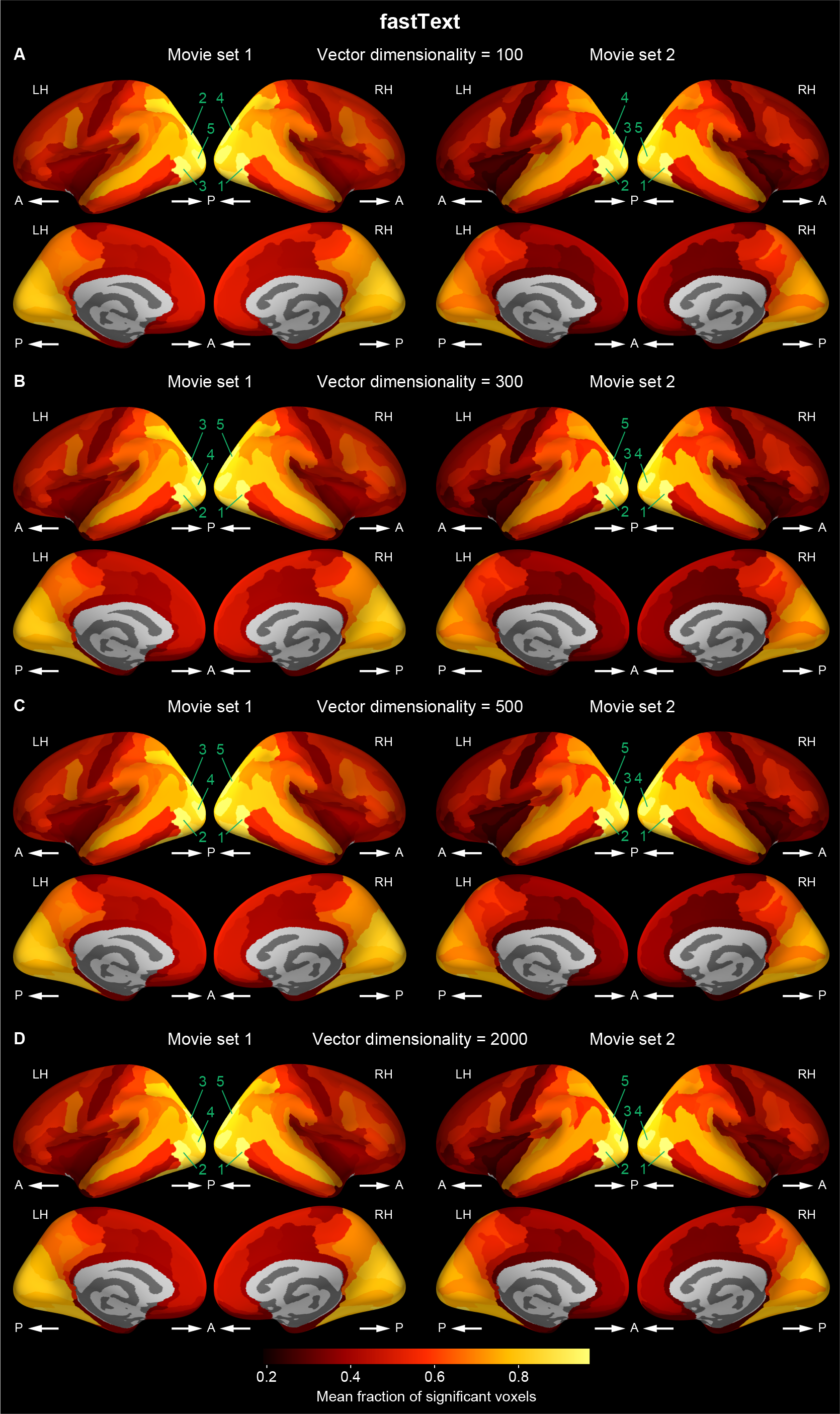

Supplement: S11 Fig — The mean fraction of significant voxels in each brain region for fastText vector-based models was mapped onto the cortical surface (A, vector dimension = 100; B, 300; C, 500; D, 2000). The same conventions were used as in Fig 5. The five numbered cortical regions with the highest mean fraction of significant voxels for each dimension and dataset are shown in S7 Table. (TIF) [file pcbi.1009138.s011.tif]

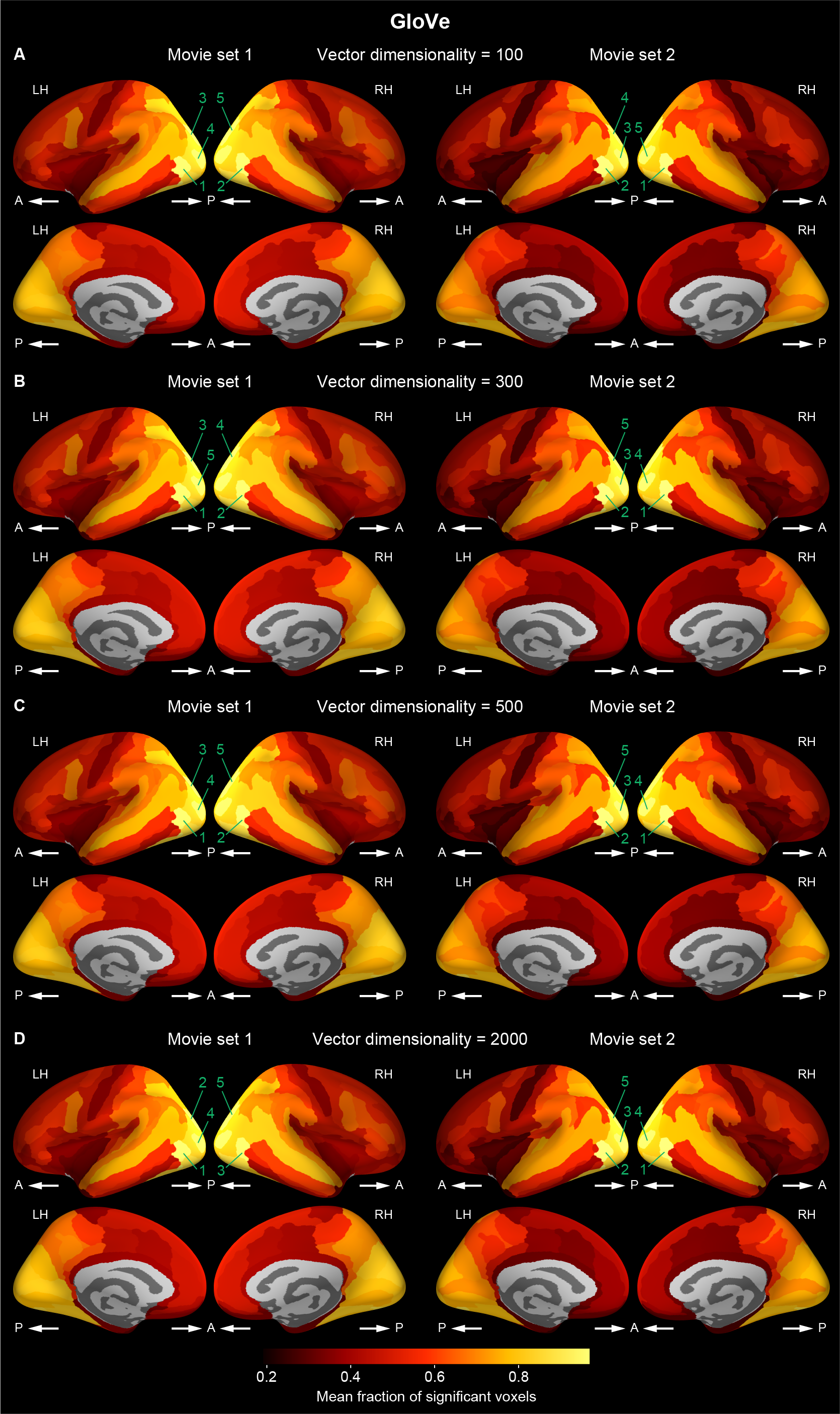

Supplement: S12 Fig — The same analysis as in S11 Fig but for GloVe vector-based models. The numbered cortical regions are shown in S8 Table. (TIF) [file pcbi.1009138.s012.tif]

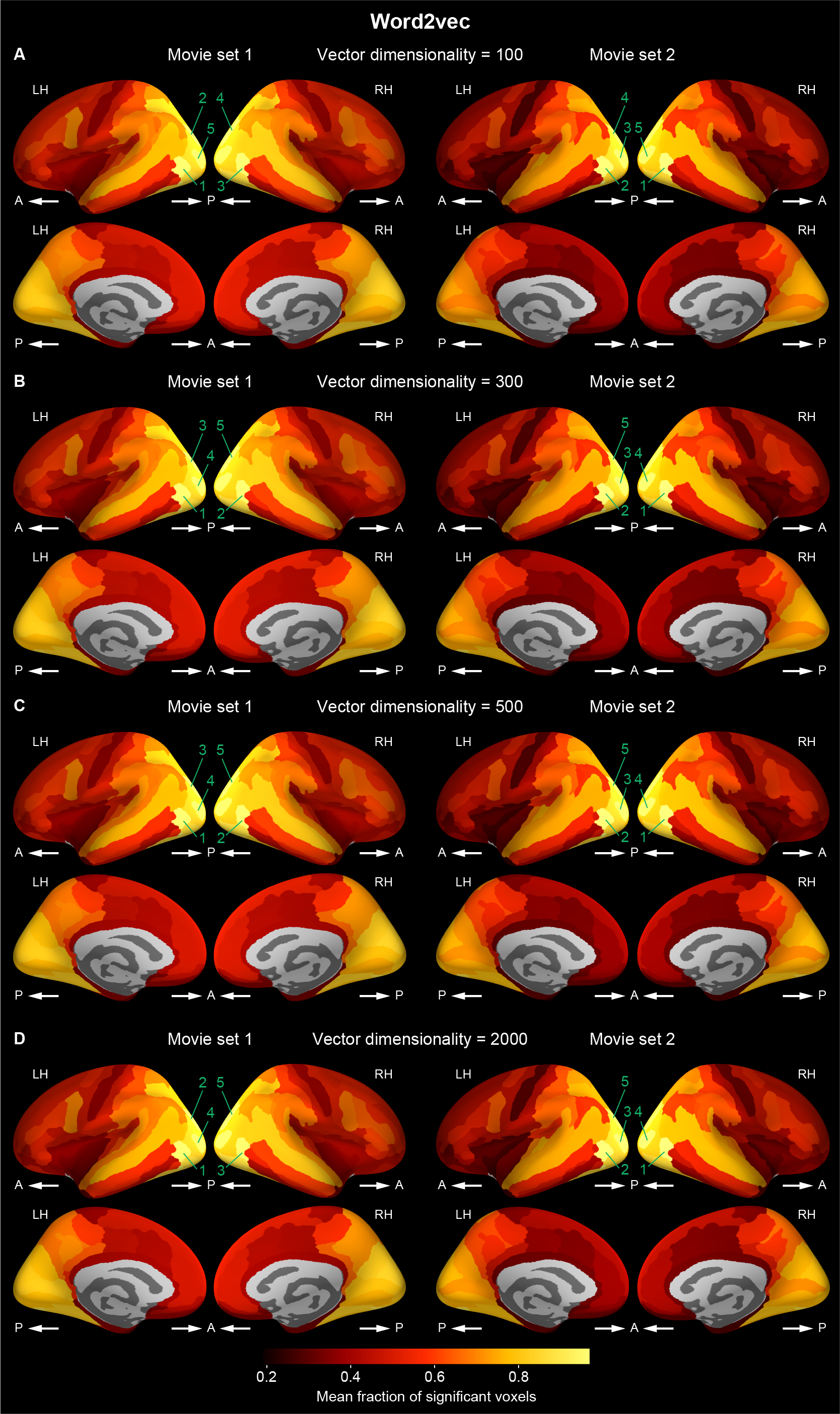

Supplement: S13 Fig — The same analysis as in S11 Fig but for word2vec vector-based models. The numbered cortical regions are shown in S9 Table. (TIF) [file pcbi.1009138.s013.tif]

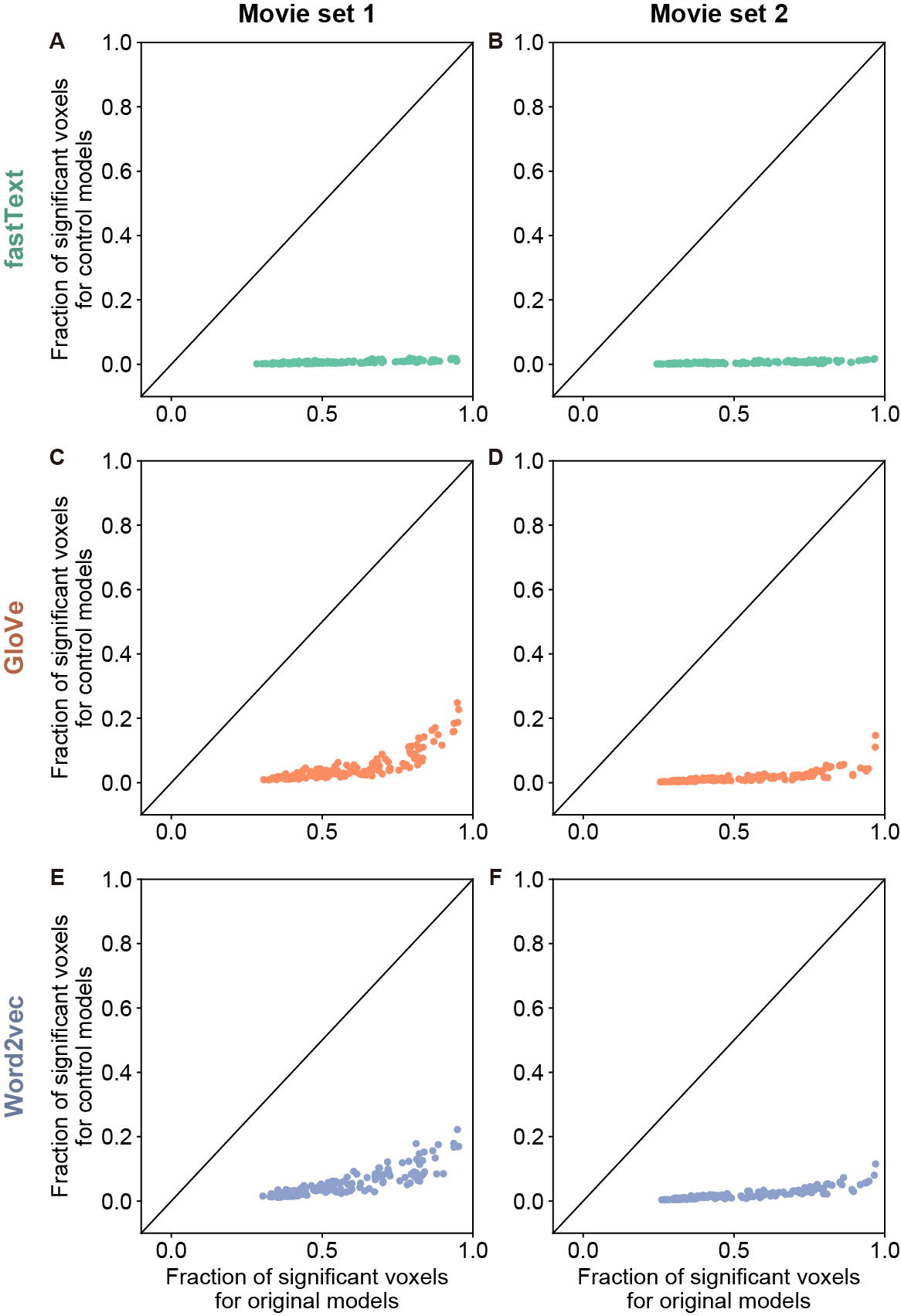

Supplement: S14 Fig — To test the significance of the fraction of significant voxels for original models, we compared the original fraction with the fraction of significant voxels for control models. The control models used word vectors randomly shuffled across vector dimensions for each vector and thereby produced the chance level of the fraction of significant voxels. The fraction of significant voxels in each cortical region for these original (y-axis) and control (x-axis) models is shown separately for each word-vector type (A and B: fastText; C and D: GloVe; E and F: word2vec) and each dataset (A, C, and E: movie set 1; B, D, and F: movie set 2). Each dot represents the fraction in each cortical region. Regardless of the word-vector types and datasets, the fraction for the original models was significantly higher than that for the control models in all the cortical regions (Wilcoxon test, p < 0.00001, FDR corrected). This result indicates that the fraction of significant voxels for the original models is sufficiently above chance level. (TIF) [file pcbi.1009138.s014.tif]

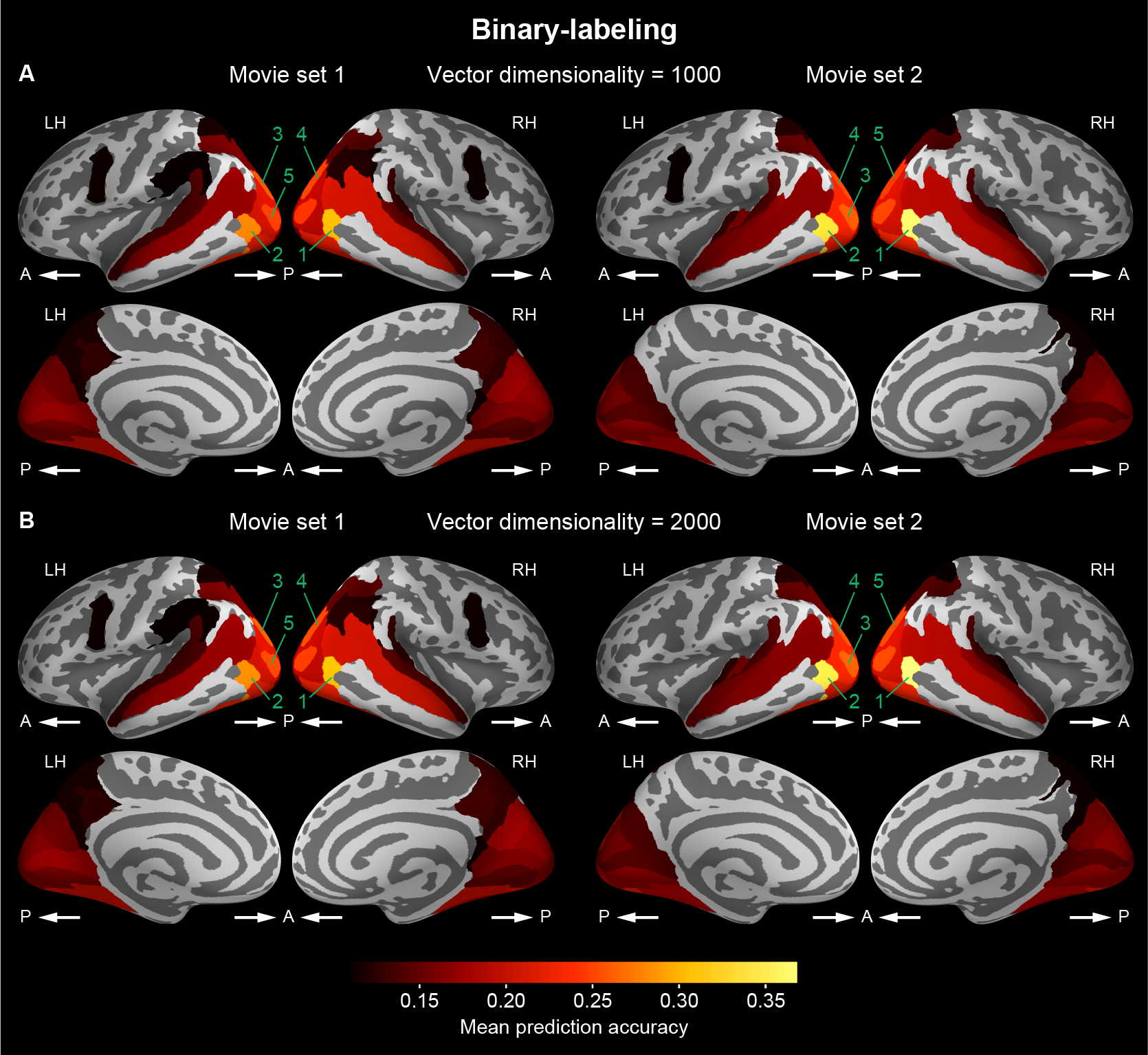

Supplement: S15 Fig — The same analysis as in S6 Fig but for binary-labeling models with the vector dimensionality of 1000 and 2000. The numbered cortical regions are shown in S13 Table. (TIF) [file pcbi.1009138.s015.tif]

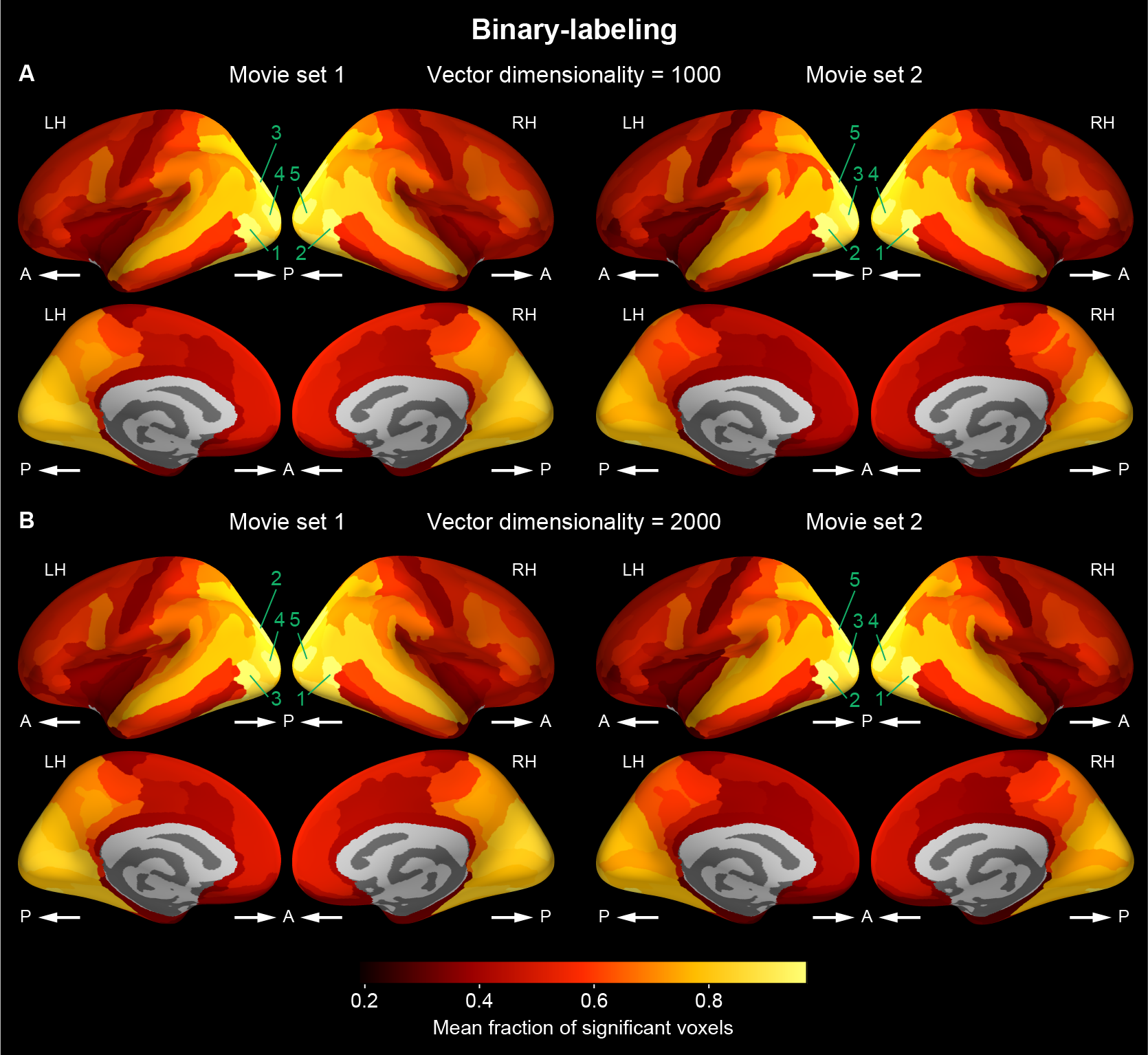

Supplement: S16 Fig — The same analysis as in S11 Fig but for binary-labeling models with the vector dimensionality of 1000 and 2000. The numbered cortical regions are shown in S14 Table. (TIF) [file pcbi.1009138.s016.tif]

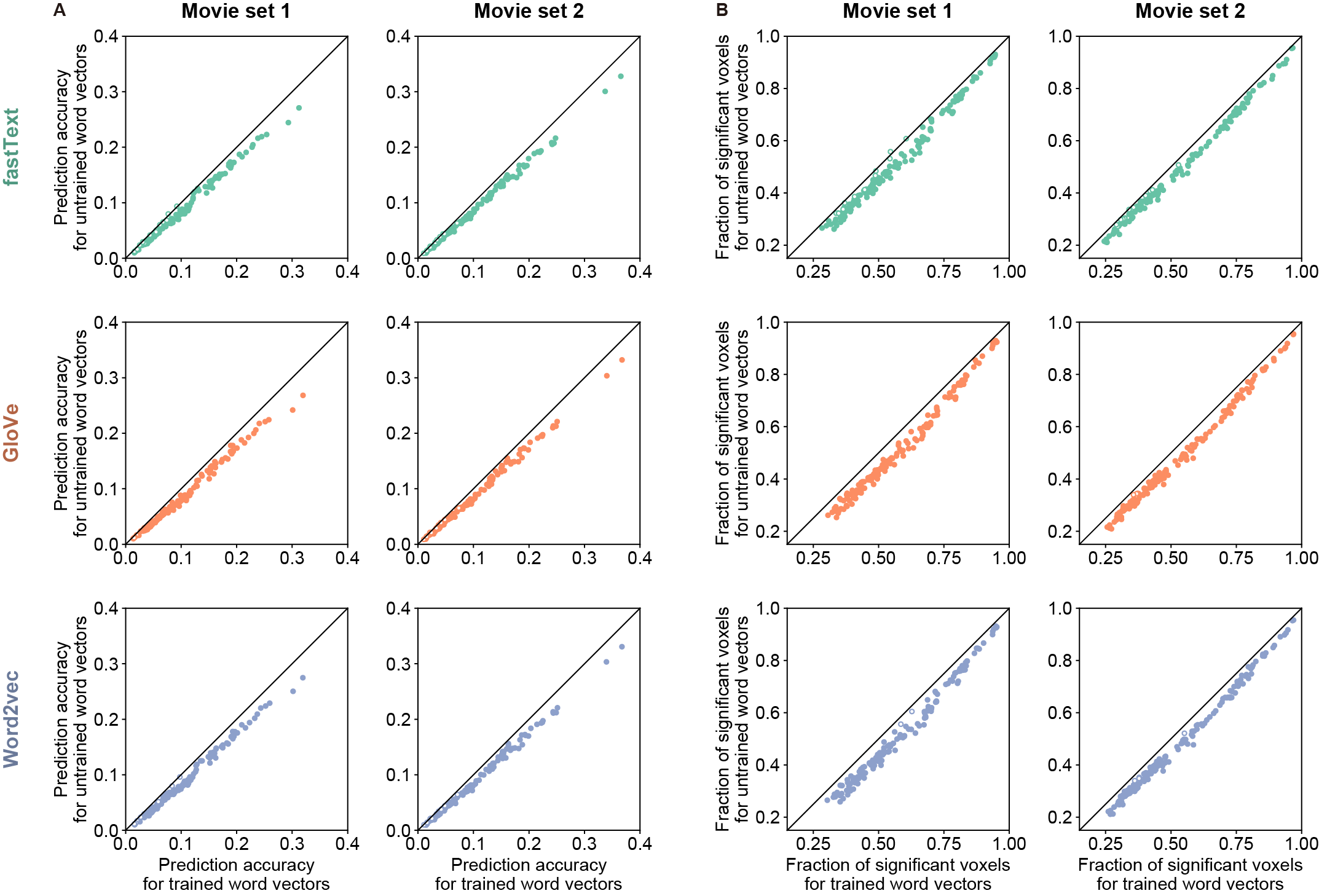

Supplement: S17 Fig — Prediction performance of voxelwise models (A, prediction accuracy; B, the fraction of significant voxels) in each cortical region was compared between trained (x-axis) and untrained word vectors (y-axis). The performance is shown separately for each word-vector type (top, fastText; middle, GloVe; bottom, word2vec) and each dataset (left in each of A and B, movie set 1; right, movie set 2). Each dot represents the mean performance averaged over participants for each region. The difference of model prediction performance between trained and untrained vectors was tested within each region while the performance for each participant was used as a data sample. Filled and open dots indicate that the region-wise difference was significant or not, respectively (Wilcoxon test, p < 0.05, FDR corrected). Then, the difference of model prediction performance between trained and untrained vectors was tested across regions while the mean performance in each region was used as a data sample. The difference across regions were significant regardless of prediction performance measures, word-vector types, and datasets (Wilcoxon test, p < 0.00001, FDR corrected). (TIF) [file pcbi.1009138.s017.tif]

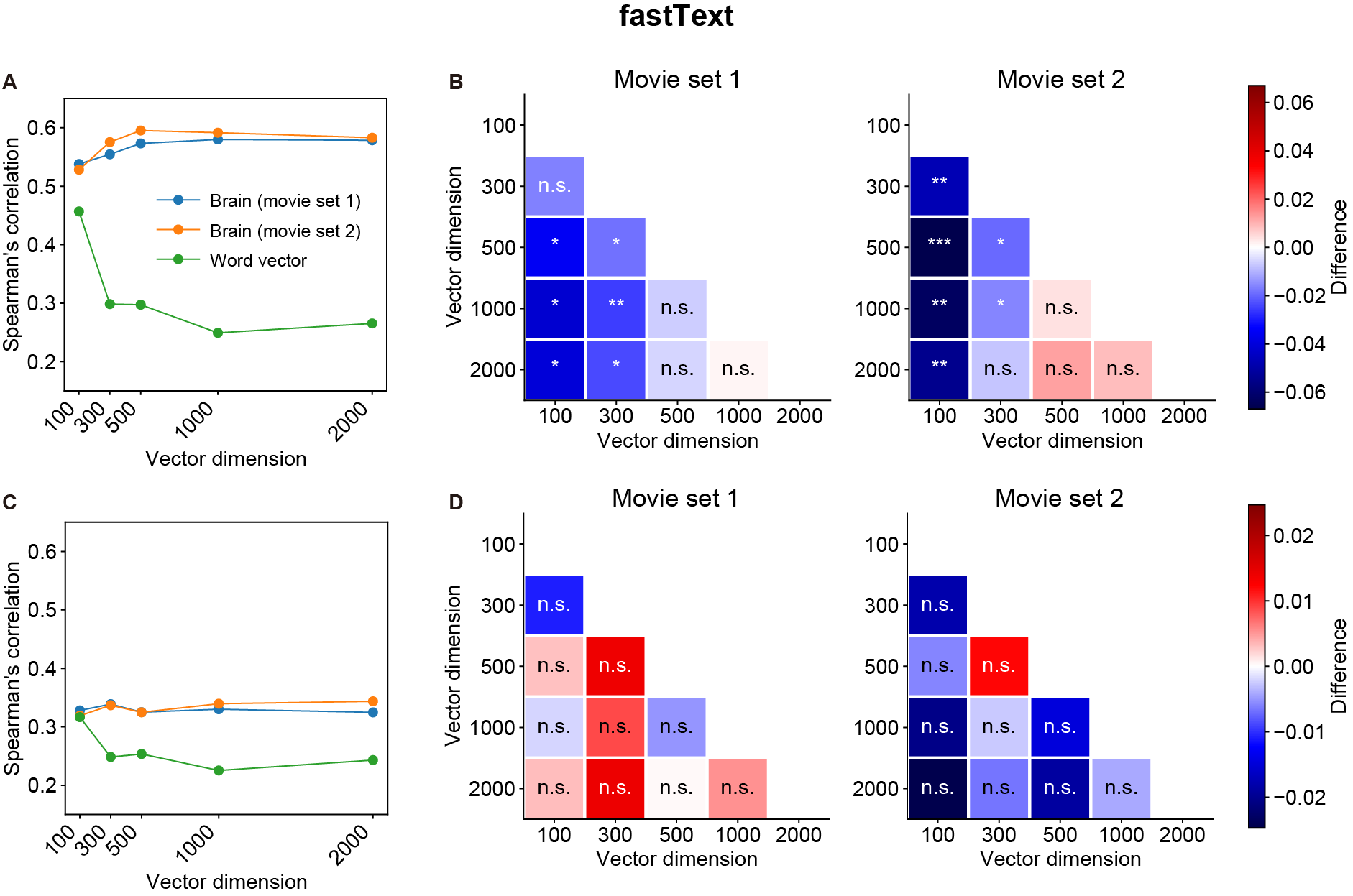

Supplement: S18 Fig — A) Brain–behavior and word vector–behavior correlations of noun dissimilarity matrices for different dimensions of fastText vectors. B) Difference between brain–behavior correlation coefficients of different vector dimensions for nouns. The color of each cell represents the coefficient difference of the dimension on the x-axis minus the dimension on the y-axis (red, positive values; blue, negative values). The mark in each cell indicates the statistical significance of the difference (permutation test, ***p < 0.0001, **p < 0.01, *p < 0.05, n.s., p > 0.05, FDR corrected). (C and D) The same analyses were performed as in A and B using adjective dissimilarity matrices. (TIF) [file pcbi.1009138.s018.tif]

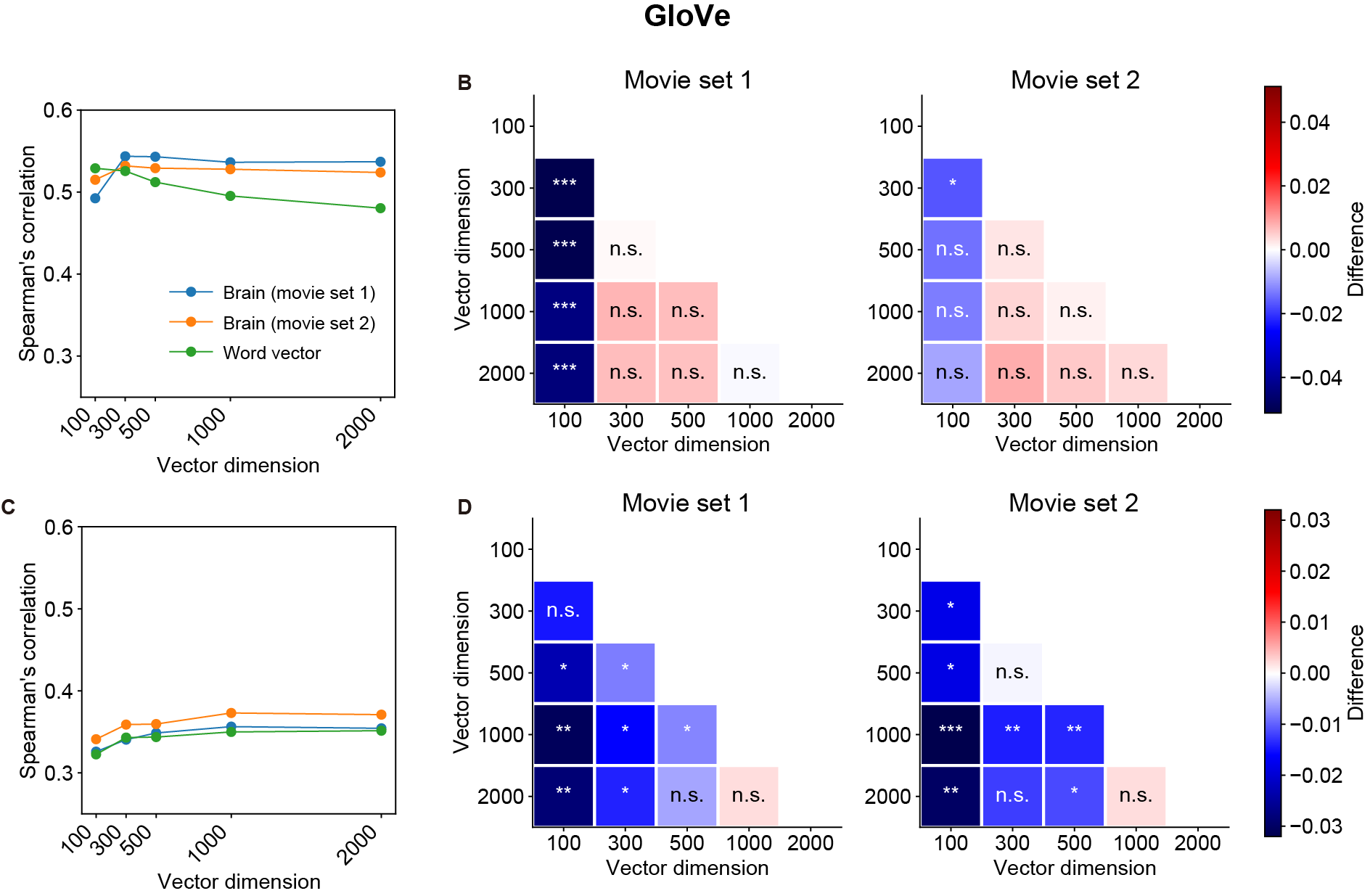

Supplement: S19 Fig — The same analysis as in S18 but for GloVe vectors. (TIF) [file pcbi.1009138.s019.tif]

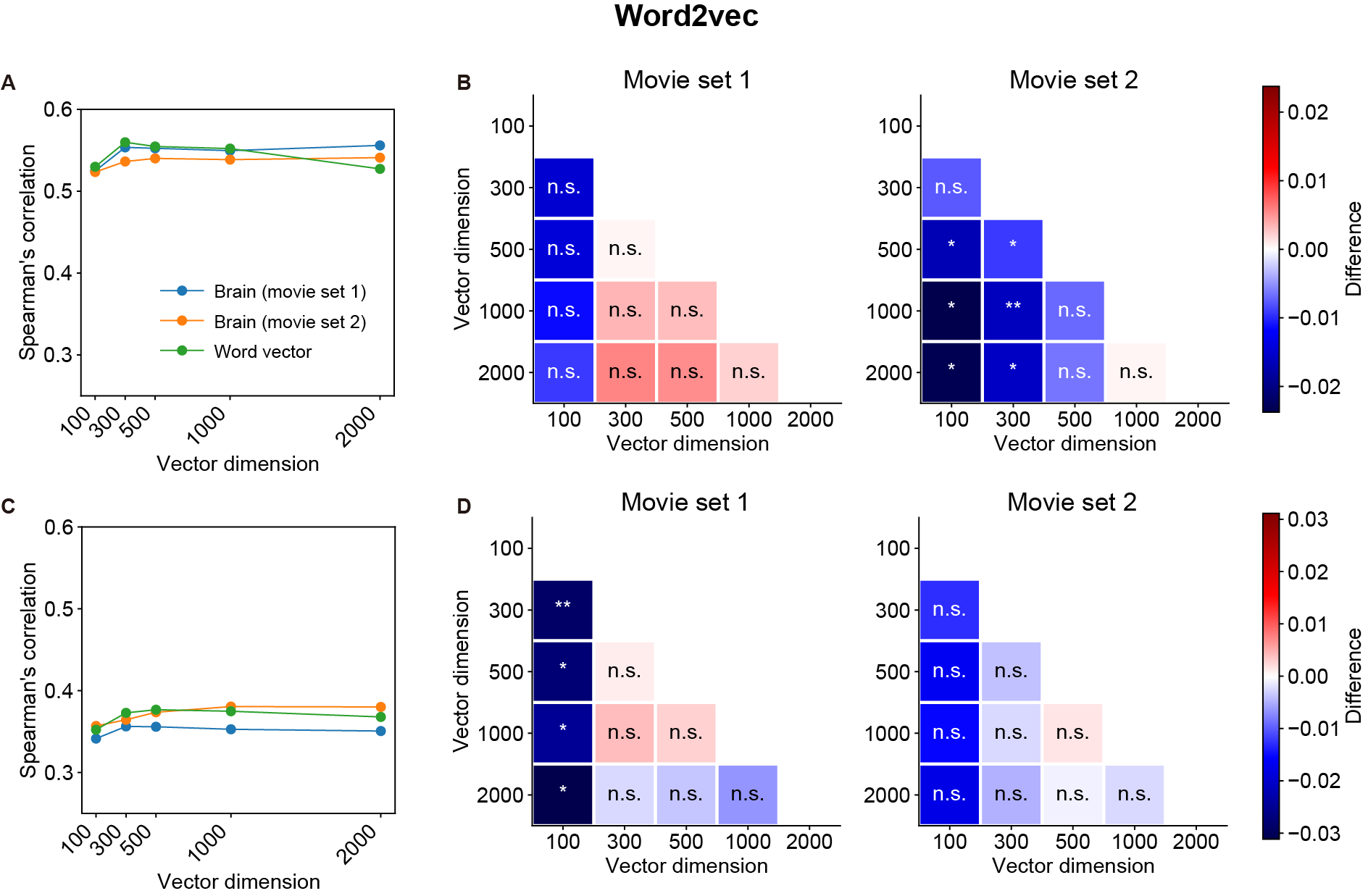

Supplement: S20 Fig — The same analysis as in S18 but for word2vec vectors. (TIF) [file pcbi.1009138.s020.tif]

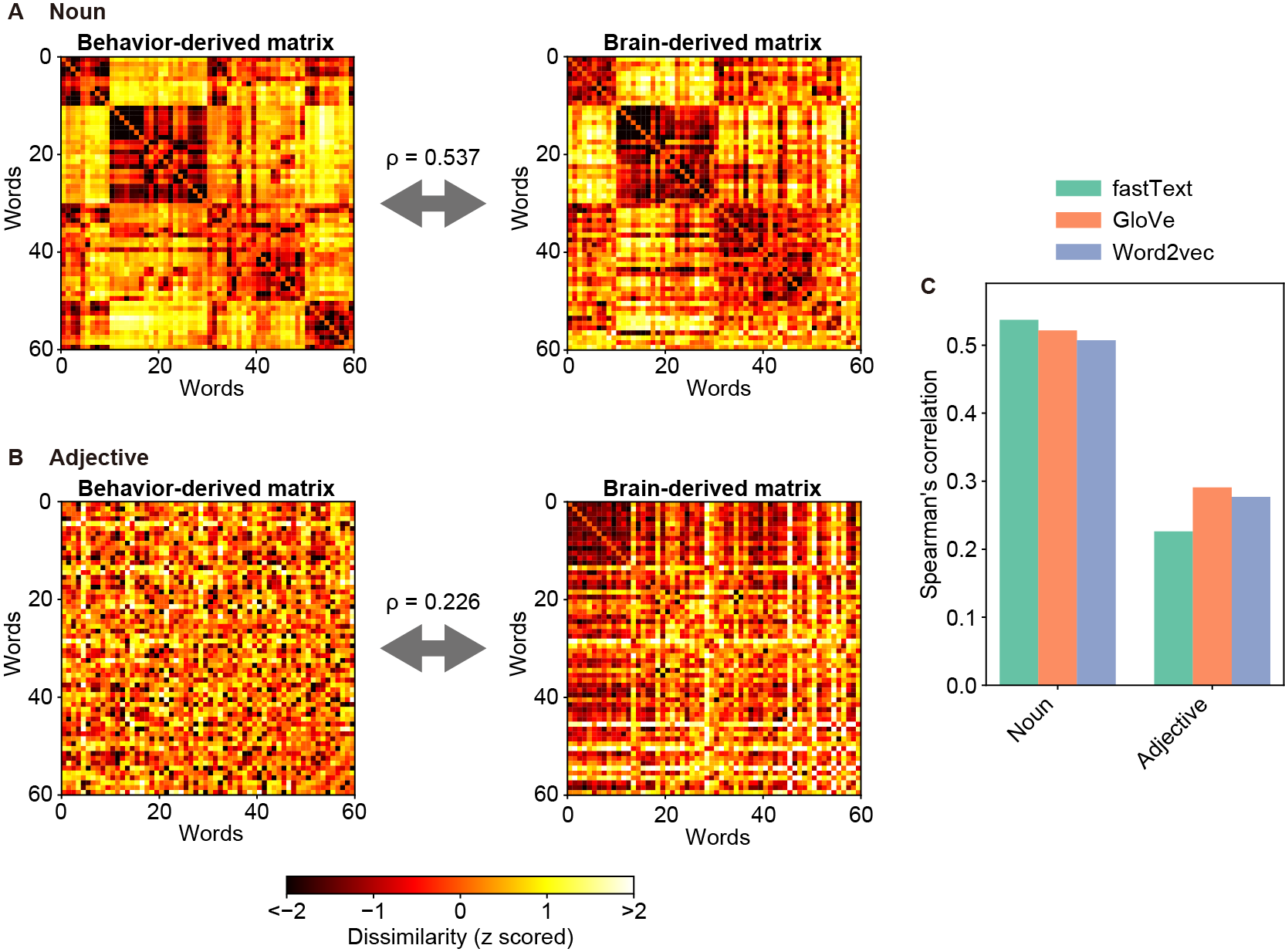

Supplement: S21 Fig — We constructed word dissimilarity matrices using behavioral and voxelwise-model data obtained from the same population of 6 participants for movie set 1. A, B) Behavior- and brain-derived dissimilarity matrices for nouns (A) and adjectives (B). The brain-derived matrices were obtained using 1000-dimensional fastText vectors. The same conventions were used as in Fig 6. C) Brain–behavior correlations for nouns and adjectives. All the correlation coefficients were significantly higher than chance level (permutation test, p < 0.0001, FDR corrected). (TIF) [file pcbi.1009138.s021.tif]

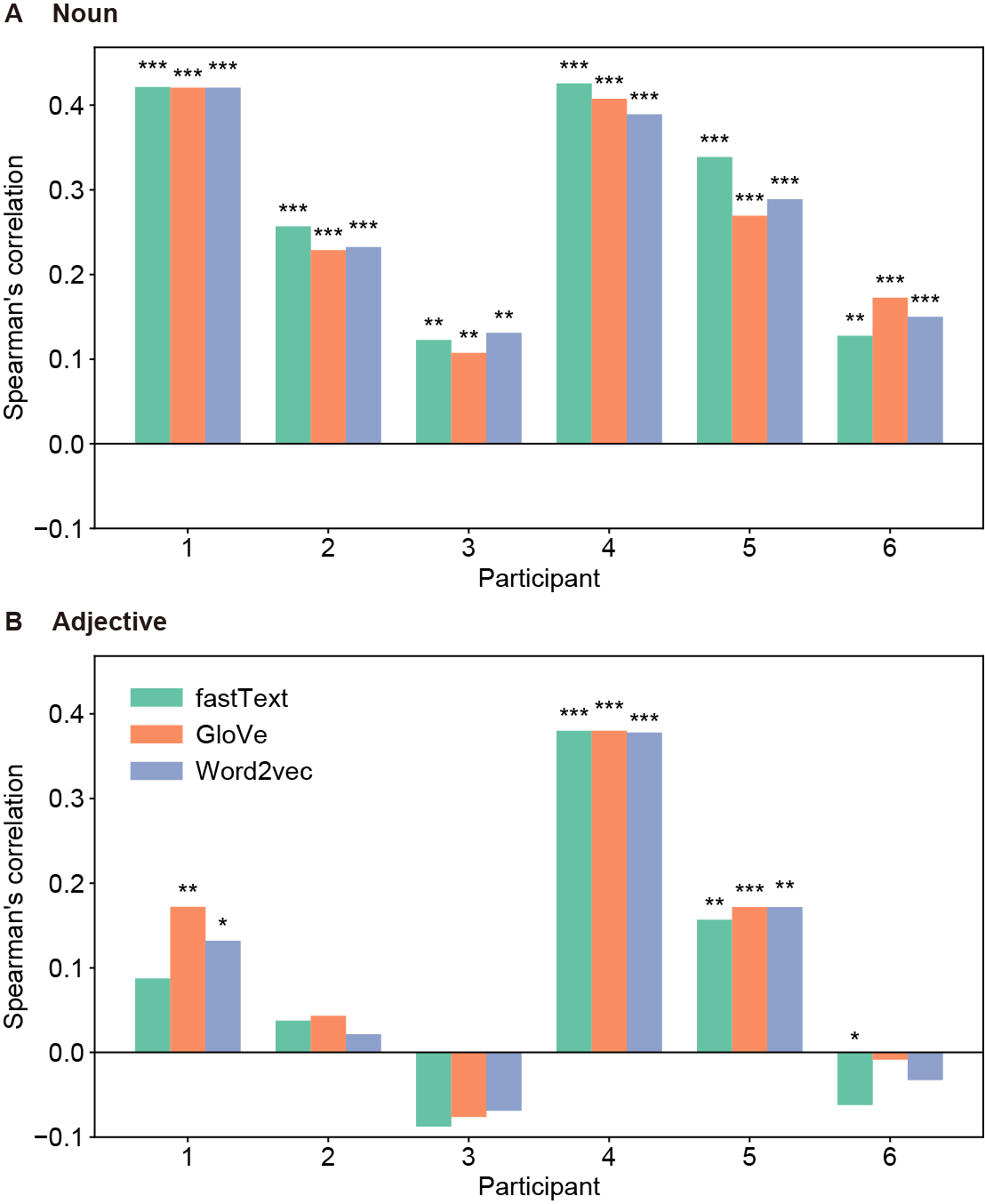

Supplement: S22 Fig — We calculated the Spearman’s correlation between brain- and behavior-derived word dissimilarity matrices for each of the 6 participants who had both brain and behavioral data. The correlation coefficients are shown separately for nouns (A) and adjectives (B) and for each type of word vectors (dimensionality = 1000). Marks above bars indicate the statistical significance of the correlation coefficients (permutation test, ***p < 0.0001, **p < 0.01, *p < 0.05, FDR corrected). (TIF) [file pcbi.1009138.s022.tif]

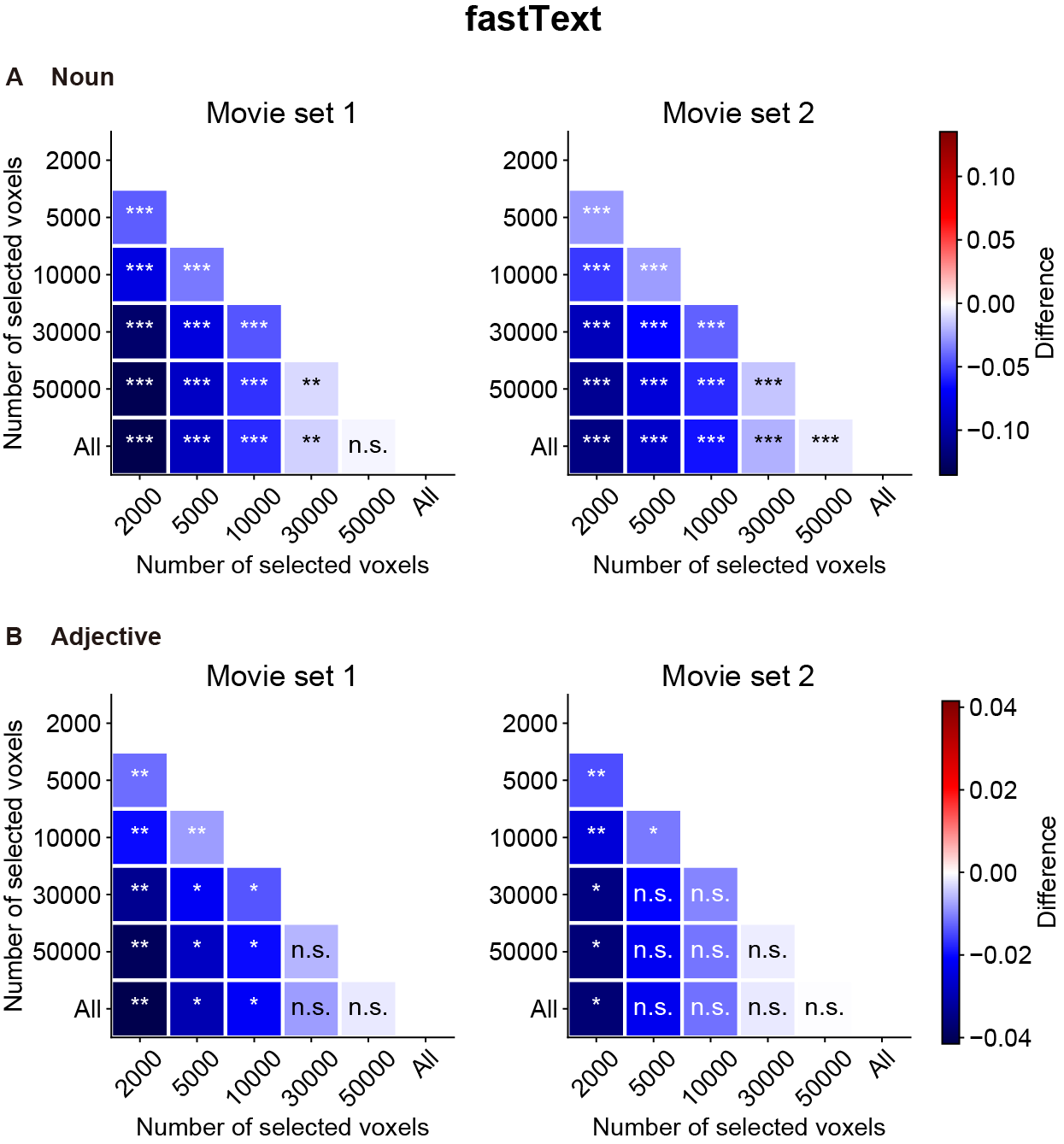

Supplement: S23 Fig — The color of each cell represents the difference between each pair of the number of selected voxels; the difference is the brain–behavior correlation coefficient for the dimension on the x-axis minus the coefficient for the dimension on the y-axis (red, positive values; blue, negative values). The mark in each cell indicates the statistical significance of the difference (permutation test, ***p < 0.0001, **p < 0.01, *p < 0.05, FDR corrected). (TIF) [file pcbi.1009138.s023.tif]

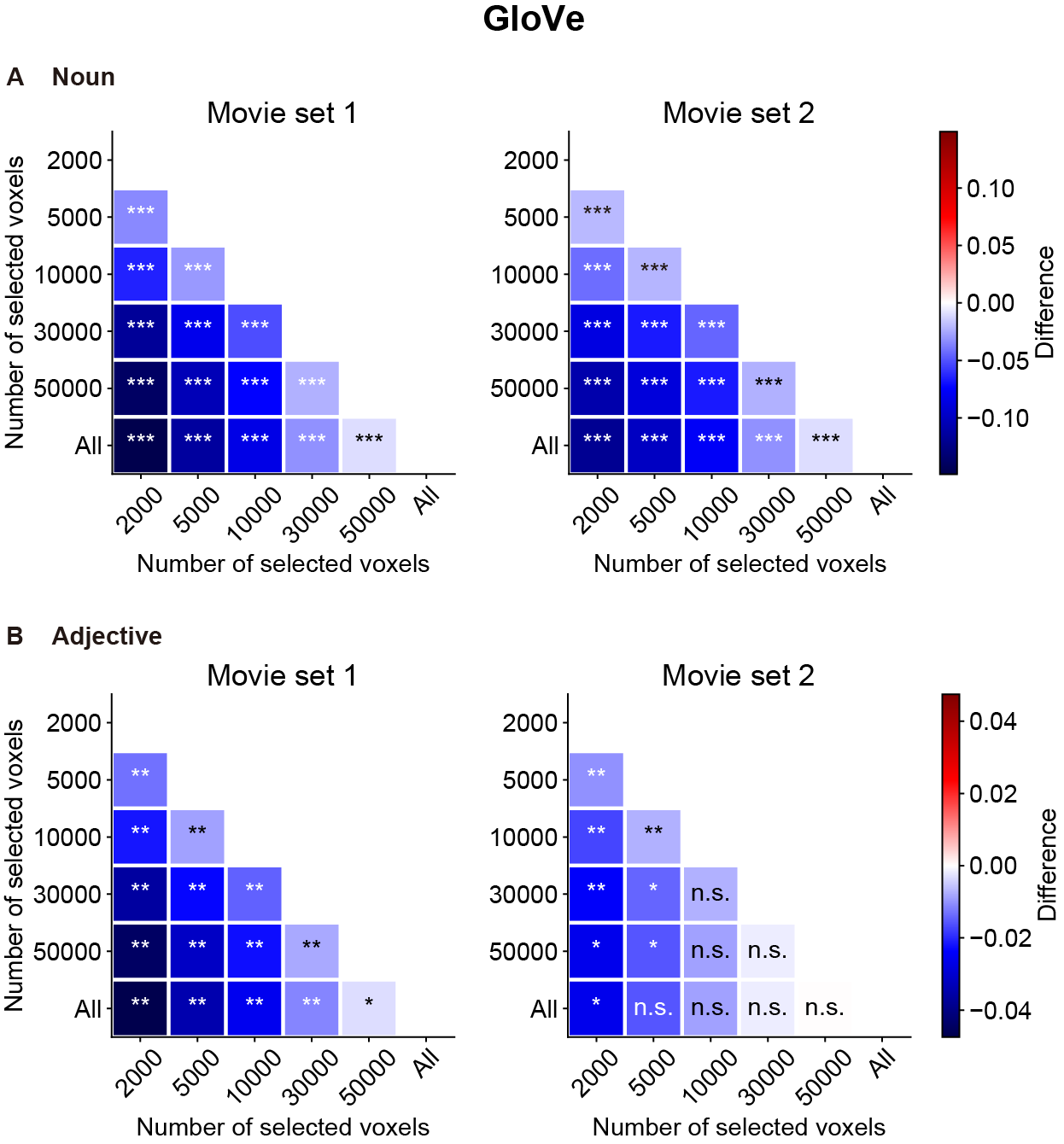

Supplement: S24 Fig — The same analysis as in S23 Fig but for GloVe vectors. (TIF) [file pcbi.1009138.s024.tif]

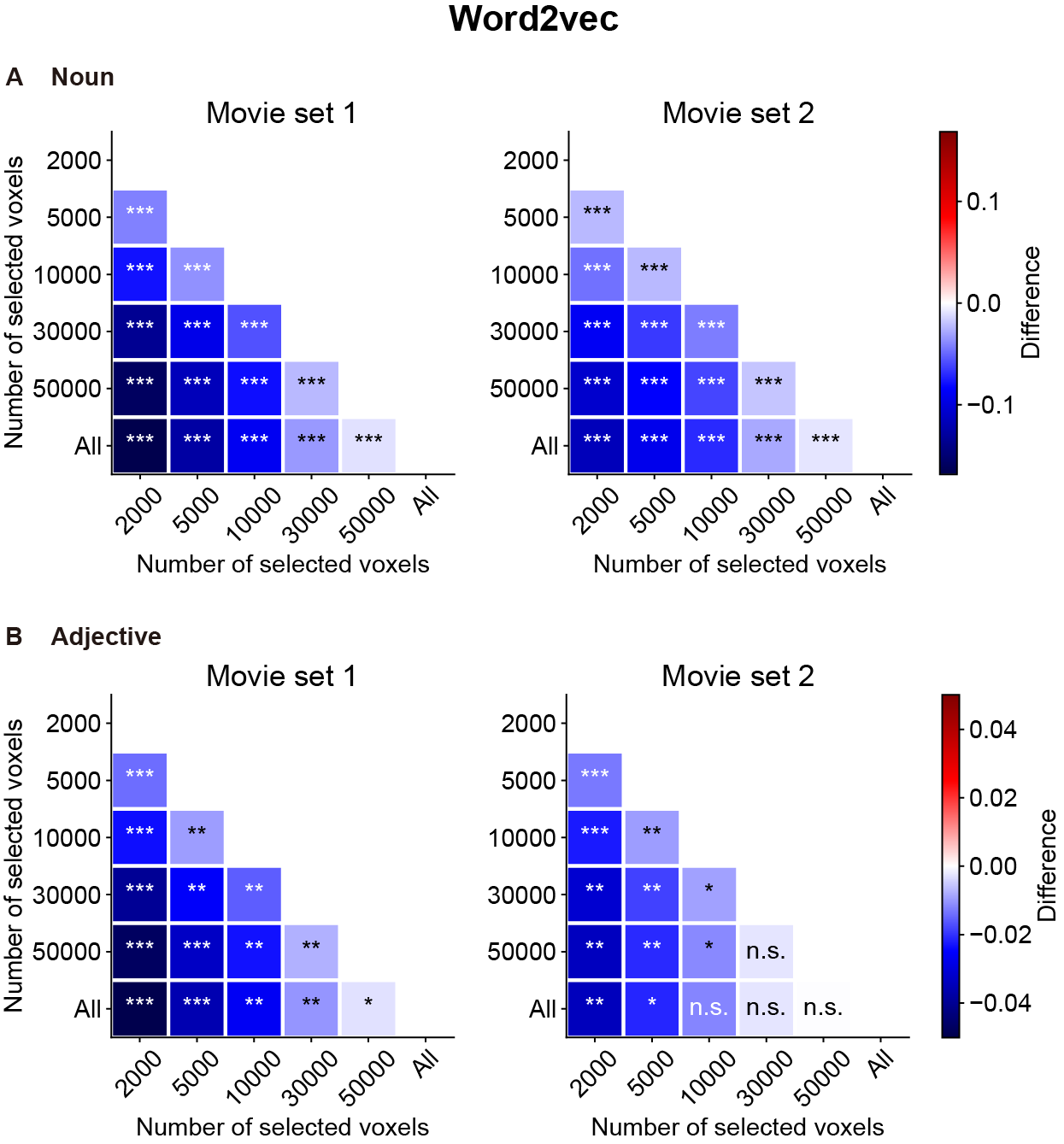

Supplement: S25 Fig — The same analysis as in S23 Fig but for word2vec vectors. (TIF) [file pcbi.1009138.s025.tif]

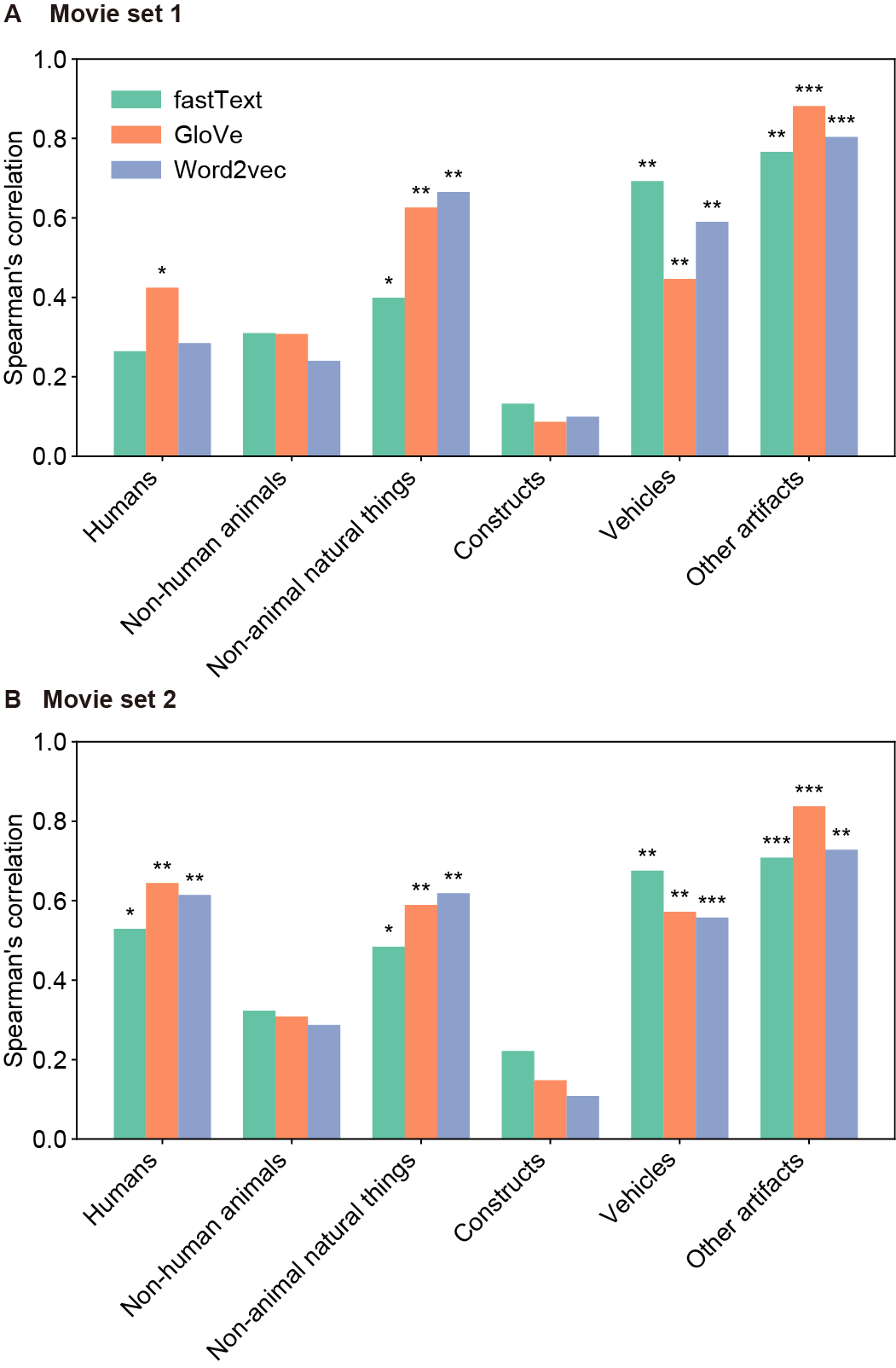

Supplement: S26 Fig — The nouns used for constructing word dissimilarity matrices consisted of the six categories (humans, non-human animals, non-animal natural things, constructs, vehicles, and other artifacts; 10 nouns in each category). We calculated the correlation between behavior- and brain-derived word dissimilarity matrices obtained from each noun category. The Pearson’s correlation coefficients for each category were shown separately for each model (green, fastText; orange, GloVe; blue, word2vec) and for each movie set (A, movie set 1; B, movie set 2). Marks above bars indicate the statistical significance of the correlation coefficients (permutation test, ***p < 0.0001, **p < 0.01, *p < 0.05, FDR corrected). (TIF) [file pcbi.1009138.s026.tif]

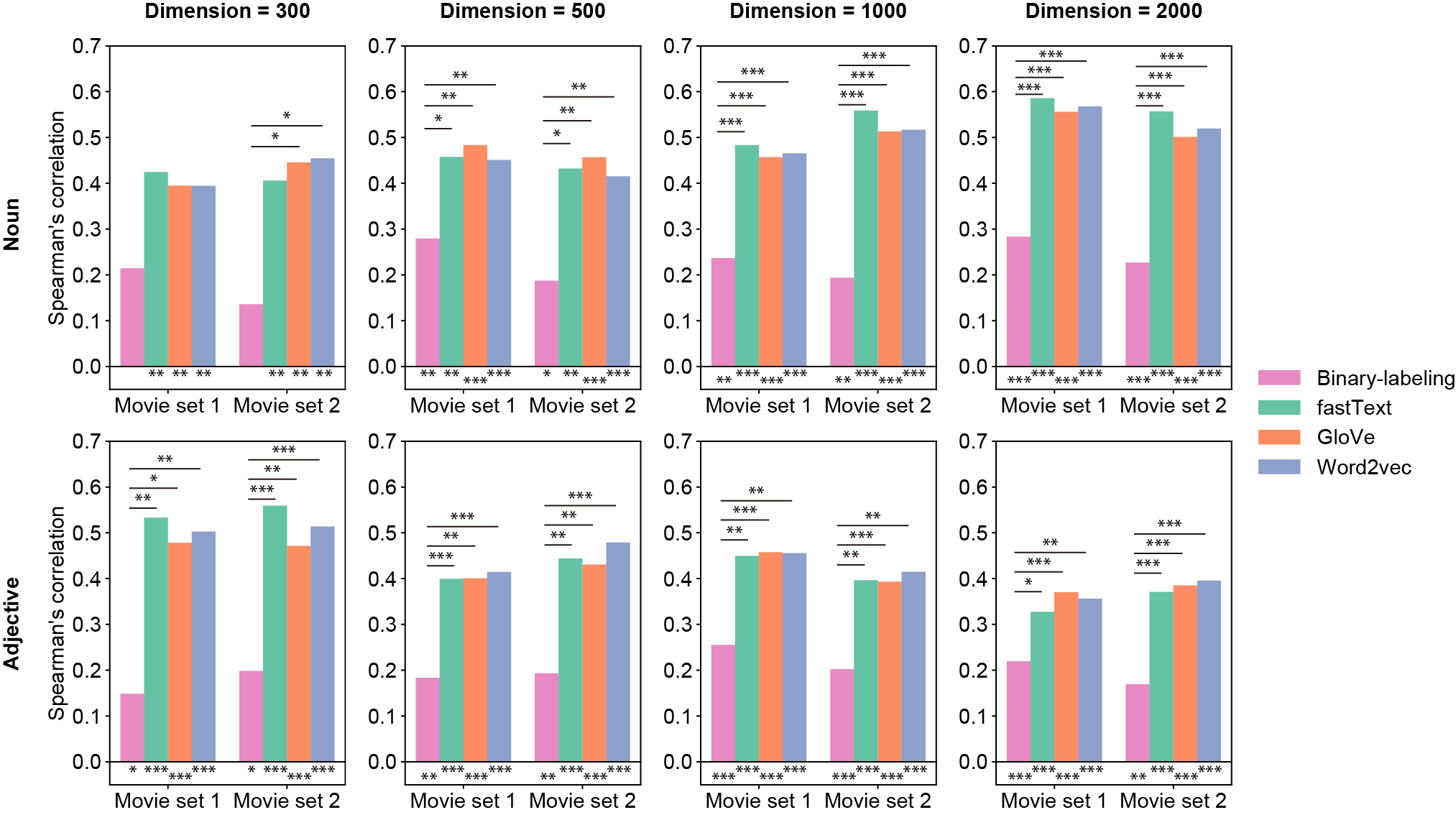

Supplement: S27 Fig — The correlation of brain- and behavior-derived word dissimilarity matrices was computed for binary-labeling models. The correlation coefficients for these models (pink bars) are separately shown for nouns (top) and adjectives (bottom) and for the vector dimensionality of 300, 500, 1000, and 2000 (from left to right), along with the coefficients of brain–behavior correlation for the word vector-based models (green bars, fastText; orange bars, GloVe; blue bars, word2vec). In this case, because the vocabulary of the binary-labeling models changed depending on vector dimensionality and datasets (S16 Table), the number of nouns and adjectives used for calculating the brain-behavior correlation of both binary-labeling and word vector-based models were different across vector dimensions and datasets. Consequently, the correlation coefficients for the word vector-based models differed from those shown in other figures (Figs 6–7 and S18–S20). Marks below bars indicate the statistical significance of correlation coefficients above chance level whereas marks above bars indicate the statistical significance of the correlation difference between the binary-labeling model and each of the word vector-based models (permutation test, ***p < 0.0001, **p < 0.01, *p < 0.05, FDR corrected). (TIF) [file pcbi.1009138.s027.tif]

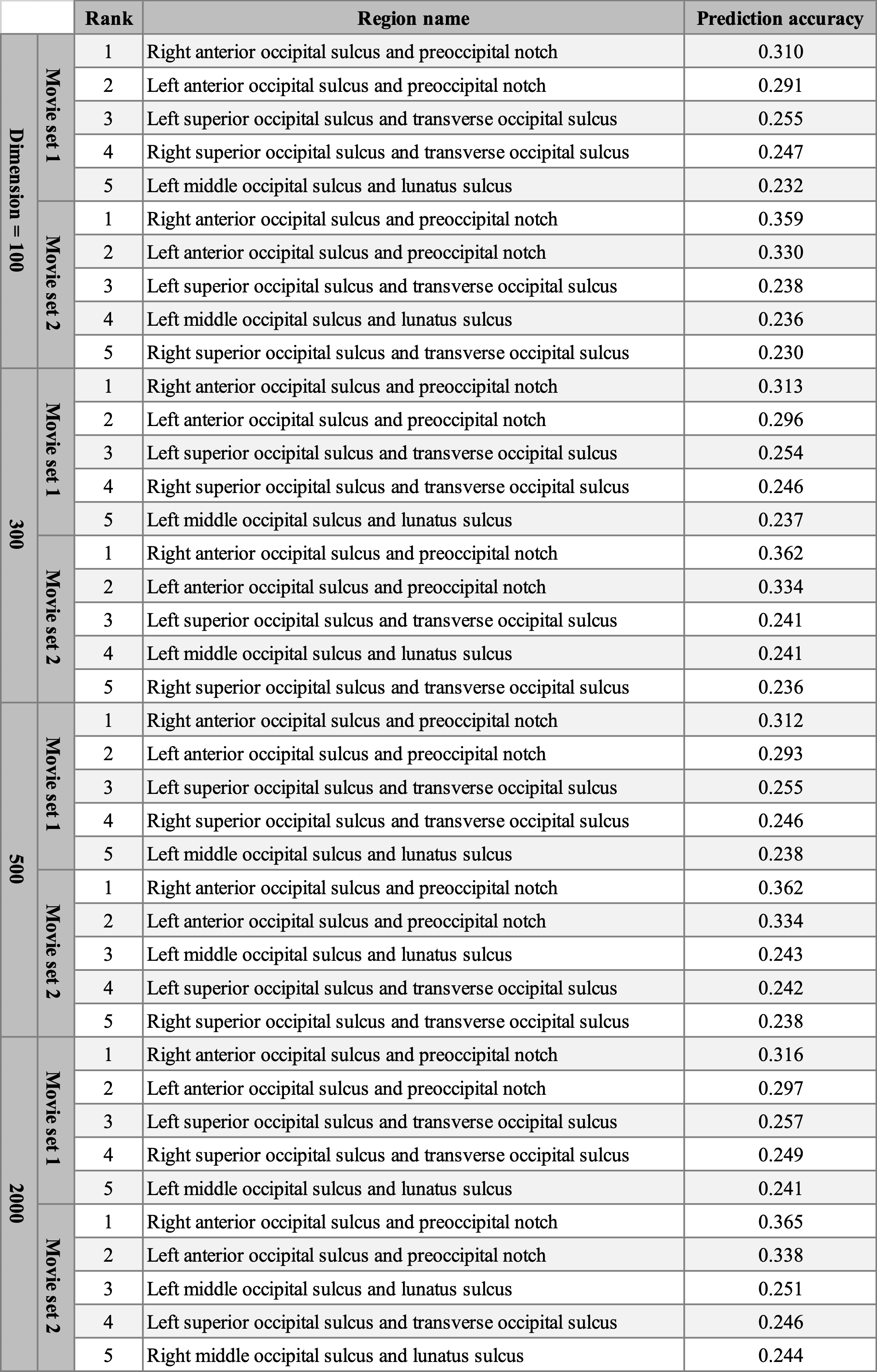

Supplement: S1 Table — (TIFF) [file pcbi.1009138.s028.tiff]

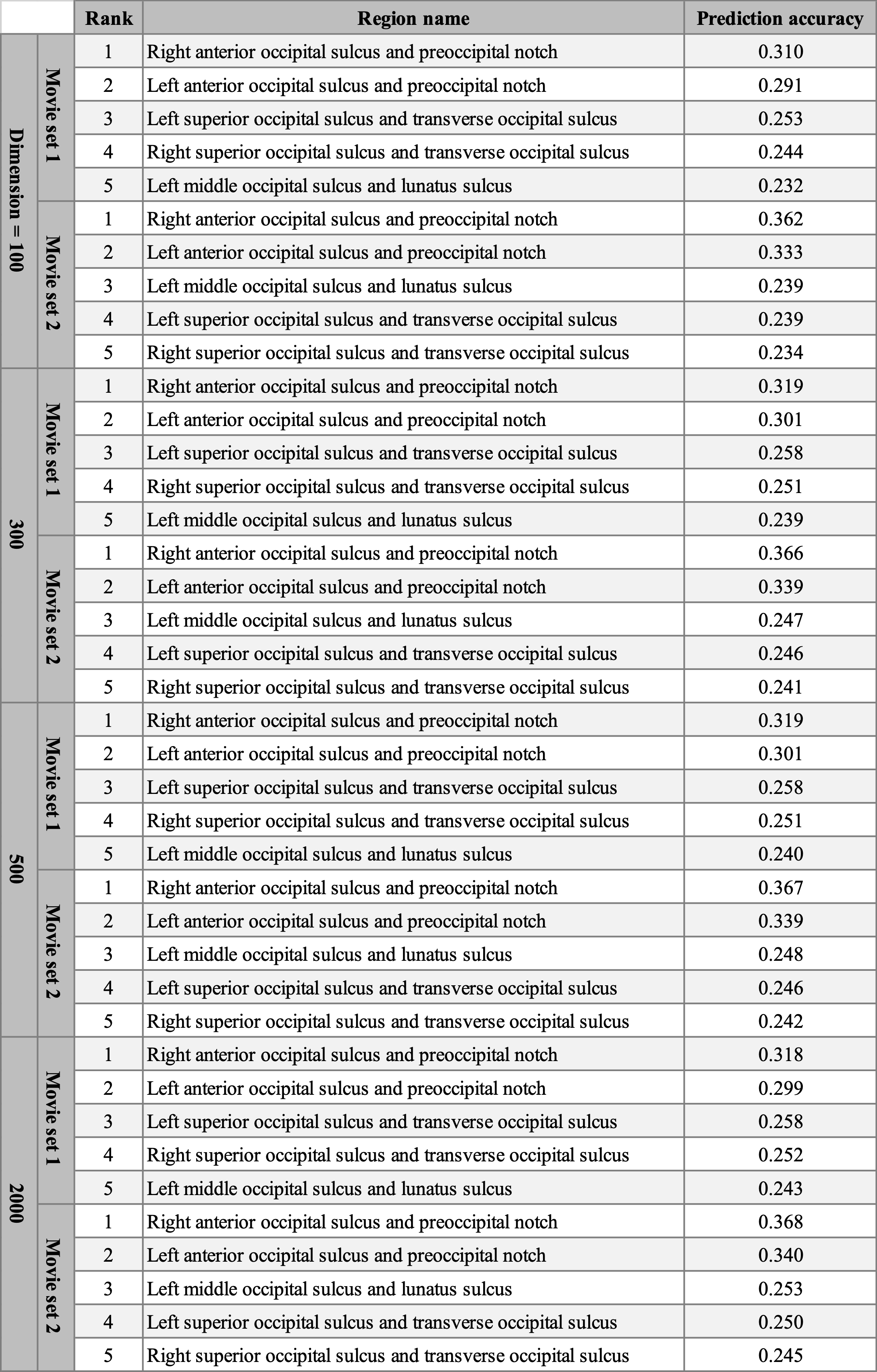

Supplement: S2 Table — (TIFF) [file pcbi.1009138.s029.tiff]

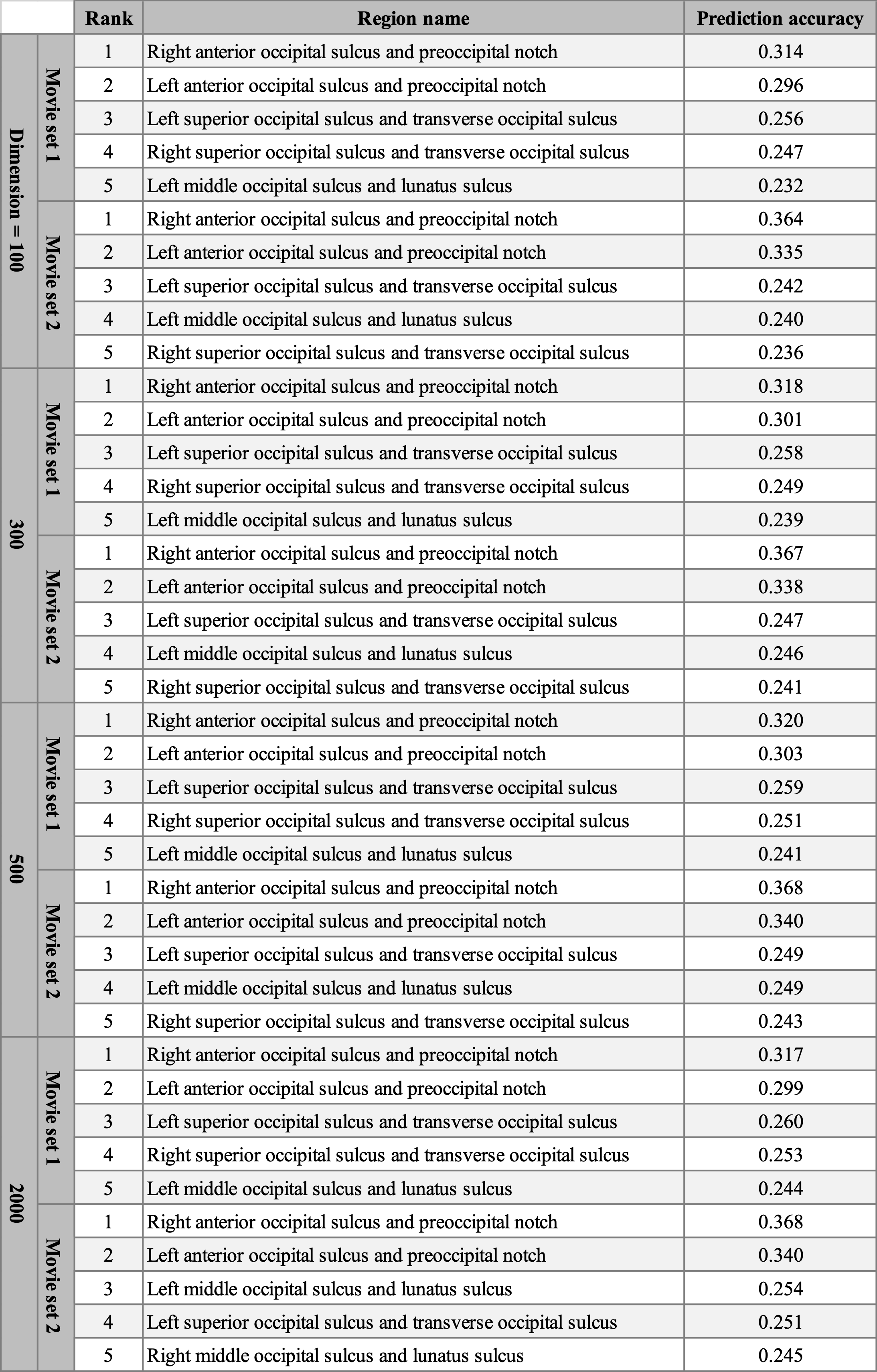

Supplement: S3 Table — (TIFF) [file pcbi.1009138.s030.tiff]

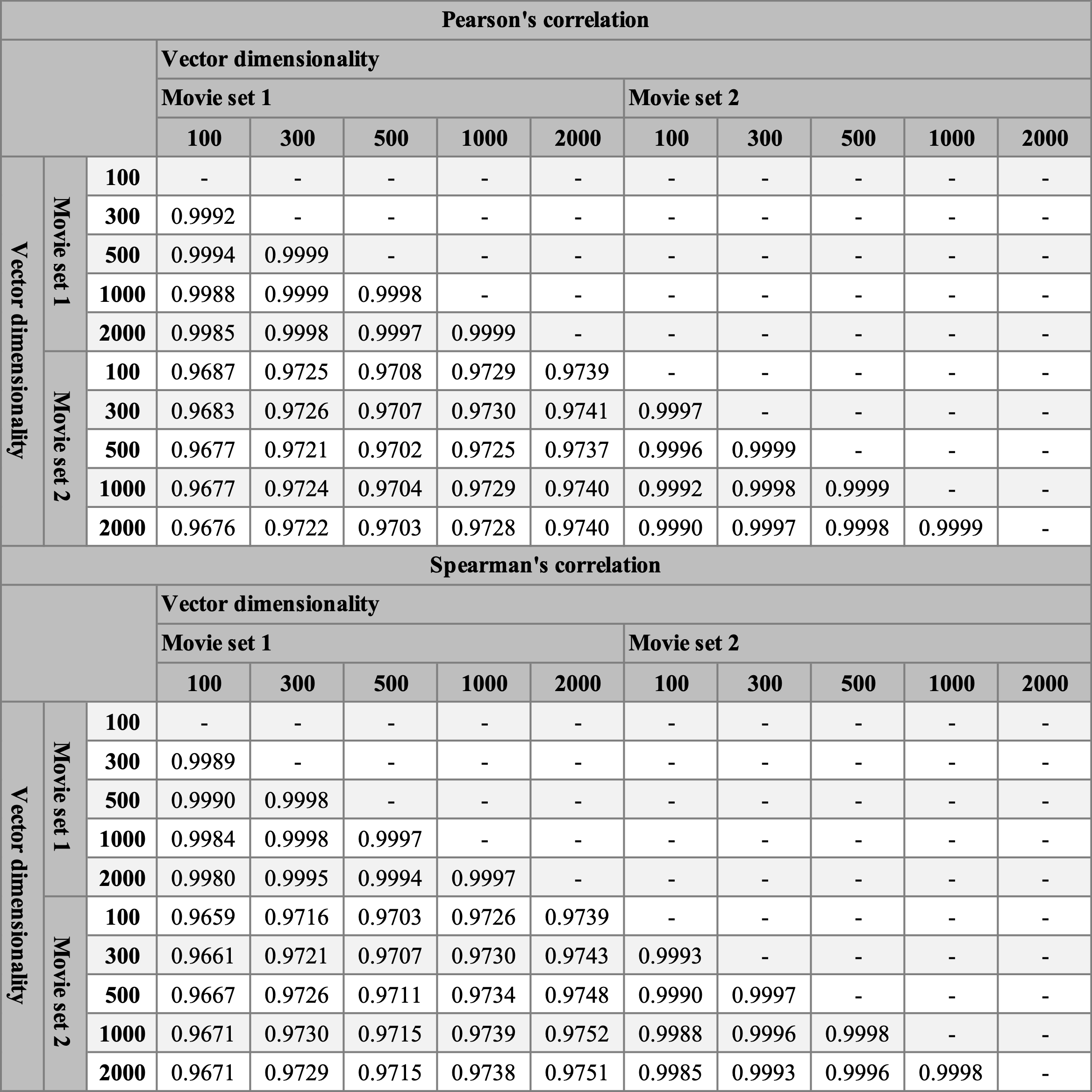

Supplement: S4 Table — We calculated the mean prediction accuracy of fastText vector-based models within each cortical region (Figs 4 and S6) and compared the inter-regional patterns of prediction accuracy between arbitrary pairs of vector dimensionality and movie sets. The value in each cell in the upper and lower part of the table denotes the Pearson’s or Spearman’s correlation coefficient, respectively, of the inter-regional patterns between each pair. (TIFF) [file pcbi.1009138.s031.tiff]

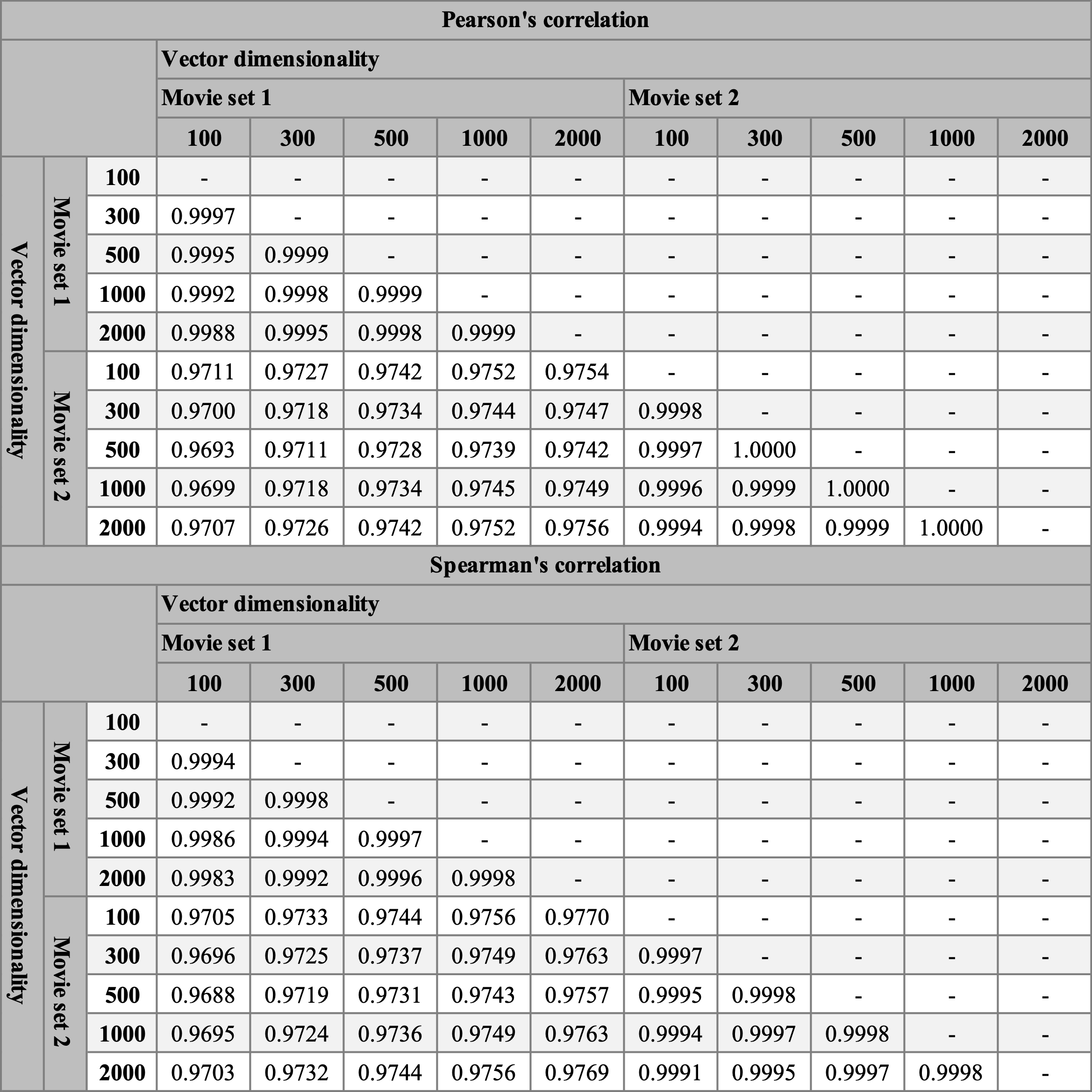

Supplement: S5 Table — The same analysis as in S4 Table but for GloVe vectors. (TIFF) [file pcbi.1009138.s032.tiff]

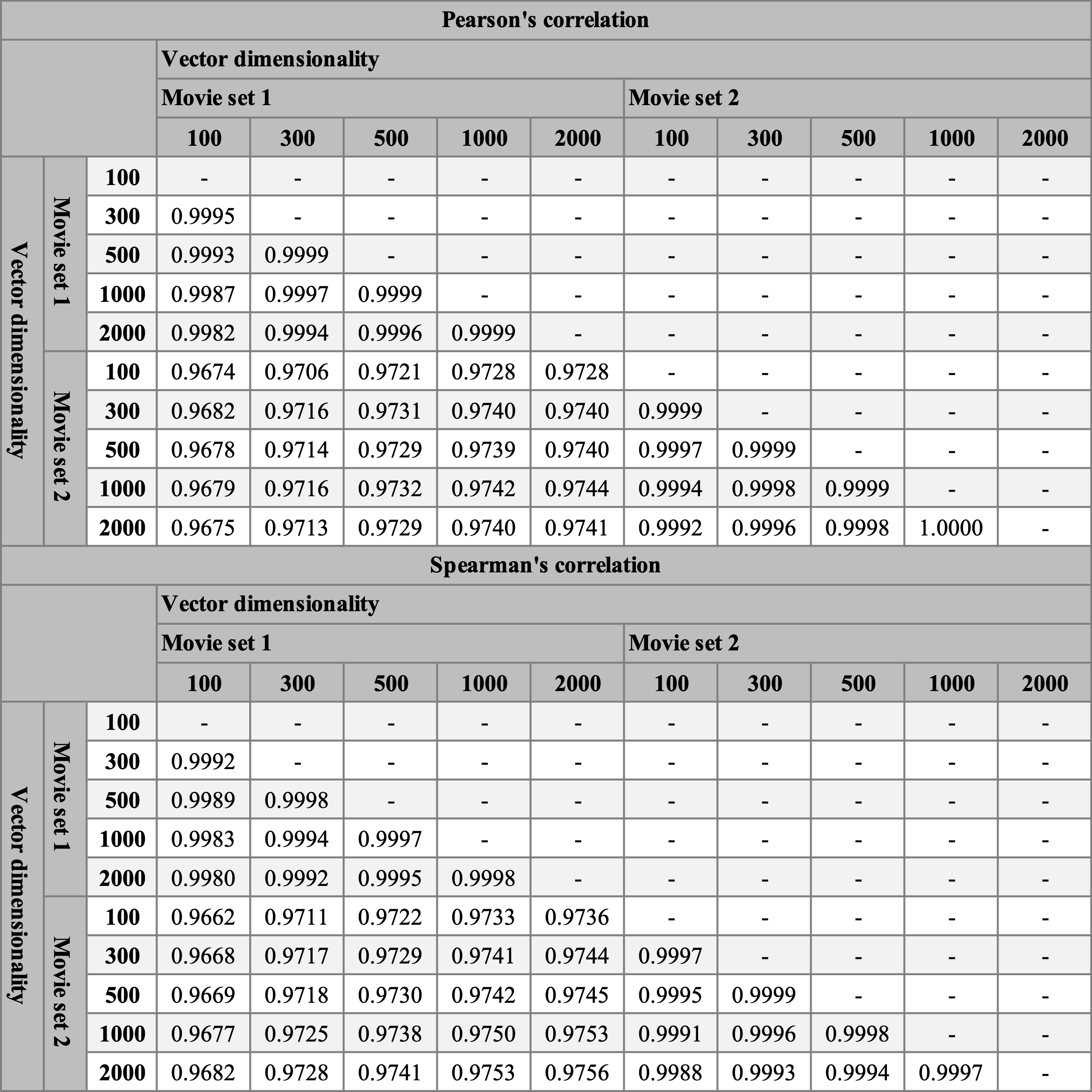

Supplement: S6 Table — The same analysis as in S4 Table but for word2vec vectors. (TIFF) [file pcbi.1009138.s033.tiff]

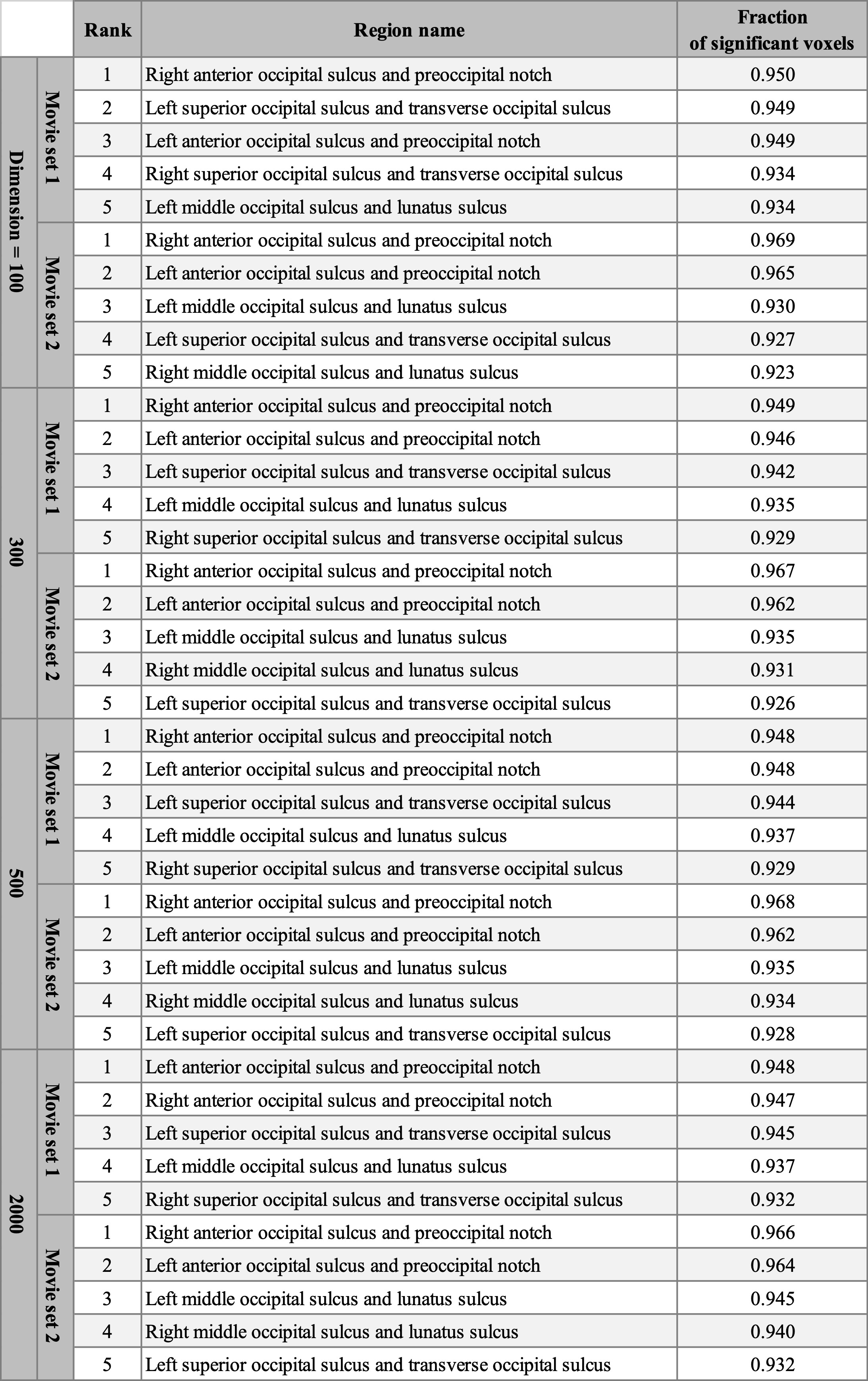

Supplement: S7 Table — (TIFF) [file pcbi.1009138.s034.tiff]

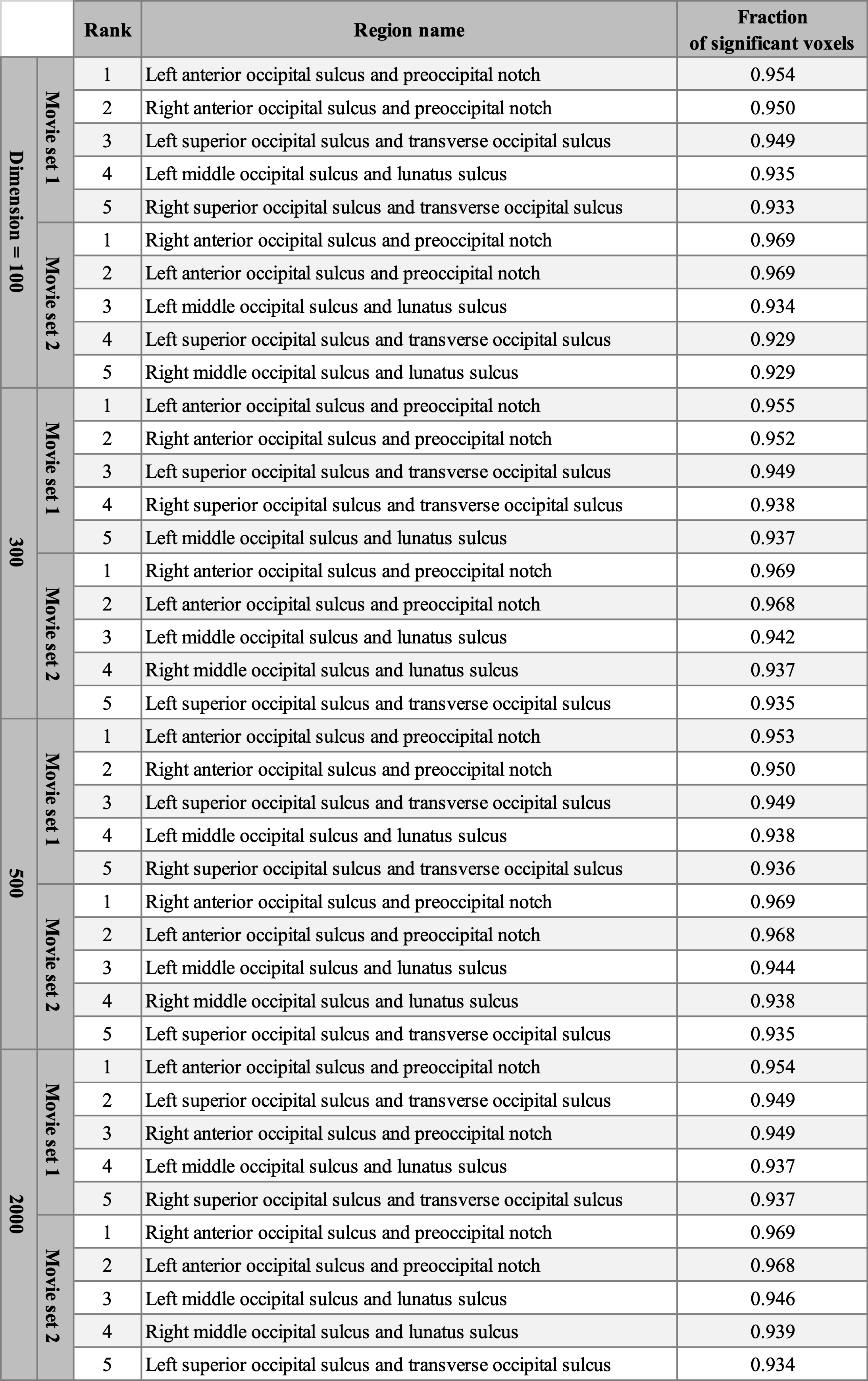

Supplement: S8 Table — (TIFF) [file pcbi.1009138.s035.tiff]

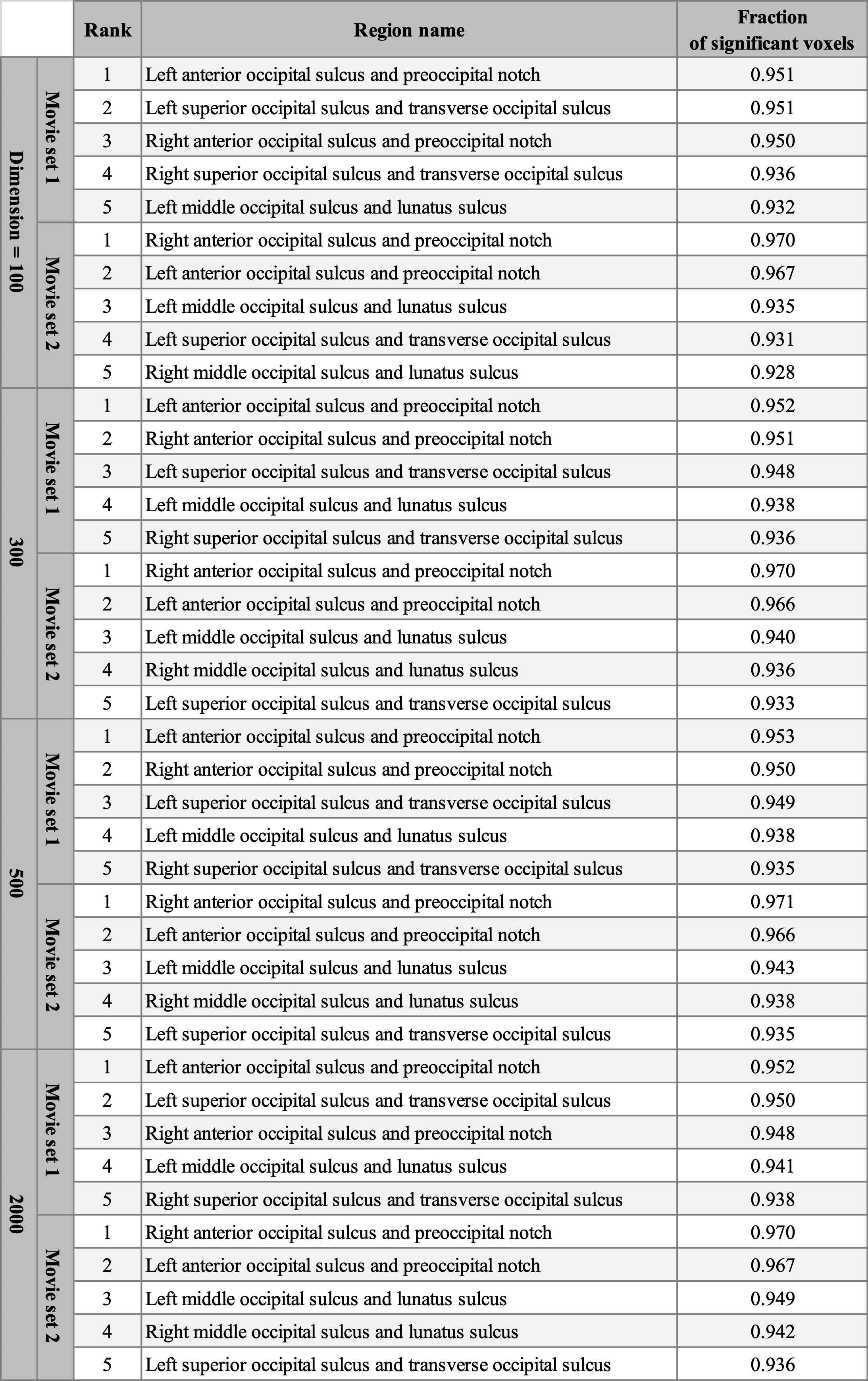

Supplement: S9 Table — (TIFF) [file pcbi.1009138.s036.tiff]

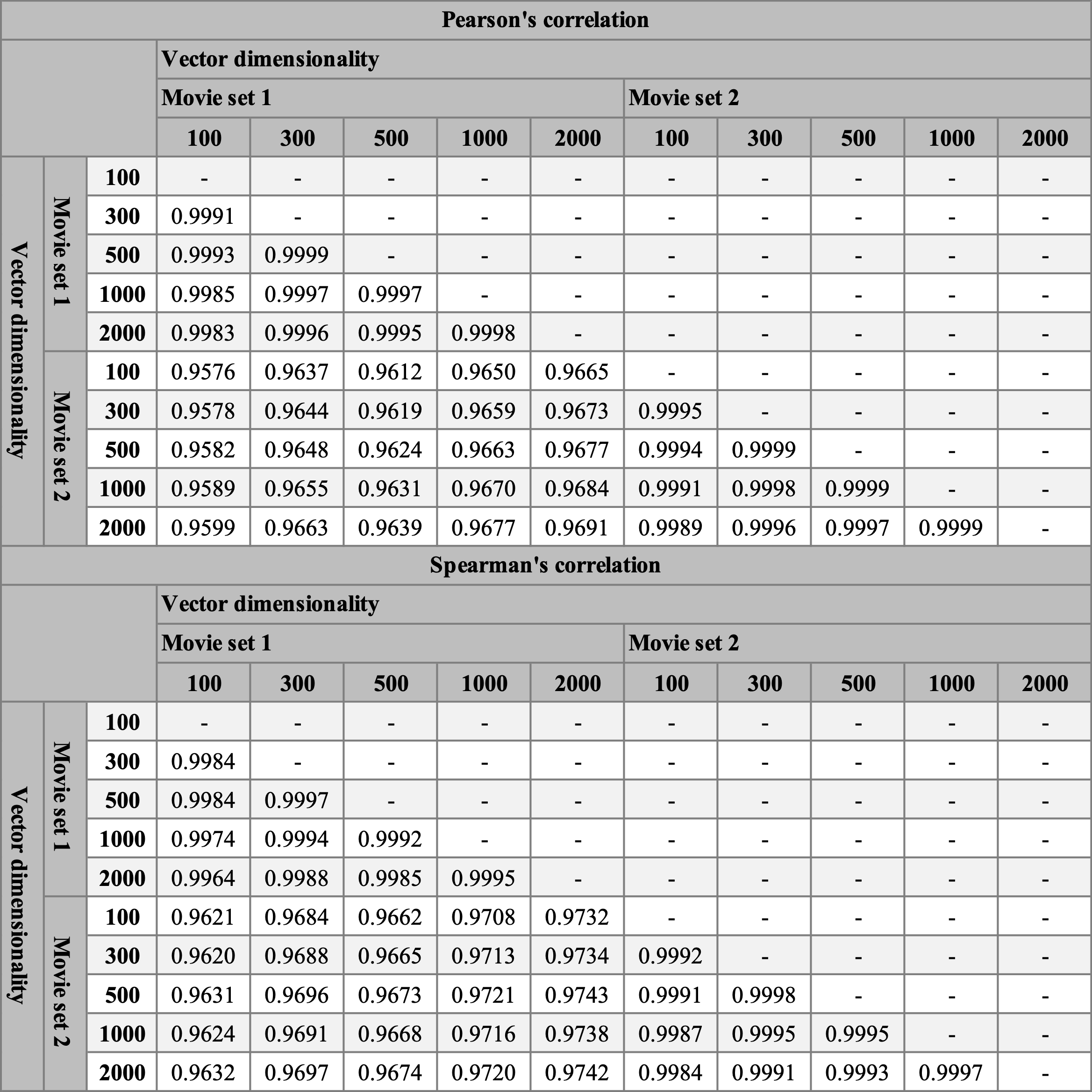

Supplement: S10 Table — As in the case of the mean prediction accuracy (S4 Table), the inter-regional patterns of significant-voxel fraction of fastText vector-based models (Figs 5 and S11) were compared between arbitrary pairs of vector dimensionality and movie sets. The value in each cell in the upper and lower part of the table denotes the Pearson’s or Spearman’s correlation coefficient, respectively, of the inter-regional patterns between each pair. (TIFF) [file pcbi.1009138.s037.tiff]

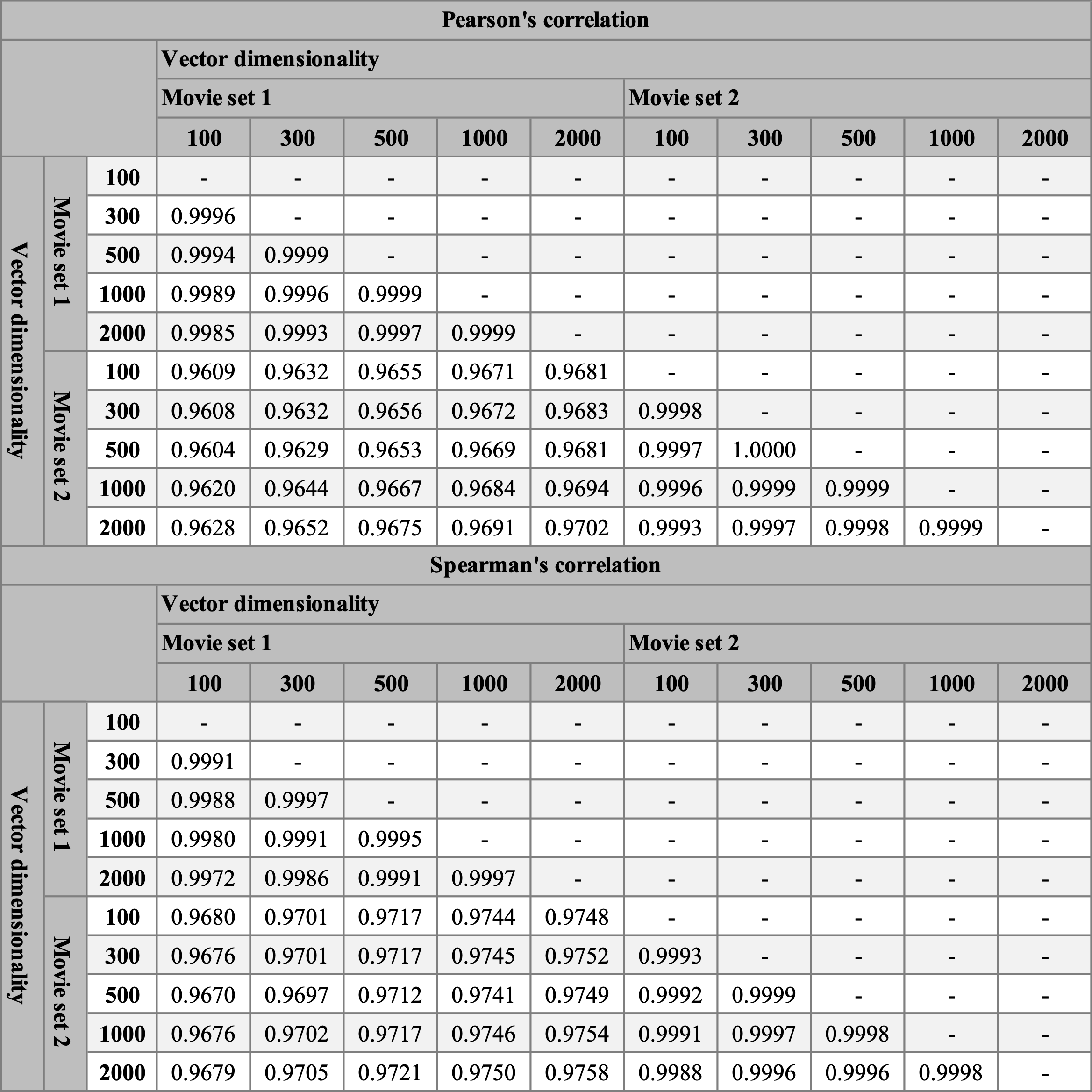

Supplement: S11 Table — The same analysis as in S10 Table but for GloVe vectors. (TIFF) [file pcbi.1009138.s038.tiff]

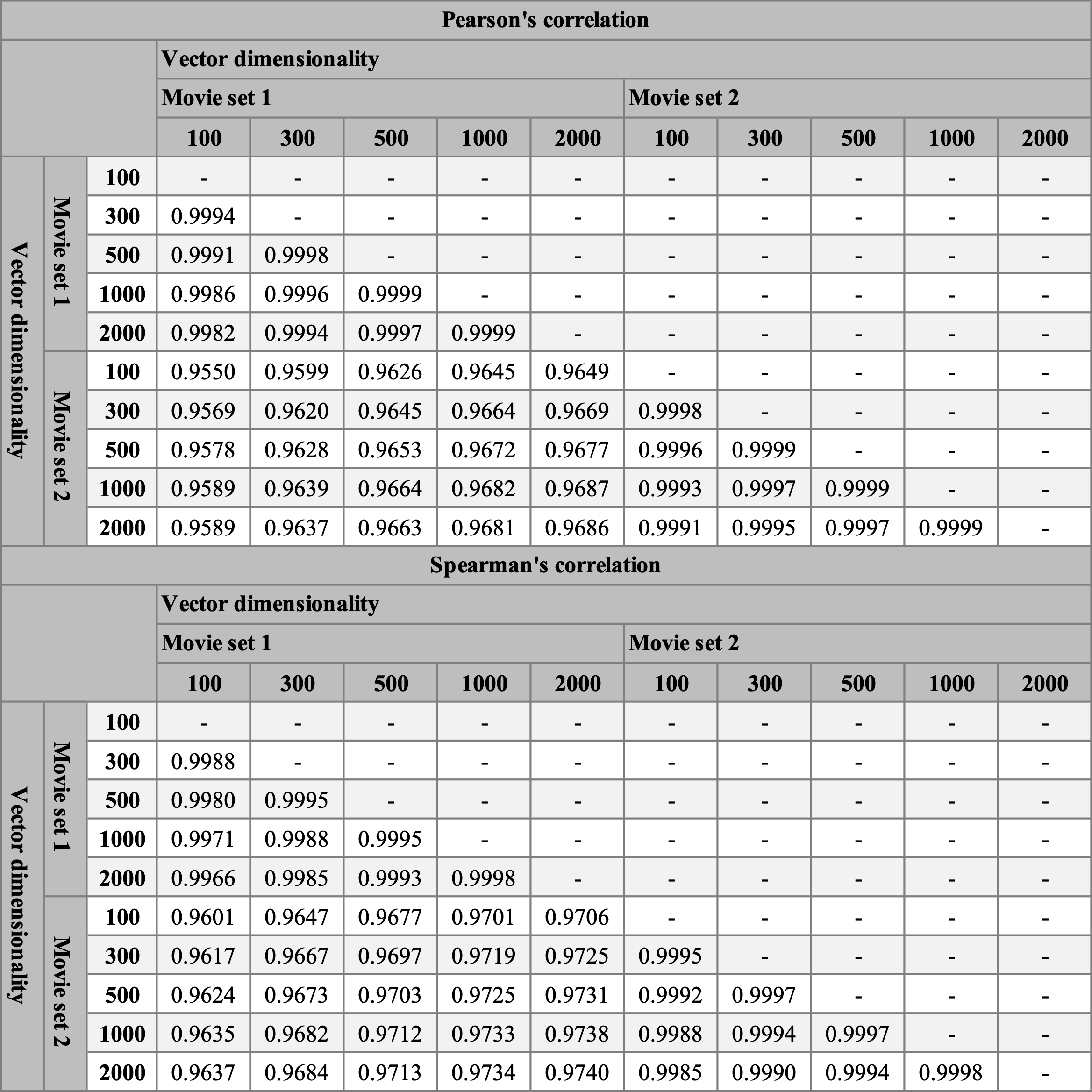

Supplement: S12 Table — The same analysis as in S10 Table but for word2vec vectors. (TIFF) [file pcbi.1009138.s039.tiff]

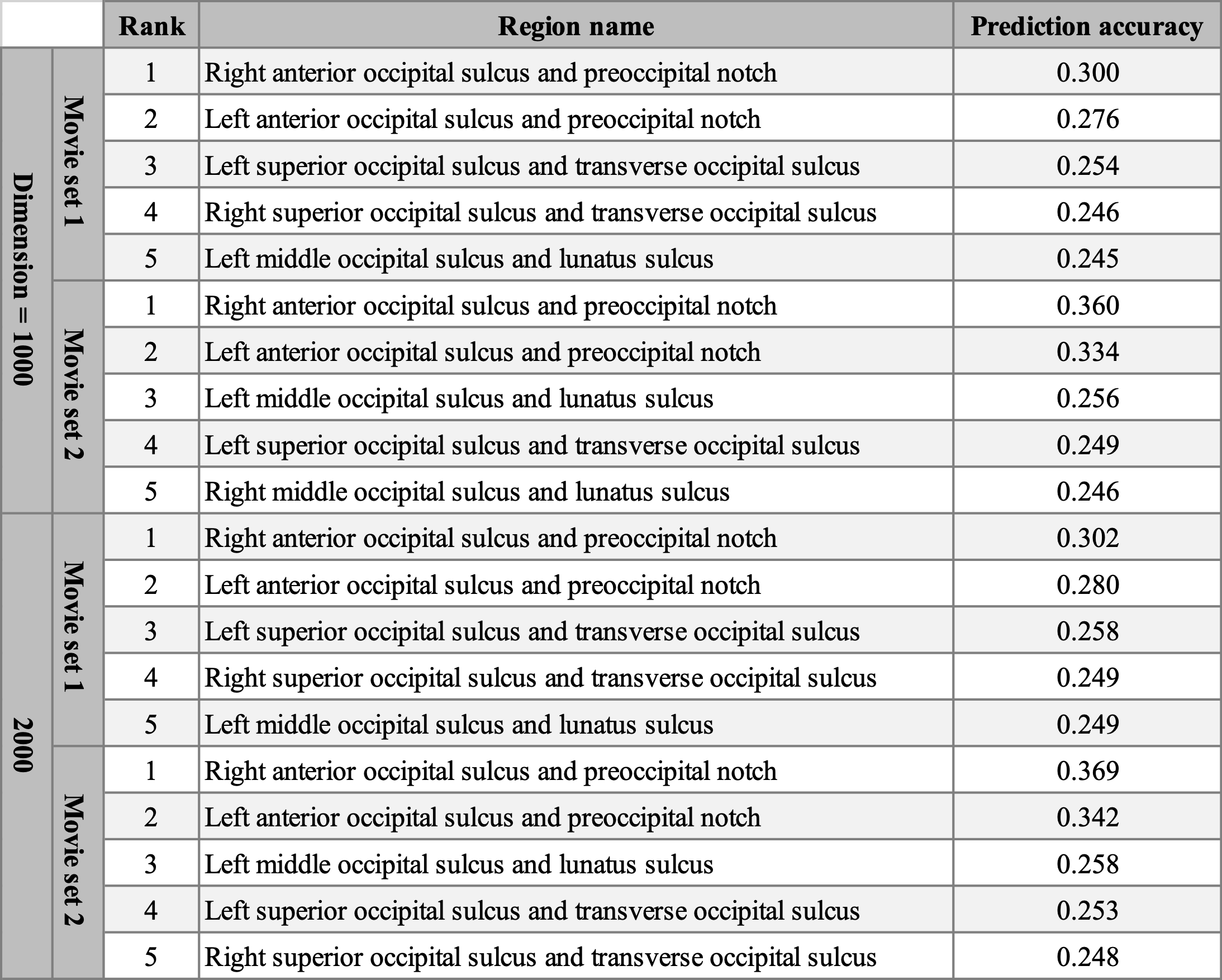

Supplement: S13 Table — (TIFF) [file pcbi.1009138.s040.tiff]

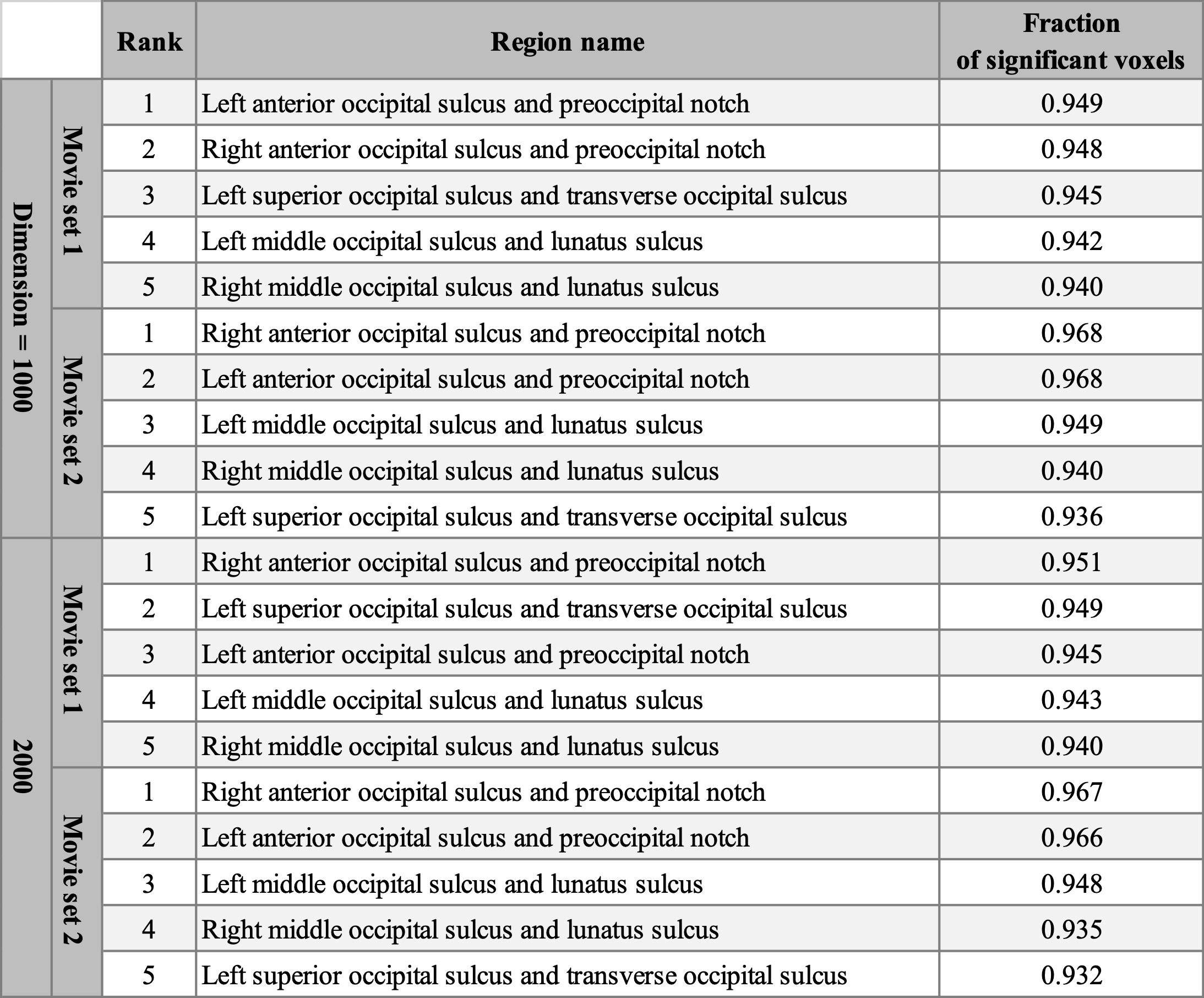

Supplement: S14 Table — (TIFF) [file pcbi.1009138.s041.tiff]

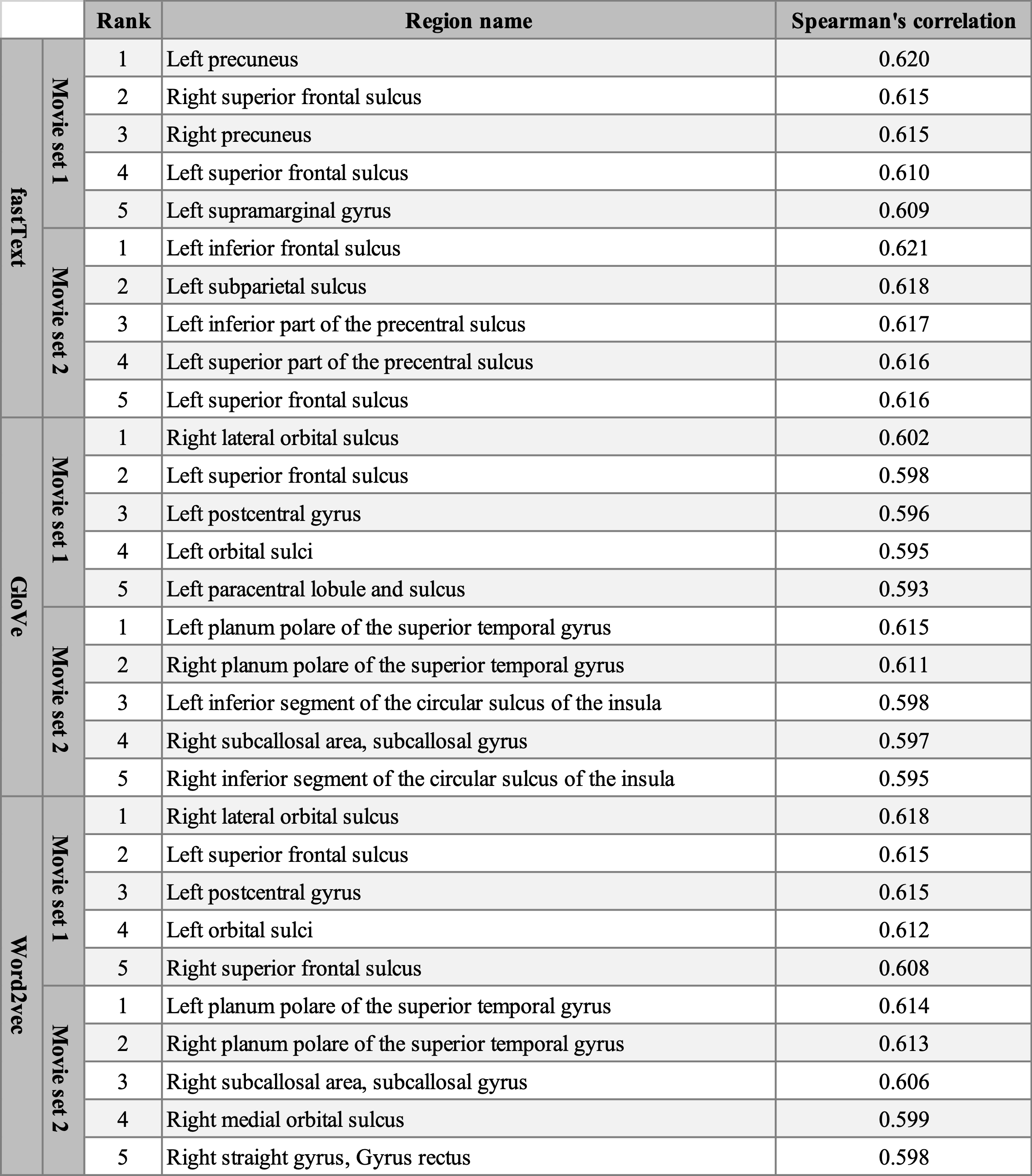

Supplement: S15 Table — (TIFF) [file pcbi.1009138.s042.tiff]

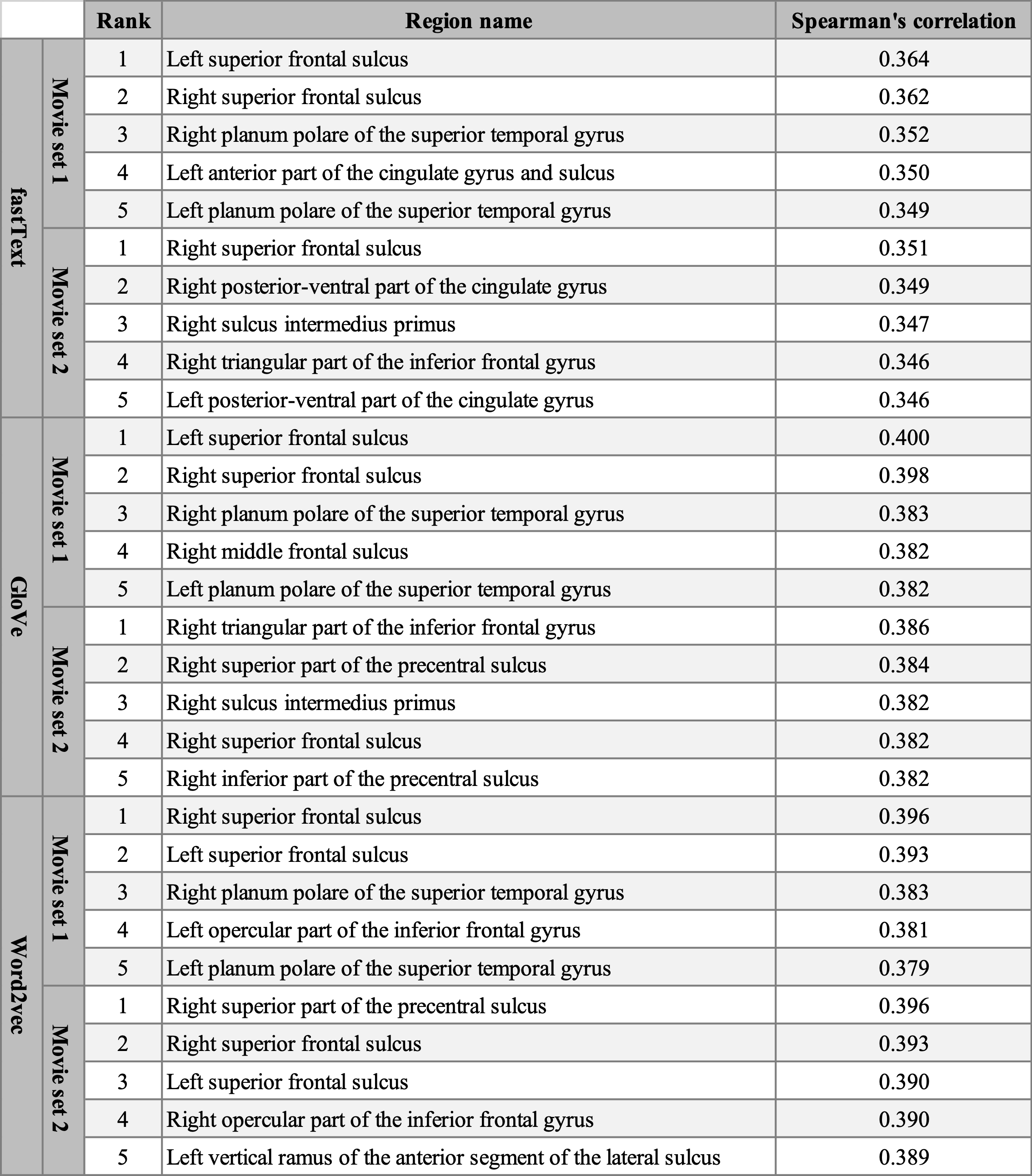

Supplement: S16 Table — (TIFF) [file pcbi.1009138.s043.tiff]

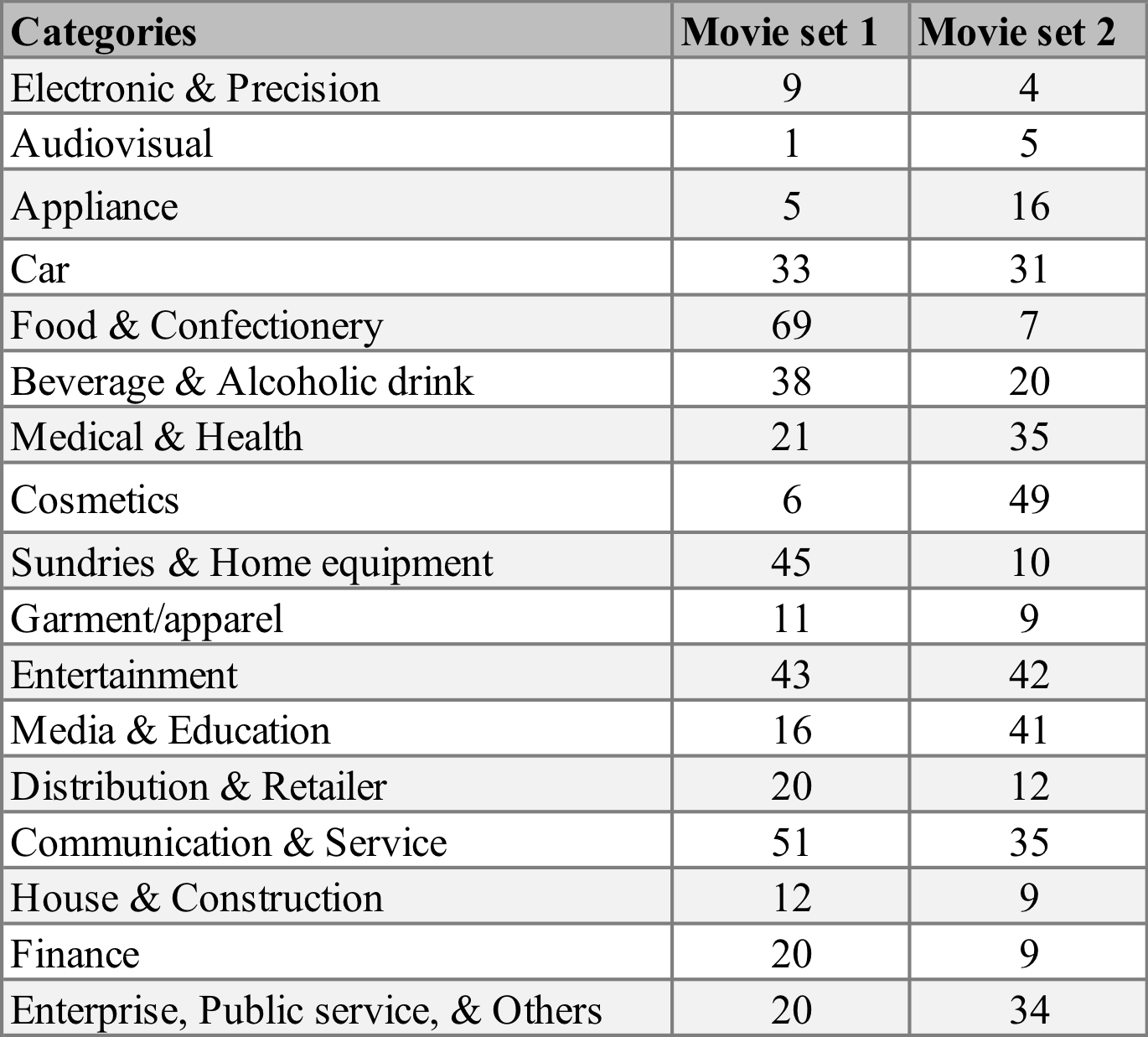

Supplement: S17 Table — (TIFF) [file pcbi.1009138.s044.tiff]

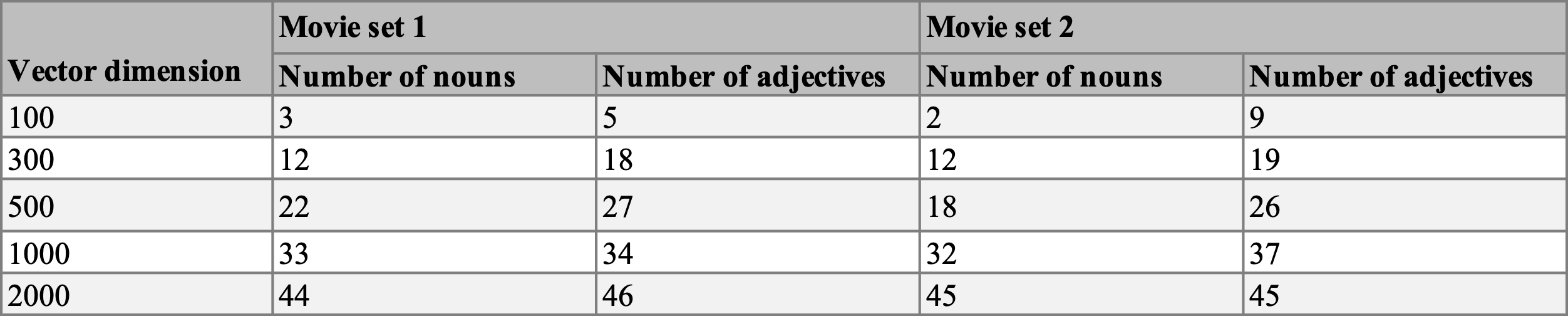

Supplement: S18 Table — (TIFF) [file pcbi.1009138.s045.tiff]
