# Supplementary material for: Behavioral correlates of cortical semantic representations modeled by word vectors
Source: PLoS Comput Biol. 2021 Jun 23;17(6):e1009138. doi: 10.1371/journal.pcbi.1009138 (PMC8260002; doi:10.1371/journal.pcbi.1009138)
Supplement: Attachment — Submitted filename: Nishida-BehavCorrSemantic-ResponseLetter02.docx [file pcbi.1009138.s046.docx]

We appreciate the reviewers for their efforts in helping us to improve this paper. We have revised the manuscript and figures to address all of their concerns. Our point-by-point replies are given here.

**Response to Reviewer #1:**

The authors investigate the extent to which artificial models of language (NLP) can predict human neural responses and align with human behavioral judgments. They find significant agreement between brain, model, and behavior and conclude that current word embedding models are able to capture a meaningful amount of variance of both neural representation and semantic perception in humans.

**Overall Impressions**

I think this question is very interesting and I think that progress in this domain would be very welcome and useful for everyone who is interested in semantics, neural correlates of perception, and/or prediction of neural representations (myself included). I also like the approach (which is consistent with prior work), but I’m not fully convinced that the present results provide significant new insights into the problem in their current form. My main concerns about the current manuscript relate to the choice of embedding model, the lower effect sizes compared to similar prior work, and not fully accounting for known neural biases in how the effects are analyzed. However, I believe that these concerns could be fully addressed by additional analyses and discussion (as detailed below).

**Major Comments**

(1) The embedding model selected has weaker performance than openly available alternatives: Pereira et al. 2018 shows that multiple skip-gram models (e.g., word-2-vec, which is purportedly not as ‘good’ as fastText) can predict concept similarity up to r~=0.70. There’s also some recent work from Iordan et al. 2019 that pushes this to r~=0.90 for certain categories. In the current work, fastText is only at r~=0.30 with behavior and the authors argue that this means the brain mapping helps (r=~0.60), but it may be that other word embedding models can already do much better than any brain mapping at predicting behavior for this particular set of adjectives and/or nouns, but that the authors did not pick a good enough embedding model. To provide evidence that this is not the case, the authors could (1) test other embedding models listed above and show that they have similar behavioral performance for their set of concepts; and/or (2) show that at least one such other model (especially if it outperforms fastText) can also give improved predictions when used to predict neural representations similar to the current analysis.

**[Response]** We appreciate the insightful comment. Following the comment, we tested other embedding models (Glove and word2vec skip-gram) in our analysis and found that the correlation between the behavior- and word vector-derived dissimilarity matrices improved (Pearson r ≥ 0.6 for nouns and r ≥ 0.4 for adjectives) when these embedding models were used instead of fastText skip-gram. The Pearson correlation coefficients for nouns were comparable with those reported in Pereira et al. 2016 (we guess that the previous work the reviewer mentioned was Pereira et al. 2016, Cognitive Neuropsychology 33:175–190, but not Pereira et al. 2018). Please see Figs 7 and S16–18 Figs in the revised manuscript. Note that we have used Spearman correlation instead of Pearson correlation in the revised manuscript, following a suggestion from another reviewer.

Although Iordan et al. 2019 reported further higher behavioral correlations (~0.9), it is not surprising because they tested the behavioral correlations of semantic structures in specific categories using word embedding models specialized for the categories, as the reviewer also mentioned. Behavioral correlations should be affected not only by the choice of words, as suggested by the variable results in Pereira et al. 2016 depending on the datasets of human similarity judgements, but also by paradigms for collecting similarity judgements in psychological experiments. However, as we mentioned in Psychological experiments subsection in Methods section, Kriegeskorte et al. 2012 argued that the paradigm we also used for collecting similarity judgements was more efficient for estimating perceptual dissimilarity structure of semantic entities from participants than the other paradigms. Therefore, we believe that our dataset of human similarity judgements is appropriate for testing behavioral correlations of word dissimilarity structures.

Importantly, even when using GloVe and word2vec as well as fastText, we observed significant correlations between behavior- and brain-derived dissimilarity matrices (p < 0.0001). This indicates that the main conclusion in our work (i.e., behavioral correlates of cortical semantic representations modeled by word vectors) remains intact regardless of embedding methods. Please see Fig 7 and S19 Fig in the revised manuscript.

It should be noted that although the word vector-behavior correlation for fastText vectors was much lower than those for the other vectors, the brain-behavior correlation for fastText vectors was rather higher for nouns and was not much different for adjectives compared with the other vectors (please see Fig 7a in the revised manuscript). This result indicates that the ability of fastText vectors for explaining the similarity structure of cortical semantic representations is not poorer than the other vectors.

To show the results for the other embedding methods and the comparisons between methods, we have added/modified figures (Figs 1, 3–4, and 7–8; S2–4, S6–7, S11–12, S17–19, and S22–23 Figs) and tables (S5–6 and S11–12 Tables) and updated the descriptions in Introduction (lines 79–88), Results (lines 132–136, 138–140, 155–164, 208–209, and 325–334), and Methods (lines 630–637 and 646–648) sections.

(2) Neural pattern prediction accuracy is much lower and spatially different from prior work: The pattern of model performance (Fig. 4, top) is very different from that of prior work and performance is much lower (i.e., r~=0.10-0.30 compared to e.g. Fig. 1 of Huth et al. 2016, r=0.60 in high-level regions). Spatially, in the present work, prediction is highest in LO, which is below chance in the Huth language model; also, current model has little to no significant frontal cortex predictions. One could argue that this is because Huth et al. 2016 tries to use a language model to predict auditory stories, not movies. But then, performance of the current model is even weaker when compared to Huth et al. 2012 (r values up to 0.80) which uses simple binary labelling of the objects occurring in videos to predict neural response. Hence, I am not convinced that this language model is the best approach to model visual content, e.g., complex movies. To provide evidence that this is a good/comparable/better alternative than existing methods, the authors could use their current data to automatically generate a binary matrix of objects (similar to Huth et al. 2012) for every timepoint and use this to perform neural prediction, instead of using the word vector representations for the objects/concepts. If this matches/improves performance, then this could show evidence that the word vector approach either doesn’t add anything or may even hinder recovery of semantic information by introducing external biases.

**[Response]** We appreciate the helpful suggestion. Following the suggestion, we modeled brain response using binary labels of semantic contents in movie scenes and compared the model performance with the word vector-based models. The binary labels were obtained from the presence/absence of words in the manual descriptions for each movie scene. We constructed 100-, 300-, 500-, 1000-, and 2000-dimensional scene vectors using the binary labels of 100, 300, 500, 1000, and 2000 words that most frequently appeared in the movie-scene descriptions for each movie set. We found that voxelwise models based on the binary labels (binary-labeling models) showed significantly higher performance of brain-response prediction compared with the word vector-based models. However, the performance difference between these models seems to be too small to explain the performance difference between the current and previous (Huth et al. 2012; 2016) models. Please see S4, S14, and S15 Figs and the descriptions in the Results (lines 172–194 and 280–284) and Methods (lines 677–690) sections.

Instead, the performance difference from the previous models is likely to be explained by the difference in the granularity of prediction analysis (i.e., voxel-wise vs. ROI-wise). Following the reviewer’s comment #7 (please see below), we evaluated voxelwise prediction accuracy for the word vector based-models mapped onto the cortical surface of a representative participant and found that the prediction accuracy was ranged up to 0.709 (please see S8 Fig in the revised manuscript). However, this accuracy mapping onto a flattened cortical surface needed to be performed for each participant, which means that we have to make 52 maps in total for showing the results from all the participants. Instead, as an additional analysis, we evaluated the maximum values of voxelwise prediction accuracy for each participant and found that the maximum values typically fell within 0.6–0.7 (mean values were around 0.65) regardless of word-vector types and datasets (please see S9 Fig in the revised manuscript). These values were not quite different from those observed in the previous study (Huth et al. 2012; 2016) although the previous studies applied a value correction (noise-ceiling correction) that raises the estimation of prediction accuracy. We have described these results in the Results section (lines 235–243).

Although not the focus of the reviewer’s concern, the comparison of brain­–behavior correlations of word dissimilarity between the binary-labeling and word vector-based models revealed a remarkable difference between these models. Although the cortical localization patters of prediction performance were highly consistent between these models, the brain-behavior correlations for the binary-labeling models were much lower than those for the word vector-based models (please see S25 Fig in the revised manuscript). This result suggests an advantage of voxelwise modeling based on word vectors with regard to capturing the similarity structure of cortical semantic representations associated with human perception. We have described this analysis in the Results (lines 440–447) and Methods (lines 803–811 and 831–833) sections.

(3) Correlations don’t control for the known animate/inanimate distinction in VTC: One of the most salient distinctions in patterns of activity in occipito-temporal cortex is between animate and inanimate stimuli/categories. From the list on line 522, I see a clear human-animal-manmade clustering of nouns which is also consistent with the behavioral matrix in Fig. 4. If the model simply picks up on the highest variance dimension of representation across cortical patterns (e.g., animacy), then this could drive most of the correlation effects observed. However, we already know this animacy effect is present in the brain (Connolly et al. 2012, Konkle & Caramazza 2013, Kriegeskorte et al. 2008, etc.), so, in this case, this model would tell us nothing new about semantic knowledge representation in the brain. To address this issue, the authors could try to characterize and control for the effects of animacy (and the human-animal distinction within that) when measuring how well the model predicts behavior and/or the brain (i.e., regress this distinction out or focus their analysis on similarity matrices within these superordinate categories). If the effects survive, then this would indeed tell us that the models capture something interesting and novel about the semantic structure of neural representations and their connections with behavioral measures / perception, beyond animacy.

**[Response]** We appreciate the insightful comment. Following the comment, we tested the brain–behavior correlation of word dissimilarity matrices within each of the six categories (i.e., humans, non-human animals, non-animal natural things, constructs, vehicles, and other artifacts); we thought that this is a highly conservative manner to exclude the possibility that the behavioral correlates we observed were simply due to the animate/inanimate representational distinction. As a result, we observed significant brain-behavior correlations within each of the “humans”, “non-animal natural things”, “vehicles”, and “other artifacts” categories (please see S24 Fig in the revised manuscript). This result indicates that the observed brain-behavior correlation of word dissimilarity matrices cannot be simply explained by the animate/inanimate representational distinction. We have described this result in the Result section (lines 426–438).

**Minor Comments**

(4) line 145: point to the corresponding supplementary figure (S5?). Authors could also consider running a Friedman test to assess non-monotonicity statistically.

**[Response]** We guess that this comment points to S1 Fig in the original manuscript. Following this comment, we performed a statistical test for monotonicity on mean predication accuracy against vector dimension. Friedman test, suggested by the reviewer, is a non-parametric version of repeated-measure ANOVA, which would not be appropriate for assessing monotonicity. Instead, we used Kendall rank correlation to assess the monotonicity of prediction accuracy and significant-voxel fraction against vector dimensionality separately for different models and datasets. We found no significant rank correlation, indicating that the model performance was not monotonic. We have described this result in the Result section (lines 167–170).

(5) line 151: how do you define ‘appropriate’? It would be helpful if you mentioned briefly in the text that you used Freesurfer anatomical segmentation.

**[Response]** Following this comment, we have modified the description (lines 198–202) as follows:

*We next identified which cortical regions were predictable by the word vector-based models in order to determine that the models could capture the semantic representations in cortical regions which are considered to be involved in audiovisual semantic processing.*

(6) Fig. 4: it would be helpful to have labels for these brain regions in the caption / text / supplement. Is the yellow one in a/b LO?

**[Response]** Following this comment, we labeled the five brain regions with the highest prediction accuracy or the highest fraction of significant voxels for each model, dimension, and movie set in the figures, and described the names of these regions into separate tables. Even in this labeling, we observed highly consistent results across models, dimensions, and movie sets. Please see Figs 4–5, Tables 1–2, S5–7 and S10–12 Figs, and S1–3 and S7–9 Tables.

(7) Fig. 4: A voxel-wise map similar to Huth et al. 2012/2016 would be helpful here to compare how this method can model semantic information in the brain (i.e., same figure, but with voxel-level granularity, not just Freesurfer ROI).

**[Response]** A voxelwise map similar to Huth et al. 2012/2016 needs to be generated at the single-participant level. However, since our dataset contained a large number of participants (52 in total), we could not show voxelwise maps for all the participants. Instead, we generated a voxelwise map of prediction accuracy for one representative participant whose fMRI data were collected for both the movie sets. Please see S8 Fig.

(8) line 273: the effect sizes are really low (1-2% neural variance explained) and the authors are over-inflating the value of the procedure here.

**[Response]** We are afraid that we could not determine which description the reviewer referred to. The description in the line 273 in the original manuscript seems to be unrelated to the reviewer’s comment.

(9) It would be useful to discuss why you chose fastText over other models. This could also be addressed in conjuction with Major Comment #1 above.

**[Response]** We added GloVe and word2vec for the analysis, as described in the response to the reviewer’s comment #1. Hence, we decided that the discussion is unnecessary for the revised manuscript.

(10) Multiband factor 6 is quite high and the TR=1s is quite fast -- this may cause very low SNR (which maybe would explain low r values compared to similar work?). Is there a reason you did not use a more conventional fMRI sequence (e.g., multiband 2/4, 1.5-2s TR)?

**[Response]** We used the same fMRI parameters in our previous study (Nishida et al. 2020 AAAI 34:5281–5288) and observed a similar performance of brain decoding as in our different study (Nishida and Nishimoto 2018 NeuroImage 180:232–242), which used more conventional fMRI parameters (multiband factor 3; TR 2s). In addition, recent studies of other groups also used comparable or more advanced fMRI parameters (Greene et al. 2018 Nat Commun 9:2807; Son et al. 2019 Cereb Cortex 30:1171–1184; Korucuoglu et al. 2020 NeuroImage 214:116759); of them, the fMRI dataset used in Greene et al. (collected with multiband factor = 8 and TR = 0.72 s) was provided by the Human Connectome Project (Van Essen et al. 2013). Accordingly, we had decided to use the advanced fMRI parameters. Actually, our voxel-based analysis revealed that the model performance in this study was comparable with that in previous studies (Huth et al. 2012/2016) as described above. Thus, we do not think that the fMRI parameters used had a bad effect on our results.

(11) line 228: The authors should be a little more careful with the strength of the conclusions they draw from their results.

**[Response]** We agree with the reviewer’s suggestion. Actually, after adding the GloVe and Word2vec embedding models and changing the method for statistical testing (following another reviewer’s suggestion), we found a significant difference between brain– and word vector–behavior correlations only for the noun dissimilarity of fastText embedding models. Therefore, we withdrew the conclusion we made in the original manuscript. Instead, we have added the following neutral descriptions to the Result section:

Lines 309–313: *Although we also found significant correlations between the word vector- and behavior-derived matrices (word vector–behavior correlations; p < 0.0001), the brain–behavior correlations for nouns (but not for adjectives) were significantly stronger than the word vector–behavior correlations (p < 0.0001).*

Lines 349–351: *In the comparison between brain–behavior and word vector–behavior correlations, there was a significant difference only for fastText noun dissimilarity (p < 0.0001, FDR corrected).*

(12) line 310: I don’t agree with the argument that the language regions are not involved in processing this type of movie info – I suspect that an activation GLM analysis on the movie data would show weak, but consistent activation in language regions that the current model cannot capture.

**[Response]** We agree that “*the language regions are not involved*” would be overstated. We modified the statement to “*the language network has a relatively small contribution to semantic processing that requires no explicit linguistic processing*” (lines 513–515). Actually, this notion is rather consistent with our additional result that substantial brain–behavior correlations of word dissimilarity were observed in widespread cortical regions, including the language regions (please see Figs 9 and 10 in the revised manuscript).

(13) line 330: I don't necessarily agree that word embeddings are more explainable than deep network representations; they are just as opaque. I tried to find work supporting the authors’ claim but couldn’t. Please provide some references here and expand on this explanation or please reword.

**[Response]** We apologize for the misleading description. Here we would like to discuss the interpretability of internal representations of word embeddings and deep neural networks. Internal representations of word embeddings (i.e., word vectors) are directly associated with single words, which makes the representations interpretable for us. In contrast, the internal representations of deep neural networks (i.e., unit activation patterns and weights of intermediate layers) cannot be easily interpreted due to huge dimensionality and the indirect association with external information. Actually, such opacity of internal representations causes some cases of unnatural false recognition of deep neural networks (Szegedy et al. 2014 ICLR; Goodfellow et al. 2015 ICLR; Ribeiro et al. 2016 SIGKDD) and thereby the development of methods for improving the interpretability of deep neural networks is recently a hot topic in the field of machine learning (Valez and Kim 2017 arXiv; Guidotti et al. 2018 ACM Computing Surveys). To make this point clearer, we modified the description in the Discussion section (lines 539–542) as follows:

*Second, word vectors are easily interpretable owing to their direct association with the meaning of words. In contrast, deep learning features cannot be easily interpreted without sophisticated methods for improving their interpretability [45,46].*

**Response to Reviewer #2:**

I have now had a chance for an in-depth review of “Behavioral correlates of cortical semantic representations modeled by word vectors” by Nishida et al. The manuscript is well written and the overall problem domain is derived clearly. In this project, the authors use encoding models based on a FastText embedding space to predict fMRI brain data and subsequently use the predicted beta values to compute a representational dissimilarity matrix (RDM) for a novel set of words. The resulting RDM is compared to a behavioural RDM obtained via inverse MDS on the same words. Using this elaborate pipeline, the authors come to the conclusion that the predicted brain data and behavioural judgments are correlated, indicating the feasibility of using semantic embedding spaces for brain and behavioural modelling.

The overall approach is interesting, especially since the brain data was obtained via a visual/sensory modality, whereas the encoding model is derived from more abstract textual scene descriptions. To my understanding this cross-domain encoding approach acts as a “filtering” function that highlights brain regions related to semantics rather than low-level visual brain regions. This strength of the paper could have been highlighted a bit more. As stated above, the paper is well written, and computationally elegant. However, I have a few comments, as detailed below, which I hope will help the authors improve their work.

**Major 1: Take-home message and novelty**

While I see the elegance and computational complexity of the author’s endeavour, I am a bit lost as to what the actual take home message of the work may be and where and how the authors advance beyond what is known about the brain from prior work. I therefore invite the authors to elaborate on this.

At multiple occasions, the authors underline the novelty of the work, stating that “[…] there has been no study explicitly examining whether the modeled semantic representations actually capture our perception of semantic information.” and “However, whether the semantic representations modeled by word vectors accurately reflect the semantic perception of humans is yet to be determined.". However, there is a large body of research investigating exactly these questions (see [1-6] for examples of previous work most of which remain uncited).

**[Response]** We apologize for the ambiguous descriptions. The objective of the present study is to elucidate the behavioral correlations of ***brain*** semantic representations ***modeled using word vectors***, but not the behavioral correlates of word vectors themselves. Most of the previous studies the reviewer cited investigated the latter, the behavioral correlates of word vectors (or deep-learning representations; Iordan et al. 2020; Mikolov et al. 2013; Hill et al. 2015; Pereira et al. 2016; Peterson et al. 2018; i.e., [1]–[4] and [6] in the reviewer’s references). In addition, the remaining study (Bankson et al. 2018; i.e., [5] in the reviewer’s citation) examined the correlation between brain representations and word vectors (or deep-learning representations), which is also different from what we aimed to do.

Word vectors have been extensively used in neuroimaging studies to model semantic representations in the brain (e.g., Güçlü and van Gerven 2015; Nishida and Nishimoto 2018; Pereira et al. 2018). This indicates that word vector-based brain modeling is a potential tool for exploring semantic representations in the brain. However, no study has yet determined that brain semantic representations modeled by word vectors are associated with human behavior reflecting semantic perception. This association is not trivial even though word vectors themselves were previously reported to correlate with human behavior. Thus, our finding of the behavioral correlates of brain semantic representations modeled by word vectors is novel and important to establish an essential methodological basis for the existing and future research based on word vector-based modeling.

To make it clearer what is the difference in research objectives between ours and the previous studies, we have added descriptions in Abstract, Author summery, the Introduction section (paragraph 2 and 3), and the Discussion section (lines 468–469).

**Major 2: Technical validity**

Going through the manuscript, I found myself continuously wondering about the exact statistical procedures run for the different tests described. This made it quite difficult to judge the actual merit of the claims made. Below are examples of cases that need technical clarification/adjustments:

a. The authors write “the fraction of significant voxels that reach their prediction accuracy to a threshold value”. It remains unclear, however, how significance was established, what the threshold value was, what statistical test was used, which data was tested, etc. As a second example, the authors write “Only regions with mean prediction accuracy above 0.11 (corresponding to p = 0.0001 ~ 0.05/150 regions) are shown”. Can the authors clarify what exactly they did here to derive the statistics?

**[Response]** We apologize for the insufficient descriptions. For calculating the fraction of significant voxels, we first evaluated the statistical significance of prediction accuracy for each voxel. Since the prediction accuracy corresponded to the Pearson’s correlation coefficient between predicted and measured fMRI signals in each voxel, we obtained a P value for each voxel. Then, we corrected the P values for all cortical voxels of each participant using FDR for the correction for multiple comparisons. Finally, the fraction of significant voxels was calculated using the rate of voxels, to all cortical voxels, for which the corrected P values reached a significance threshold (P < 0.05). To make this procedure clearer, we have modified the description in the Results section (lines 129–131) as follows:

*(2) the fraction of significant voxels, among all cortical voxels, for which the prediction accuracy reached a significance threshold (p < 0.05 after the correction for multiple comparisons using the false discovery rate [FDR]; for more details, see Methods).*

We have also added the following description in the Methods section (lines 716–721):

*In addition, the significance of prediction accuracy (Pearson’s correlation) for each of all cortical voxels was evaluated after the correction for multiple comparisons using FDR. The rate of voxels, to all cortical voxels, for which the prediction accuracy reached a significance threshold (p < 0.05, FDR corrected) was calculated as the fraction of significant voxels, which was another measure of brain-response prediction performance for each model.*

For determining the threshold of prediction accuracy (=0.11), we used P values corresponding to prediction accuracy (Pearson’s correlation coefficients), assuming that the prediction accuracy was calculated for a single brain model. In addition, the P values obtained for all of 148 cortical regions (sorry, but not 150 regions; please see the response to the reviewer’s minor comment #1) were corrected for multiple comparisons with Bonferroni correction; specifically, the standard significance threshold of P = 0.05 was divided by 148 regions. This procedure yielded the corrected significance threshold of P = 0.0001 (~ 0.05/148) and the corresponding threshold of prediction accuracy (=0.11). Nevertheless, please note that the threshold of prediction accuracy was used only for visualization purposes and did not affect our conclusion. To make this procedure clearer, we have modified the description in the caption of Fig 4 as follows:

*We showed only regions with mean prediction accuracy above 0.11, which reaches a significance level (i.e., p = 0.05) of prediction accuracy after Bonferroni correction for multiple comparisons among 148 cortical regions (i.e., p = 0.0001 ~ 0.05/148).*

b. The minimum fraction of significant voxels within any individual region is reported as 0.237. This seems very high, given that the average model prediction accuracy across all voxels is reported as “0.09”. To better understand this discrepancy, I would like the authors to comment on (a) how exactly the test set was derived, (b) how exactly the authors tested for significance (see above comment), and (c) how many training/test

datapoints were used. Secondly, testing against chance, after having fitted hundreds of free parameters, seems like a somewhat low bar (although I acknowledge the fact that this is prediction on test data). As a more conservative control, I suggest the authors use an untrained embedding network as a control model as this would give the claims for actual semantic embedding throughout the cortex more explanatory power/merit.

**[Response]** We apologize for the ambiguous descriptions. In response to (a), the test dataset for each movie set was derived from fMRI responses to two movie clips (1200 s) each of which was presented four times in four separate scans, as described in the Method section (4.2) of the original manuscript. The fMRI responses were averaged over repetitions to improve the signal-to-noise ratio. Similar experimental designs were used in many other studies on encoding/decoding modeling (e.g., Nishimoto et al. 2011 Curr Biol; Huth et al. 2012 Neuron; Stansbury et al. 2013 Neuron; Huth et al. 2016 Nature; Nishida and Nishimoto 2018 NeuroImage; Wen et al. 2018 NeuroImage; Çelik et al. 2018 NeuroImage). In response to (b), as described above (the response to the major comment #2-a), the calculation of prediction accuracy (Pearson’s correlation between predicted and measured brain responses in the test dataset) produced the corresponding P values. Then, the P values were corrected over all voxels for each participant using FDR. In response to (c), for each movie set, we used 7200 datapoints (derived from 7200-s movies) for training and separate 1200 datapoints (derived from 1200-s movies) for test. To make these methods clearer, we have added the following descriptions in the Method section:

Lines 593–594: *The fMRI responses to these clips were then averaged across four scans to improve the signal-to-noise ratio [11,23,53]*

Line 705: *The model training was conducted using the training dataset (7200 datapoints).*

Lines 713–714: *The brain-response prediction performance of the model was evaluated using the test dataset (1200 datapoints), …*

Lines 716–721 (please see the response to the major comment #2-a)

We interpreted the reviewer’s suggestion regarding the control model as meaning that the fraction of significant voxels within each cortical region should be compared between original and control models, but not that the significance of prediction accuracy for each voxel should be tested using the comparison between original and control models. This is because each of the original and control models produces only a single value of prediction accuracy for each voxel and thereby the voxelwise significance test of prediction accuracy cannot be performed. According to this interpretation, we emulated an untrained embedding network proposed by the reviewer using word vectors for which the vector representations were shuffled across vector dimensions for each word. Then, we constructed voxelwise models using scene vectors generated from the dimensionally shuffled word vectors and compared the fraction of significant voxels for these control models with that for the original models. The result revealed that the fraction of significant voxels for each cortical region was much lower for the control models (mean values = 0.005–0.060) compared with the original models (mean values = 0.522–0.587; Wilcoxon test, p < 0.00001). Thus, even considering the comparison with the control models, the fraction of significant voxels for the original models seems sufficiently high in every cortical region. To show these results, we have added a new figure (S13 Fig) and new descriptions (lines 271–276 and 723–737).

c. “There was a strong correlation of mean prediction accuracy over 150 cortical regions between movie sets 1 and 2 (Pearson’s r = 0.974);“ Please repeat this analysis with a robust Spearman correlation, as deviations from normality (such as outliers) can easily increase the Pearson correlation coefficient but this is not meaningful. Please note that nearby regions are autocorrelated, which may violate the independence assumption of the classic Pearson statistic.

**[Response]** Following this comment, we calculated the Spearman’s correlation of mean prediction accuracy between movie sets and found a similar strong correlation for 1000-dimensional fastText vectors (Spearman’s ρ = 0.974). Such high Spearman’ s correlation was also observed for the other types of word vectors (i.e., GloVe and word2vec), for the fraction of significant voxels, and in the correlation between different vector dimensions (Spearman’s ρ > 0.95). This result indicates that the observed strong correlation is not specific to Pearson’s correlation and, again, the localized patterns of model performance over 148 cortical regions are highly consistent across datasets and vector dimensionality. To show these results, we have described these results in the Results section (lines 208–210) and S4–6 and S10–12 Tables.

d. To my understanding, the significance of correlations between RDMs (Figure 5) is computed via bootstrapping individual RDM cells. I do not think that this is technically valid, as the RDM cells have dependencies that are violated by this approach (the N*N-1 cells are all derived from only N experimental conditions). The more standard approach, which I advise the authors to take, would be to perform a permutation test on the RDM conditions (i.e. permuting the rows/columns of one of the two RDMs to derive a null distribution against which the empirical correlation value can be tested). Moreover, Spearman correlations are more commonly used for RDM comparisons across domains.

**[Response]** We appreciate the helpful suggestion. Following the suggestion, we replaced bootstrapping test with permutation test. As reviewer proposed, a null distribution was obtained by permuting the rows of either RDM and used to test the significance of correlation values. In addition, we also evaluated the behavioral correlations using Spearman’s correlation coefficients. To show the results, we have added/modified the figures (Figs 6–10 and S16–25 Figs) and added descriptions into the Methods section (lines 827–830 and 835–846).

**Minor:**

1. Which exact atlas was used? This is not mentioned anywhere. Relatedly, were the 150 regions tested all regions of the atlas, or were ROIs excluded?

**[Response]** We apologize for the unnoticeable description. This paper used the Destrieux atlas, which is one of the standard cortical atlases (Destrieux et al. 2010 NeuroImage). We also apologize for the wrong description. Although this atlas included 150 cortical regions, we tested only 148 regions of them because the remaining 2 regions (bilateral medial wall) contained no voxel for every participant. To describe these, we have modified descriptions in the Result section (lines 199–202) as follows:

*For this purpose, the prediction accuracy averaged across participants was calculated in each of the 148 cortical regions that were anatomically segmented using FreeSurfer [24,25] on the basis of the Destrieux atlas [26].*

We have also modified descriptions in the Method sections (lines 605–606) as follows:

*Then, each voxel was assigned to one of the 148 cortical regions derived from the Destrieux atlas for cortical segmentation [26].*

2. Please consider citing [7], which is a novel paper in the domain.

**[Response]** Following the comment, we have added the following description to the Discussion section (lines 486–492):

*This enables to comprehensively understand the representational structure of semantic information in the human brain. For example, a recent study demonstrated the multidimensional representations of natural objects on the basis of a data-driven approach using large-scale behavioral data of similarity judgements [33]. The word vector-based models capturing human word similarity judgements may be used to explore whether such a multidimensional structure of semantic representations actually exists in the human brain.*

3. Could the authors attempt a spatial localization of brain-behavioural semantic processing by computing RDMs for each ROI and correlate it with the behavioural data?

**[Response]** We appreciate the insightful suggestion. Following the suggestion, we evaluated the brain–behavior correlation of word dissimilarity within each of all the cortical regions. Interestingly, our results revealed that substantial brain–behavior correlation (up to 0.621) was observed in widespread cortical regions, including the prefrontal cortex, except for low-level audiovisual regions, such as the posterior occipital and superior temporal areas. This localization pattern of brain–behavior correlation coefficients appears to be different from that of highly predictable regions observed in the prediction-accuracy analysis (Fig 4). However, in the analysis of the fraction of significant voxels, we observed that the fraction was sufficiently high across the cortex (Fig 5, S10–12 Figs). These results suggest that our word vector-based voxelwise models capture semantic information even in high-level cortical regions, in which the semantic information is more intermingled with other types of information (this might produce the low prediction accuracy of our models) but is more associated with human perception than the semantic information in low-level cortical regions. This notion is supported by previous findings on the difference in information representation between high- and low-level cortical regions (Sakai and Passingham 2006 J Neurosci; Boly et al. 2013 Front Psychol; Cole et al. 2016 Cereb Cortex). To show these results and describe this notion, we have added new figures (Figs 9 and 10) and tables (S15–16 Tables) and modified the description in the Results (lines 393–407) and Discussion (lines 519–529) sections.

I sincerely hope that the above comments will be perceived as constructive and that they may help assist the authors in making their contribution stronger. It is an exciting project and overall computational approach.

[1] https://arxiv.org/pdf/1910.06954.pdf

[2] Mikolov, T., Yih, S. W. & Zweig, G. Linguistic Regularities in Continuous Space Word Representations. In Proceedings of the 2013 Conference of the North American Chapter of the Association for Computational Linguistics: Human Language Technologies, 746-751 (2013).

[3] https://arxiv.org/abs/1408.3456

[4] Pereira, F., Gershman, S., Ritter, S. & Botvinick, M. A comparative evaluation of off- the-shelf distributed semantic representations for modelling behavioural data. Cogn. Neuropsychol. 33, 175–190 (2016).

[5] https://pubmed.ncbi.nlm.nih.gov/29777825/

[6] https://arxiv.org/pdf/1805.07644.pdf

[7] https://www.nature.com/articles/s41562-020-00951-3

**Response to Reviewer #3:**

Although a number of studies demonstrated correspondences between brain activity and word embeddings, the behavioral validation of these studies has been lacking. The authors address this issue by using representational similarity analysis on behavioral data and fMRI data in order the compare the two, revealing a significant correlation. It is a clearly written manuscript with well-designed experiments and analyses. I really liked that it has a clear message and delivers it well without going into tangential research questions which would distract from the main results. I would be happy to recommend accepting the manuscript for publication after the few minor issues that I list below are addressed.

- It is not immediately clear how many participants were involved in the study and which tasks each participant performed. Although this information is given later in the methods section, it appears rather late in the manuscript. A table or a few sentences to clearly state this earlier in the manuscript could make it easier for the reader. On another note, just out of curiosity, I see that the number of participants was quite large in comparison to what we are used to seeing in similar studies. Was there a specific motivation for collecting such a large fMRI dataset?

**[Response]** Following the comment, we have described the number of participants in the Introduction section (lines 81–84) as follows:

*For this purpose, we conducted two sets of functional magnetic resonance imaging (fMRI) experiments in which 52 participants were asked to view natural movies in the scanner; among them, 36 participated in on or the other of these experiments, and 16 participated in both.*

As mentioned by the reviewer, our fMRI dataset contained the much larger number of participants than used in previous similar studies. Drawing on the large-scale dataset, we plan to explore individual differences of brain representations that are comprehensively quantified for each brain using voxelwise modeling. Please look forward to our future progress.

- Not a big deal, but in Figure 4, the colors that are overlaid on to the brain surfaces are transparent, which makes it a bit confusing to read the results with the brain surface also having brightness differences. Making the results opaque would be preferable, in my opinion. At the same time, I see the importance of conveying the anatomical information to the reader, so I leave it to the authors to decide what to do with this comment.

**[Response]** Following this comment, we made the overlaid colors opaque. In addition, we labeled the cortical regions with the highest model performance in each figure to provide the anatomical information of the predictable cortical regions to the readers. Please see Figs 5–6 and 9–10 and S5–7, S10–12, and S14–15 Figs.

- How was the variability of the RDMs between participants? Have the analyses been performed also at the individual level (e.g. comparing each participant’s behavioral data with their own brain data)? If not, why not?

**[Response]** We appreciate the helpful suggestion. Following the suggestion, we examined the brain–behavior correlation of word dissimilarity for each of the six participants who had both brain and behavioral data. We found significant brain–behavior correlation of noun dissimilarity for all the participants but significant correlation of adjective dissimilarity for only three participants. This result may suggest that the current version of word vector-based voxelwise models cannot capture the individual variability in adjective dissimilarity perception. However, there is another possibility that the psychological experiments for adjective arrangement are not sufficient to capture the variability of adjective dissimilarity perception at the individual level (but not at the population level). Further investigation will be needed to determine them. To show these results, we have added a new figure (S20 Fig) and a new description (lines 361–364).
