# Supplementary material for: Behavioral correlates of cortical semantic representations modeled by word vectors
Source: PLoS Comput Biol. 2021 Jun 23;17(6):e1009138. doi: 10.1371/journal.pcbi.1009138 (PMC8260002; doi:10.1371/journal.pcbi.1009138)
Supplement: Attachment — Submitted filename: Nishida-BehavCorrSemantic-ResponseLetter04.docx [file pcbi.1009138.s047.docx]

We appreciate the reviewer for his efforts in helping us to improve this paper. We have revised the manuscript and figures to address all of his concerns. Our point-by-point replies are given here.

**Response to Reviewer #2:**

**Minor:**

1. In response to one of my earlier comments, the authors derived a random control model (“we emulated an untrained embedding network […] using word vectors for which the vector representations were shuffled across vector dimensions for each word.). This approach seems overly lenient, as no sensible betas can be derived for the encoding model if the dimensions are shuffled randomly for each datapoint. I therefore do not see the immediate use in this control. What I had originally thought of, but perhaps not communicated precisely enough, was that the authors take an untrained DNN (rather than a semantics trained DNN) and run it through their pipeline. This ensures that the encoding model can make “sensible” predictions, while the model activation patterns themselves are not driven based on semantics, as the model is untrained.

We apologize for our misunderstanding of the reviewer’s earlier comment. We agree that the brain-response prediction from the encoding model based on untrained word vectors does not use the semantic relational structure of words, which is captured by original, trained word vectors. However, we do not agree that the prediction is not driven based on semantics at all, because untrained word vectors also have signatures of individual words (even if they are drawn from random vectors) and produce scene vectors that implicitly reflect the information about which words are contained in individual scene descriptions. In other words, the encoding model based on untrained word vectors is basically similar to the binary-labeling model, which was introduced as an encoding model based on discrete semantic features and exhibited comparable or higher brain-response prediction performance compared with the original word vector-based models (S4 Fig). Therefore, the encoding model based on untrained word vectors would not be suitable for a control model for comparing semantic and non-semantic models in terms of brain-response prediction.

Nevertheless, comparing trained and untrained word vectors in encoding modeling is rather beneficial to determine whether the semantic relational structure of words (but not the semantic information of individual words themselves) is effective in the modeling of cortical semantic representations (although this may be the reason why the reviewer suggested this comparison). This comparison is fairer than the comparison between the original word vector-based and binary-labeling models, which employed different procedures for transforming scene descriptions to semantic features. Higher model performance of trained word vectors relative to untrained word vectors purely indicates that the semantic relational structure of words, captured by trained word vectors, is effective for the modeling of cortical semantic representations. For this reason, we compared trained and untrained word vectors in terms of model performance of brain-response prediction, including not only the region-wise analysis but also the whole-brain analysis of prediction performance.

We found that the models based on trained word vectors significantly outperformed the models based on untrained word vectors in all the analyses: the whole-brain analysis of prediction accuracy (Wilcoxon test, p < 0.00001, FDR corrected) and significant-voxel fraction (p < 0.00001, FDR corrected) and the region-wise analysis of prediction accuracy (p < 0.00001, FDR corrected) and significant-voxel fraction (p < 0.00001, FDR corrected). The region-wise analysis also revealed that the improvement of model performance for trained word vectors was uniform across cortical regions rather than being conspicuous in specific regions. These results indicate that the semantic relational structure of words, captured by word vectors, is effective in the modeling of semantic representations in widespread cortical regions. To show these findings, we have added new figures (S5 and S17 Figs) and descriptions in the Results and Methods sections (lines 196–219, 311–325, and 779–800). In addition, we have removed the following sentence from the descriptions of the shuffled-vector analysis in the Methods section: “*These shuffled word vectors could be interpreted as vectors derived from an untrained word-vector space*” (line 770).

2. Typo: Author summery —> Author summary

Following the comment, we have corrected the typo (line 40).

3. Figure 7 - would it make sense for the authors to add the binary category control model as an additional bar?

As the reviewer suggested, it might be informative for readers to show the behavioral correlation for the binary-labeling control model in Fig 7. However, the number of words used for evaluating the behavioral correlation for the binary-labeling model (please see S18 Table) was different from that for the word vector-based models in Fig 7 (i.e., all the 60 nouns or adjectives). To avoid readers’ confusion, we had separately performed the comparison of these models using the same subsets of words and showed the results in a separate figure (S27 Fig in the revised manuscript).

4. I would advise not calling binary noun/category descriptions “primitive” (l 678).

Following the advice, we have removed the word “*primitive*” (line 719). Likewise, we have replaced the terms “*more primitive, discrete word features*” with the terms “*simpler, discrete word features*” (line 172).
